# A network meta-analysis of efficacy and safety for first-line and maintenance therapies in patients with unresectable colorectal liver metastases

**DOI:** 10.3389/fphar.2024.1374136

**Published:** 2024-07-26

**Authors:** Yunlin Jiang, Taihang Shao, Mingye Zhao, Yahong Xue, Xueping Zheng

**Affiliations:** ^1^ Nanjing Hospital of Chinese Medicine Affiliated to Nanjing University of Chinese Medicine, Nanjing, China; ^2^ Graduate School of Nanjing University of Chinese Medicine, Nanjing, China; ^3^ Department of Pharmacoeconomics, School of International Pharmaceutical Business, China Pharmaceutical University, Nanjing, China

**Keywords:** metastatic colorectal cancer, unresectable liver metastases, network meta-analysis, firstline treatment, maintenance treatment

## Abstract

**Background:** Evidence comparing the efficacy of different treatments for patients with unresectable colorectal liver metastases (CRLM) receiving first-line or maintenance therapy is sparse. We aimed to assess the efficacy and safety of these treatments, with a distinct focus on evaluating first-line and maintenance treatments separately.

**Methods:** We conducted Bayesian network meta-analyses, sourcing English-language randomized controlled trials (RCTs) published through July 2023 from databases including PubMed, Embase, the Cochrane Library, ClinicalTrials.gov, and key conference proceedings. Phase Ⅱ or Ⅲ trials that assessed two or more therapeutic regimens were included. Primary outcome was overall survival (OS). Secondary outcomes included progression-free survival (PFS), objective response rate (ORR), adverse events graded as 3 or above (SAE), and R0 liver resection rate. Hazards Ratios (HRs) and 95% confidence intervals (CI) were used as effect size for OS and PFS, Odds Ratios (ORs) and 95% CI were used for ORR, SAEs and R0 resection rate. Subgroup and sensitive analyses were conducted to analysis the model uncertainty (PROSPERO: CRD42023420498).

**Results:** 56 RCTs were included (50 for first-line treatment, six for maintenance therapies), with a total of 21,323 patients. Regarding first-line, for OS, the top three mechanisms were: local treatment + single-drug chemotherapy (SingleCT), Targeted therapy (TAR)+SingleCT, and TAR + multi-drug chemotherapy (MultiCT). Resection or ablation (R/A)+SingleCT, S1, and Cetuximab + intensified fluorouracil-based combination chemotherapy (ICTFU) were identified as the best treatments. For PFS, the top three mechanisms were: Immune therapy + TAR + MultiCT, multi-targeted therapy (MultiTAR), TAR + SingleCT. The top three treatments were: Atezolizumab + Bevacizumab + fluorouracil-based combination chemotherapy (CTFU), TAS-102+bevacizumab, Bevacizumab + ICTFU. Cetuximab + CTFU was the best choice for RAS/RAF wild-type patients. Regarding maintenance treatment, Bevacizumab + SingleCT and Adavosertib were the best options for OS and PFS, respectively. For safety, MultiCT was the safest, followed by local treatment + MultiCT, TAR + MultiCT caused the most SAEs. Bevacizumab plus chemotherapy was found to be the safest among all targeted combination therapies.

**Conclusion:** In first-line, local treatment or targeted therapsy plus chemotherapy are the best mechanisms. R/A + SingleCT or CTFU performed the best for OS, Atezolizumab + Bevacizumab + ICTFU was the best option regarding PFS. For RAS/RAF wild-type patients, Cetuximab + CTFU was the optimal option. Monotherapy may be preferred choice for maintenance treatment. Combination therapy resulted in more SAEs when compared to standard chemotherapy.

## 1 Background

Colorectal cancer (CRC) is a common malignant digestive tract tumor, and in recent years, its incidence and mortality rates have shown an increasing trend year by year. The global incidence of CRC has been escalating, experiencing yearly growth rates of 3.2% (Zhou et al., 2022).Worldwide, it is the second leading cause of cancer-related mortality and ranks as the third most common cancer (Wang et al., 2023). Approximately 15%–25% of patients are found to have colorectal liver metastases (CRLM) at their initial diagnosis, while 70%–80% of patients with CRLM are initially deemed unresectable. For resectable patients, 50%–60% may experience recurrence after surgery, potentially progressing to unresectable disease (Xu et al., 2019).

When suspected liver metastasis is found in clinical examinations, it is recommended to perform liver-enhanced magnetic resonance imaging (MRI) scans. For initial unresectable CRC, it is recommended to test the patient’s gene statuses. For patients with unresectable CRLM, conversion therapy can be considered after multidisciplinary team discussion, with preoperative chemotherapy or chemotherapy combined with targeted drugs recommended. Whether the primary lesion of CRLM, without bleeding or obstruction symptoms, should be removed is still under debate (NCCN Guidelinesa; NCCN Guidelinesb; Scherman et al., 2021). A multicenter prospective study showed that there was no statistically significant difference in overall survival (OS) between patients with primary synchronous CRLM who underwent resection of the primary lesion tumor followed by systemic chemotherapy and those who only received chemotherapy (Park et al., 2020). For most LM that cannot be surgically removed, radiofrequency or microwave ablation can be used to control local lesions. Transarterial chemoembolization (TACE) is an effective minimally invasive treatment that is widely used for unresectable CRLM. However, TACE-induced hypoxia microenvironment and increased neovascularization may potentially promote early progression (Fiorentini et al., 2018). Systemic therapy is a preferable treatment choice for unresectable CRLM due to its ability to improve both quality-of-life and survival. Furthermore, effective systemic therapy—which includes chemotherapy, targeted therapy, and other systemic treatments—has the potential to convert unresectable lesions into resectable ones (Tomasello et al., 2017). FOLFOX, CAPEOX, FOLFIRI, and 5-fluorouracil/leucovorin or capecitabine are the recommended initial chemotherapy treatments for eligible patients who require intensified therapy. Effective cancer therapy enables about 12.5% of patients with unresectable CRLM to undergo liver resection and consequently improves their survival rates, however, it is essential to carefully consider the potential adverse effects (AEs) associated with this regimen (Adam et al., 2004). Patients who are able to tolerate aggressive therapy may experience improved outcomes by combining chemotherapy with targeted therapy. The combination of chemotherapeutic drugs is commonly used along with drugs that target epithelial growth factor receptor (EGFR) and VEGF (Li et al., 2014). Cetuximab and panitumumab are frequently used as EGFR inhibitors, while bevacizumab plays a vital role in anti-angiogenesis by targeting VEGF (Xie et al., 2020). Nevertheless, the response to anti-angiogenic therapy differs among patients, whereby some individuals do not experience any benefits, while others may develop tolerance or encounter more severe consequences (Abdalla et al., 2018; Lugano et al., 2020). Blocking immune checkpoints directly to prevent immune escape is the most established approach in immunotherapy, which has shown outstanding efficacy in treating various types of cancer (Hoos et al., 2010). The patients’ response to immune checkpoint blockade (ICB) varies depending on whether they have DNA microsatellite instability (MSI) or mismatch repair (MMR) status, unlike patients with other types of cancer (Johdi and Sukor, 2020).

At present, there are numerous treatment choices accessible for unresectable CRLM patients who have not received treatment or receive maintenance therapies, caused by various mechanisms. However, there is insufficient information regarding the comparative outcomes of these options. As a result, we undertook this study to comprehensively evaluate the influence of all current treatment regimens on the survival outcomes of patients with unresectable CRLM receiving first-line or maintenance therapies. Our objective was to determine the relative efficacy and safety of these regimens and provide healthcare clinicians, patients, and relevant guidelines with valuable references for clinical medication and disease management.

## 2 Methods

Our study was conducted following the guidelines of the Preferred Reporting Items for Systematic Reviews and Meta-Analyses (PRISMA) extension statement (Hutton et al., 2015). See Supplementary File S1. This systematic review protocol was registered on PROSPERO (CRD42023420498).

### 2.1 Data sources and search strategy

The search strategy is provided in Supplementary File S2. In 31 July 2023, we conducted a comprehensive search on PubMed, EMBASE, Cochrane Library, and ClinicalTrials.gov to find relative RCTs and published studies. There were no restrictions on the publication date, and language was limited to English. Moreover, abstracts from the European Society for Medical Oncology, American Society of Clinical Oncology since 2021 were also included in the search.

### 2.2 Selection criteria

Two researchers (YJ and TS) independently screened all articles identified through the database search by title and abstract. Articles that met the inclusion criteria were then subjected to full-text screening. Discrepancies were sorted out through discussions involving other researchers (YJ, TS, MZ, YX, and XZ). The eligibility criteria based on the PICOS framework were as follows:

(1) Population: Adult patients with confirmed CRLM, diagnosed either histologically or cytologically. Patients also need to meet the requirement of receiving first-line treatment or maintenance treatment after prior system treatment stabilization. No limitations were imposed regarding individual-level characteristics. Due to the fact that some RCTs only reported results for mCRC patients with 2 or more organ metastases, we assumed that such patients had liver metastasis, considering liver metastasis is present in more than 90% of these patients (Riihimäki et al., 2016; Reboux et al., 2022).
(2) Interventions and comparisons: We evaluated any systematic interventions, including pharmaceutical, surgical, radiological, and combination therapies.
(3) Outcomes: The trials included in the analysis reported on clinical outcome measures such as OS, progression-free survival (PFS), objective response rate (ORR), AEs graded as 3 or above (SAE) according to the National Cancer Institute Common Terminology Criteria for Adverse Events, and R0 liver resection.
(4) Study design: Phase Ⅱ or Ⅲ studies that compared multiple distinct treatments were primarily considered.

To avoid redundancy, we focused on trials that provided the most recent and significant insights. Moreover, we dismissed trials that explored treatments unrelated to any comparisons. Additionally, trials that specifically investigated varying dosages but implemented the same administrations were also eliminated.

### 2.3 Data extraction and quality assessment

Two independent researchers (YJ and TS) were responsible for extracting the required data. The extracted information encompassed the characteristics of eligible trials (publication year, registration information, etc.), characteristics of populations (age, sample size, countries, etc.), and characteristics of the program (interventions, outcomes of endpoints, etc.). The clinical outcomes extracted included OS, PFS, ORR, SAEs, and R0 liver resection. For studies that only published Kaplan-Meier curves without providing hazard ratios (HRs) or a 95% confidence interval (CI), Liu et al.'s tool was used to extract OS or PFS rates, and number-at-risk from Kaplan-Meier curves. Individual patient data (IPD) were then reconstructed, and HR and their 95% CI were calculated based on reconstructed IPD (Liu et al., 2021).

Cochrane Collaboration’s risk of bias (ROB) tool was used to evaluate the quality of the studies included (Higgins et al., 2011). The eligible studies were categorized into three groups: high, low, or unclear risk (Lin and Chu, 2018). To evaluate the publication bias, the Egger regression test was utilized, with *p*-values <0.05 being interpreted as evidence of bias.

### 2.4 Statistical analyses

The primary outcomes analysed are OS and PFS, while the secondary outcomes included ORR and SAE, as well as R0 liver resection. Network plots were created to compare and visually represent the different treatment options. Pooled hazard ratios (HRs) with 95% CI were computed for OS and PFS. Pooled odds ratios (ORs) with 95% CI were calculated for ORR, SAE, and R0 resection rate. The analysis of synthesized HRs or ORs utilized the Bayesian approach, taking into account that the majority of direct evidence stemmed from a single trial. Therefore, the fixed effects consistency model was chosen (Zhao et al., 2019). The Bayesian network meta-analysis (NMA) using the R statistical packages Gemtc was carried out by employing four sets of Markov chains. Each set consisted of 50,000 samples with 10,000 burn-in samples. Non-informative prior distributions were utilized: specifically, a uniform prior distribution (Uniform (0, 1)) was used for parameter theta, and a normal prior distribution (Normal (0, 10^6)) was used for parameter mu (Sutton et al., 2008). In addition, we calculated the probability ranking for each available treatment and represented it using the surface area under the cumulative ranking (SUCRA). A higher SUCRA value indicated a greater rank.

The I^2^ statistic was used to evaluate heterogeneity among studies, with a value greater than 50% indicating a moderate level of heterogeneity (Zhao et al., 2019). The edge-splitting method was used to assess the inconsistency of models, taking into account direct and indirect evidence (Zhao et al., 2019). To ensure the robustness of this study, several comparisons were conducted using pairwise meta-analysis. To confirm the convergence of Markov chains, trace plots and Gelman-Rubin diagnostic statistics were utilized (Brooks and Gelman, 1998).

To assess the influence of the number of metastatic organs, subgroup and sensitivity analyses were conducted to evaluate the dependability of the findings. We classified the population receiving first-line treatment into two categories: those with liver-limited metastasis and those with multiple metastasis sites, and then conducted subgroup analysis separately. Due to limited evidence for patients receiving maintenance treatment, we chose not to classify this group. In the sensitivity analyses, we studied the potential impact of mutation target levels on the efficacy of intervention strategies.

## 3 Results

### 3.1 Characteristics of the included studies

**FIGURE 1:**
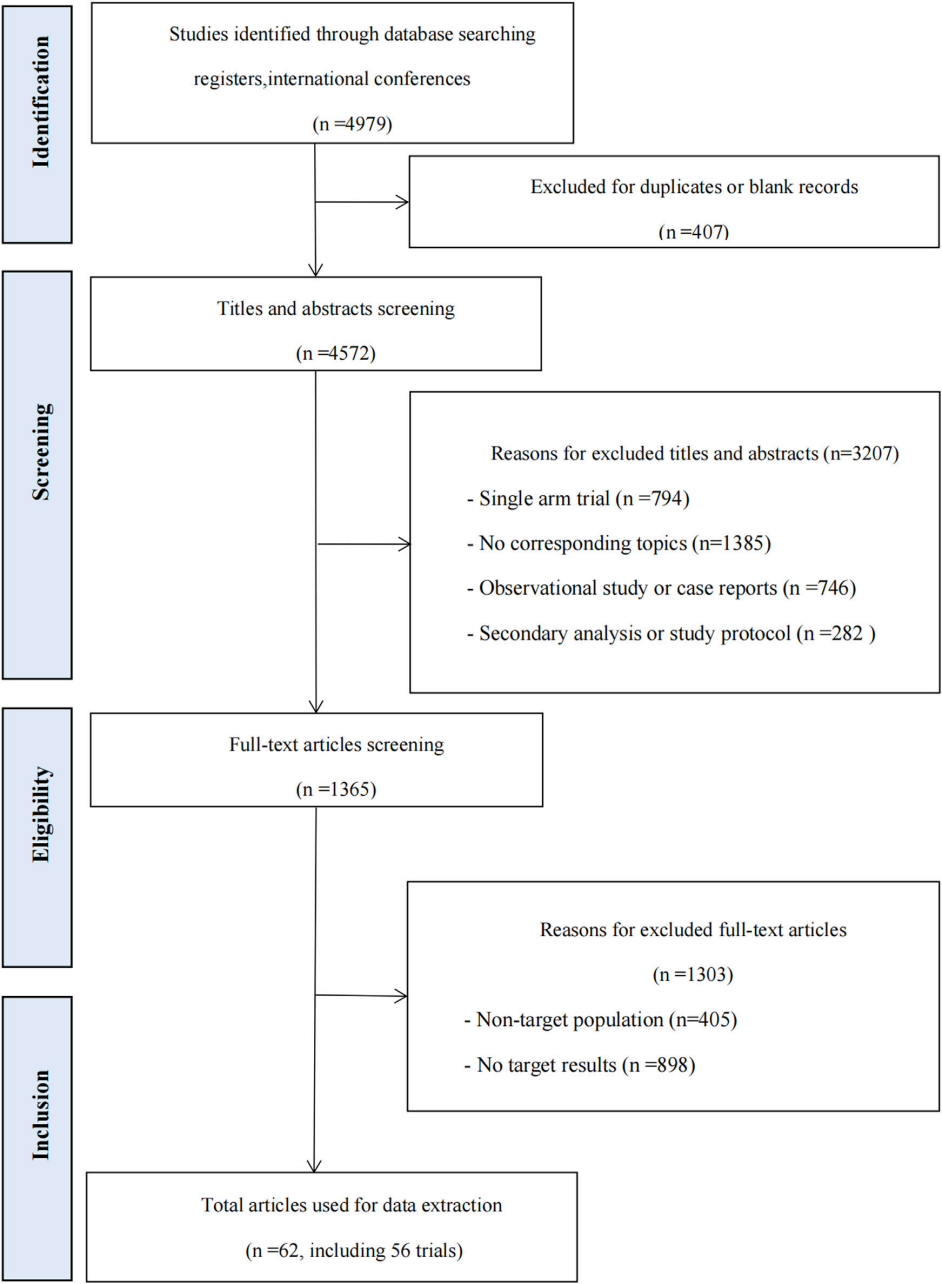
Study flow chart.

A total of 4979 records were identified from database searches. 3614 records were excluded during the title and abstract screening, and 1365 records were screened in full text. Sixty-two articles, comprising 56 RCTs, were included in the review. The flow chart can be seen in Figure 1. Details about the included studies are presented in Supplementary File S3.

This research study included a total of 21,323 patients diagnosed with metastasis CRC (mCRC). Of these, 50 RCTs are applicable for the comparative analysis of first-line treatment, and six RCTs were specifically designed for maintenance treatment. To form a complete indirect comparison, we classified chemotherapy into single-drug chemotherapy (SingleCT), fluorouracil-based combination chemotherapy (CTFU), capecitabine-based combination (CTCA), and intensified CTFU (ICTFU) which contained four drugs. We uniformly referred to the best supportive treatment, observation, or placebo as BSC without distinction. Briefly, 12 mechanisms have been included, including: BSC, immune checkpoint inhibitor (ICI)+multi-drug chemotherapy (MultiCT), ICI + targeted therapy (TAR)+MultiCT, local treatment (defined as hepatic artery infusion [HAI], Selective internal radiation therapy [SIRT], transcatheter arterial chemoembolization [TACE], resection or ablation [R/A]), local treatment + MultiCT, local treatment + SingleCT, MultiCT, multi-targeted therapy (MultiTAR), RNA therapy + TAR + MultiCT, SingleCT, TAR + MultiCT, TAR + SingleCT. Besides, 29 treatments were involved, comprising of Adavosertib, Atezolizumab + Bevacizumab + ICTFU, Bevacizumab, Bevacizumab + CTCA, Bevacizumab + CTFU, Bevacizumab + Erlotinib, Bevacizumab + ICTFU, Bevacizumab + SingleCT, Cediranib + CTCA, Cetuximab + CTFU, Cetuximab + ICTFU, CTCA, CTFU, CTFU + SIRT, GOLFIG (defined as combination of gemcitabine, oxaliplatin, levofolinate, 5-fluorouracil, granulocyte-macrophage colony-stimulating factor, and interleukin-2), ICTFU, R/A + CTFU, R/A + SingleCT, panitumumab + CTFU, panitumumab + ICTFU, pelareorep + bevacizumab + CTFU, S1 (combined chemotherapy of tegafur, gimeracil, and oteracil), S1+bevacizumab + singleCT, S1+singleCT, SingleCT, sorafenib + CTFU, TAS-102 (trifluridine/tipiracil) + bevacizumab, tivozanib + CTFU, TSU-68 (Orantinib) + S1 + singleCT. We did not classify capecitabine and fluorouracil as the same drug category. Despite similar mechanisms, their differences in clinical application and side effect management warranted separate analyses (Twelves et al., 2005; Cassidy et al., 2008). This yielded more accurate results and better supported clinical decisions.

### 3.2 Risk of bias

The assessment of ROB is presented in Supplementary File S4. Overall, ROB in all RCTs was generally low. However, multiple RCTs were open-label in our study (Cassidy et al., 2008; Ducreux et al., 2011; Hoff et al., 2012; Hong et al., 2012; Ruers et al., 2012; Yamada et al., 2013; Correale et al., 2014; Heinemann et al., 2014; Lee et al., 2014; Loupakis et al., 2014; Gruenberger et al., 2015; Simkens et al., 2015; Tournigand et al., 2015; Benson et al., 2016; Luo et al., 2016; Stintzing et al., 2016; Aparicio et al., 2018; Jonker et al., 2018; Qin et al., 2018; Yamada et al., 2018; Aranda et al., 2020; Cremolini et al., 2020; Van Cutsem et al., 2020; Avallone et al., 2021; Kanemitsu et al., 2021; Antoniotti et al., 2022; Modest et al., 2022; Rossini et al., 2022; Ychou et al., 2022; Bond et al., 2023; Watanabe et al., 2023), this raised concerns about the blinding of participants and personnel, assessment of outcomes, and concealment of allocation. Furthermore, several RCTs were found to have potential bias because of insufficient availability of outcome data. The network’s results from the Egger test showed no publication bias, and the funnel plots can be found in Supplementary File S5.

### 3.3 Efficacy outcomes

#### 3.3.1 Primary analysis of overall survival for first-line treatments

For the analysis of OS and PFS, Fire-4.5 (a phase Ⅲ trial, also known as AIO KRK-0116) was not considered due to its focus on BRAF mutations (which only account for 5% of all mCRC patients), resulting in excessive heterogeneity (Stintzing et al., 2016).

**FIGURE 2:**
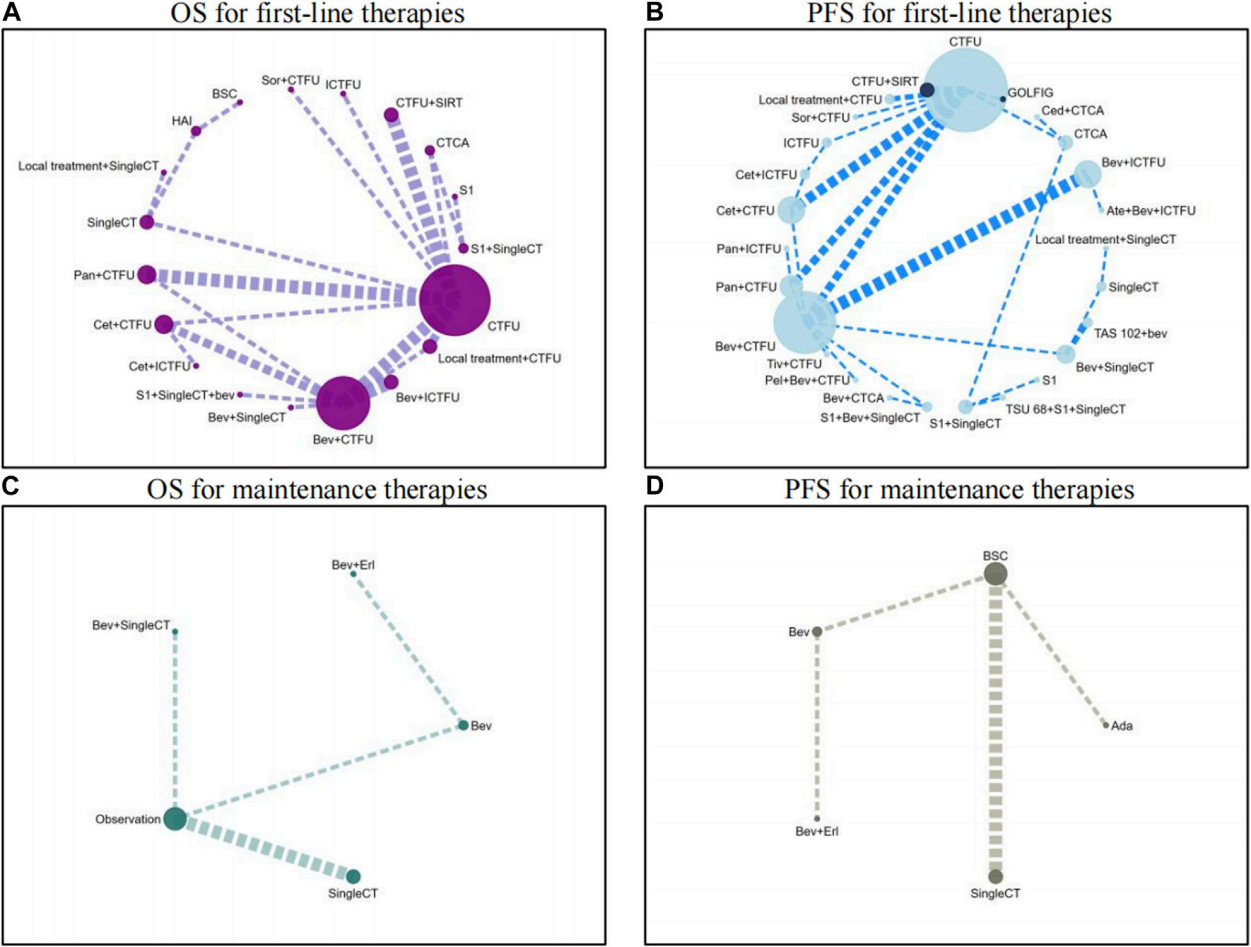
Network plots for first-line treatment and maintenance treatment in patients with unresectable colorectal liver metastases Abbreviation: Ada, Adavosertib; ATE, Atezolizumab; BEV, Bevacizumab; BSC, best supportive care; CED, Cediranib; CET, Cetuximab; CTCA, capecitabine-based combination chemotherapy; CTFU, fluorouracil-based combination chemotherapy; ERL, Erlotinib; HAI, hepatic artery infusion; ICTFU, intensified fluorouracil-based combination chemotherapy; PAN, panitumuma; SingleCT, single-drug chemotherapy; SIRT, Selective internal radiation therapy; SOR, sorafenib; TIV, tivozanib. **(A)** OS for first-line therapies; **(B)** PFS for first-line therapies; **(C)** OS for maintenance therapies; **(D)** PFS for maintenance therapies.

**FIGURE 3:**
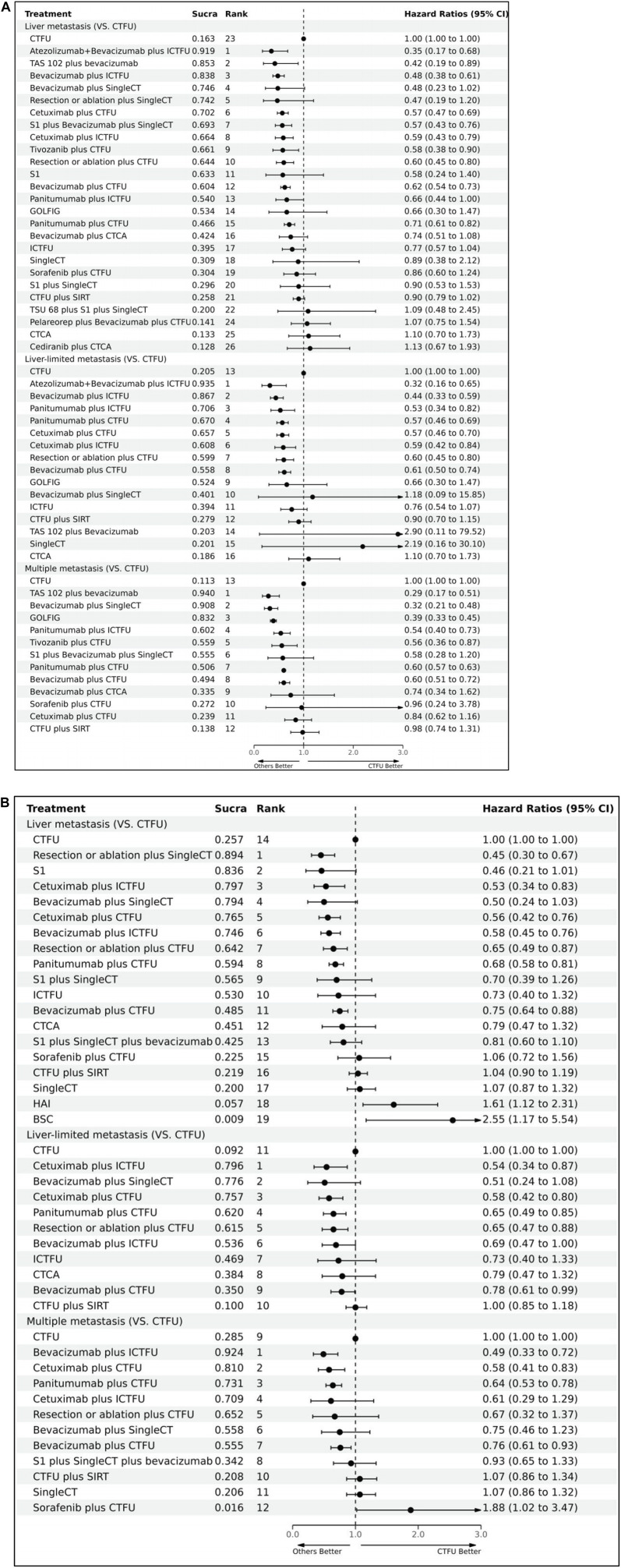
Forest plots illustrating the comparison results of primary and secondary endpoints Abbreviation: CTFU, fluorouracil-based combination chemotherapy; CTCA, capecitabine-based combination chemotherapy; ICTFU, intensified fluorouracil-based combination chemotherapy; SIRT, Selective internal radiation therapy; SingleCT, single-drug chemotherapy. **(A)** OS for first-line treatments; **(B)** PFS for first-line treatments.

For OS, network plot is presented in Figure 2A and Figure 2B. In the 9 intervention mechanisms, the top five rankings were, in order: local treatment + SingleCT (SUCRA, 0.938), TAR + SingleCT (SUCRA, 0.886), TAR + MultiCT (SUCRA, 0.732), Local treatment + MultiCT (SUCRA, 0.537), MultiCT (SUCRA, 0.411). Compared to MultiCT, the mechanisms with significant advantages were: local treatment + SingleCT (HR 0.45%, 95% CI 0.3–0.67), TAR + SingleCT (HR 0.49%, 95% CI 0.24–0.99), TAR + MultiCT (HR 0.74%, 95% CI 0.65–0.84). More details are shown in Table 1 and Supplementary File S6 (Supplementary Figure S1). In all 19 intervention schemes, the top five rankings were: R/A + SingleCT (SUCRA, 0.894), S1 (SUCRA, 0.836), Cetuximab + ICTFU (SUCRA, 0.797), Bevacizumab + SingleCT (SUCRA, 0.794), Cetuximab + CTFU (SUCRA, 0.765). Compared to CTFU, the ones with significant advantages, ranked from high to low, were as follows: R/A + SingleCT (HR 0.45%, 95% CI 0.3–0.67), Cetuximab + ICTFU (HR 0.53%, 95% CI 0.34–0.83), Cetuximab + CTFU (HR 0.56%, 95% CI 0.42–0.76), Bevacizumab + ICTFU (HR 0.58%, 95% CI 0.45–0.76), R/A + CTFU (HR 0.65%, 95% CI 0.49–0.87), Panitumumab + CTFU (HR 0.68%, 95% CI 0.58–0.81), and Bevacizumab + CTFU (HR 0.75%, 95% CI 0.64–0.88). Moreover, CTFU had a significant advantage compared to HAI (HR 1.61, 95% CI 1.12–2.31) and BSC (HR 2.55, 95% CI 1.17–5.54). Detailed results are provided in Figure 3A and Supplementary File S6 (Supplementary Table S3).

**TABLE 1:** Comparative results of overall survival and progression-free survival of first-line treatment mechanism in patients with unresectable colorectal liver metastases.

	Overall Survival											
Progression-free Survival	BSC	NA	NA	0.63 (0.32, 1.24)	**0.37 (0.17, 0.8)**	**0.18 (0.08, 0.4)**	**0.39 (0.18, 0.85)**	NA	NA	**0.42 (0.2, 0.88)**	**0.29 (0.13, 0.63)**	**0.19 (0.07, 0.55)**
	NA	ICI + MCT	NA	NA	NA	NA	NA	NA	NA	NA	NA	NA
	NA	1.29 (0.46, 3.6)	ICI + TAR + MCT	NA	NA	NA	NA	NA	NA	NA	NA	NA
	NA	NA	NA	LT	**0.58 (0.4, 0.86)**	**0.28 (0.18, 0.44)**	**0.62 (0.43, 0.9)**	NA	NA	**0.67 (0.49, 0.9)**	**0.46 (0.31, 0.68)**	**0.3 (0.14, 0.68)**
	NA	0.79 (0.35, 1.75)	0.61 (0.32, 1.18)	NA	LT + MCT	**0.48 (0.31, 0.73)**	1.07 (0.95, 1.2)	NA	NA	1.14 (0.9, 1.46)	**0.79 (0.67, 0.93)**	0.52 (0.25, 1.08)
	NA	1.03 (0.36, 2.96)	0.8 (0.31, 2.05)	NA	1.31 (0.65, 2.65)	LT + SCT	**2.22 (1.48, 3.34)**	NA	NA	**2.38 (1.68, 3.37)**	**1.64 (1.08, 2.52)**	1.09 (0.48, 2.48)
	NA	0.66 (0.3, 1.46)	**0.51 (0.27, 0.98)**	NA	**0.84 (0.75, 0.95)**	0.64 (0.32, 1.29)	MCT	NA	NA	1.07 (0.86, 1.32)	**0.74 (0.65, 0.84)**	**0.49 (0.24, 1)**
	NA	1.09 (0.48, 2.49)	0.85 (0.44, 1.65)	NA	**1.39 (1.09, 1.77)**	1.06 (0.54, 2.09)	**1.65 (1.34, 2.04)**	MTAR	NA	NA	NA	NA
	NA	0.54 (0.23, 1.29)	**0.42 (0.2, 0.86)**	NA	**0.69 (0.48, 0.99)**	0.53 (0.25, 1.13)	0.82 (0.58, 1.15)	**0.49 (0.34, 0.72)**	RNA + TAR + MCT	NA	NA	NA
	NA	0.55 (0.2, 1.48)	0.42 (0.18, 1.02)	NA	0.69 (0.37, 1.29)	**0.53 (0.38, 0.75)**	0.83 (0.45, 1.52)	**0.5 (0.28, 0.9)**	1.01 (0.51, 2)	SCT	**0.69 (0.54, 0.88)**	**0.46 (0.22, 0.96)**
	NA	0.93 (0.42, 2.07)	0.72 (0.38, 1.37)	NA	**1.18 (1.01, 1.38)**	0.9 (0.45, 1.8)	**1.41 (1.27, 1.55)**	0.85 (0.71, 1.02)	**1.72 (1.24, 2.39)**	1.7 (0.93, 3.1)	TAR + MCT	0.66 (0.33, 1.33)
	NA	1.01 (0.41, 2.48)	0.78 (0.36, 1.69)	NA	1.28 (0.83, 2)	0.98 (0.57, 1.71)	**1.53 (1, 2.34)**	0.92 (0.62, 1.38)	**1.87 (1.1, 3.18)**	**1.85 (1.2, 2.86)**	1.09 (0.72, 1.65)	TAR + SCT

Abbreviation: BSC, best supportive care; ICI, immune checkpoint inhibitor; LT, local treatment; MCT, multi-drug chemotherapy; MTAR, multi-targeted therapy; SCT, single-drug chemotherapy; TAR, targeted therapy.

Note: This table presents the comparative results for overall survival (right section) and progression-free survival (left section) among various first-line treatment mechanisms in patients with unresectable colorectal liver metastases. Each cell contains a comparison between two treatments, expressed as a ratio (95% confidence interval). Ratios in the lower-left cells (below the diagonal) represent the outcome of the treatment in the row compared to the treatment in the column; Ratios in the upper-right cells (above the diagonal) represent the outcome of the treatment in the column compared to the treatment in the row. NA, indicates that data is not available for a particular comparison. Bold values denote statistically significant results (*p* < 0.05). The interpretation method described above applies consistently to Tables 2–4.

#### 3.3.2 Primary analysis of progression-free survival for first-line treatments

For PFS, network plots are shown in Figures 2C,D. The top five ranked among the 11 intervention mechanisms were: ICI + TAR + MultiCT (SUCRA, 0.834), MultiTAR (SUCRA, 0.784), TAR + SingleCT (SUCRA, 0.675), Local treatment + SingleCT (SUCRA, 0.669), and ICI + MultiCT (SUCRA, 0.625). Compared to MultiCT, the mechanisms with significant advantages were: ICI + TAR + MultiCT (HR 0.51%, 95% CI 0.27–0.98), MultiTAR (HR 0.6%, 95% CI 0.49–0.75), TAR + SingleCT (HR 0.65 95% CI 0.43–1), TAR + MultiCT (HR 0.71%, 95% CI 0.64–79) and local treatment + MultiCT (HR 0.84 95% CI 0.75–0.95). Among all 26 intervention plans, the top five rankings were respectively Atezolizumab + Bevacizumab + ICTFU (SUCRA, 0.919), TAS-102+bevacizumab (SUCRA, 0.853), Bevacizumab + ICTFU (SUCRA, 0.838), Bevacizumab + SingleCT (SUCRA, 0.746), R/A + SingleCT (SUCRA, 0.742). Compared to CTFU, the scheme with significant advantages were: Atezolizumab + Bevacizumab + ICTFU (HR 0.35%, 95% CI 0.17–0.68), TAS-102+bevacizumab (HR 0.42%, 95% CI 0.19–0.89), Bevacizumab + ICTFU (HR 0.48, 95% CI 0.38–0.61), Cetuximab + CTFU (HR 0.57, 95% CI 0.47–0.69), S1+Bevacizumab + SingleCT (HR 0.57%, 95% CI 0.43–0.76), Cetuximab + ICTFU (HR 0.59%, 95% CI 0.43–0.79), Tivozanib + CTFU (HR 0.58%, 95% CI 0.38–0.9), R/A + CTFU (HR 0.6%, 95% CI 0.45–0.8), Bevacizumab + CTFU (HR 0.62%, 95% CI 0.54–0.73), and Panitumumab + CTFU (HR 0.71%, 95% CI 0.61–0.82). Other schemes, such as CTCA, showed no significant differences compared to CTFU. More details are shown in Table 1 and Figure 3B and Supplementary File S6 (Supplementary Table S3).

#### 3.3.3 Primary analysis of maintenance treatments

Network plots are provided in Figures 2C,D. Seven intervention strategies were included for comparison. For OS, compared to BSC, the significant improvements were: Bevacizumab + SingleCT (HR 0.71%, 95% CI 0.56–0.89) and SingleCT (HR 0.95%, 95% CI 0.91–0.98). Bevacizumab or Bevacizumab + Erlotinib did not show significant superiority when compared to BSC; In terms of PFS, Adavosertib (HR 0.35%, 95% CI 0.17–0.72) and SingleCT (HR 0.73%, 95% CI 0.65–0.81) had a significant advantage over BSC; Similarly, Bevacizumab or Bevacizumab + Erlotinib did not show significant differences compared to BSC. For more details, see Table 2. Due to the lack of safety data for CRLM patients, a quantitative comparison could not be made. However, the safety results of the overall patients indicated that monotherapy was a safer choice for maintenance treatment. See more in Supplementary File S9.

**TABLE 2:** Comparative results of overall survival and progression-free survival of maintenance treatment in patients with unresectable colorectal liver metastases.

Progression-free Survival	Overall Survival					
	ADA	NA	NA	NA	NA	NA
	**0.41 (0.19, 0.87)**	BEV	1.05 (0.81, 1.36)	0.71 (0.13, 3.88)	1 (0.19, 5.4)	0.95 (0.18, 5.1)
	0.46 (0.2, 1.04)	1.13 (0.84, 1.52)	BEV + ERL	0.68 (0.12, 3.79)	0.95 (0.17, 5.25)	0.9 (0.16, 4.97)
	NA	NA	NA	BEV + SCT	**1.41 (1.12, 1.77)**	**1.33 (1.06, 1.68)**
	0.35 (0.17, 0.72)	0.86 (0.68, 1.08)	0.76 (0.52, 1.1)	NA	BSC	0.95 (0.91, 0.98)
	**0.48 (0.23, 1)**	1.18 (0.91, 1.53)	1.04 (0.7, 1.54)	NA	**1.37 (1.23, 1.54)**	SCT

Abbreviation: ADA, adavosertib; BEV, bevacizumab; BSC, best supportive care; ERL, erlotinib; SCT, single-drug chemotherapy.

#### 3.3.4 Primary analysis of safety, ORR, and R0 resection rate for first-line treatments

Network plots are presented in Supplementary File S6, Supplementary Figure S2. In terms of safety, for first-line treatments, MultiCT was the safest choice (SUCRA, 0.998), followed by local treatment + MultiCT (SUCRA, 0.795), TAR + MultiCT was the worst (SUCRA, 0.007). MultiCT showed significant advantages in terms of safety compared to other targeted combination therapies and local combination therapies. CTFU is the safest choice, and the combined mechanism therapy increased the incidence of SAEs compared to chemotherapy. Specifically, the treatment plans with significant differences compared to CTFU were: Bevacizumab + ICTFU (OR 4.5 95% CI 2.29–8.92), Panitumumab + CTFU (OR 4.07 95% CI 2.3–7.29), Cetuximab + CTFU (OR 3.27 95% CI 1.35–7.88), Bevacizumab + CTFU (OR 1.99 95% CI 1.23–3.24), and CTFU + SIRT (OR 1.43 95% CI 1.1–1.86). For ORR, compared to CTFU, combined mechanism therapies have significant advantages, ranked from high to low were: Panitumumab plus CTFU (OR 6.98, 95% CI 3.3–15.01), Cetuximab + ICTFU (OR 6.5%, 95% CI 2.97–12.5), Panitumumab + ICTFU (OR 5.26%, 95% CI 1.72–16.16), Bevacizumab + ICTFU (OR 4.71%, 95% CI 2.49–8.92), Cetuximab + CTFU (OR 3.99%, 95% CI 2.3–7.02), Bevacizumab + CTFU (OR 1.93%, 95% CI 1.21–3.08), and CTFU + SIRT (OR 1.55%, 95% CI 1.21–2). There was no significant difference between CTFU, CTCA, and ICTFU. In terms of the R0 resection rate, the significant advantages in order compared to CTFU were: Bevacizumab + ICTFU (OR 15.81%, 95% CI 5.87–45.53), Bevacizumab + CTFU (OR 5.12%, 95% CI 2.53–11.13), and Cetuximab + CTFU (OR 4.09%, 95% CI 1.92–9.16). Cetuximab + ICTFU was better than BSC (OR 2.69, 95% CI 0.69–11.61), but the difference was not statistically significant. CTFU, ICTFU, and CTCA had almost no difference. More details are provided in Tables 3, 4 and Supplementary File S6, Supplementary Figure S3.

**TABLE 3:** Comparative results of safety of first-line treatment in patients with unresectable colorectal liver metastases.

BEV + CTFU					
**0.44 (0.27, 0.71)**	BEV + ICTFU				
0.61 (0.29, 1.27)	1.38 (0.57, 3.33)	CET + CTFU			
**1.99 (1.23, 3.24)**	**4.5 (2.29, 8.92)**	**3.27 (1.35, 7.88)**	CTFU		
1.39 (0.81, 2.42)	**3.15 (1.53, 6.56)**	2.28 (0.91, 5.72)	**0.7 (0.54, 0.91)**	CTFU + SIRT	
**0.49 (0.3, 0.79)**	1.11 (0.56, 2.18)	0.8 (0.33, 1.94)	**0.25 (0.14, 0.44)**	**0.35 (0.19, 0.66)**	PAN + CTFU

Abbreviation: BEV, bevacizumab; CET, cetuximab; CTFU, fluorouracil-based combination chemotherapy; ICTFU, intensified fluorouracil-based combination chemotherapy; PAN, panitumuma; SIRT, selective internal radiation therapy.

**TABLE 4:** Comparative results of R0 liver resection rate and objective response rate of first-line treatment in patients with unresectable colorectal liver metastases.

ORR	R0									
	BEV + CTFU	**3.06 (1.55, 6.38)**	0.8 (0.41, 1.54)	0.52 (0.11, 2.66)	**0.2 (0.06, 0.67)**	**0.2 (0.09, 0.39)**	NA	**0.23 (0.07, 0.81)**	NA	NA
	**0.41 (0.27, 0.63)**	BEV + ICTFU	**0.26 (0.1, 0.68)**	**0.17 (0.03, 0.99)**	**0.06 (0.01, 0.26)**	**0.06 (0.02, 0.17)**	NA	**0.08 (0.02, 0.32)**	NA	NA
	**0.48 (0.26, 0.89)**	1.18 (0.55, 2.49)	CET + CTFU	0.66 (0.14, 3.45)	**0.25 (0.07, 0.87)**	**0.24 (0.11, 0.52)**	NA	0.29 (0.08, 1.05)	NA	NA
	**0.32 (0.15, 0.68)**	0.78 (0.32, 1.87)	0.66 (0.39, 1.1)	CET + ICTFU	0.37 (0.06, 2.03)	0.37 (0.09, 1.44)	NA	0.45 (0.16, 1.16)	NA	NA
	1.85 (0.7, 4.93)	**4.54 (1.56, 13.13)**	**3.84 (1.39, 10.69)**	**5.84 (1.91, 17.91)**	CTCA	0.98 (0.36, 2.7)	NA	1.18 (0.3, 4.97)	NA	NA
	**1.93 (1.21, 3.08)**	**4.71 (2.49, 8.92)**	**3.99 (2.3, 7.02)**	**6.05 (2.97, 12.5)**	1.04 (0.44, 2.44)	CTFU	NA	1.2 (0.47, 3.37)	NA	NA
	1.24 (0.73, 2.12)	**3.03 (1.53, 6.03)**	**2.56 (1.4, 4.77)**	**3.89 (1.84, 8.4)**	0.67 (0.27, 1.63)	**0.64 (0.5, 0.83)**	CTFU + SIRT	NA	NA	NA
	1.76 (0.75, 4.28)	**4.31 (1.66, 11.57)**	**3.65 (1.58, 8.7)**	**5.53 (2.31, 13.86)**	0.95 (0.3, 3.02)	0.92 (0.44, 1.98)	1.42 (0.65, 3.21)	ICTFU	NA	NA
	**0.28 (0.15, 0.5)**	0.67 (0.32, 1.4)	0.57 (0.24, 1.33)	0.87 (0.33, 2.29)	**0.15 (0.05, 0.46)**	**0.14 (0.07, 0.3)**	**0.22 (0.1, 0.49)**	**0.16 (0.05, 0.44)**	PAN + CTFU	NA
	0.37 (0.13, 1.02)	0.9 (0.3, 2.72)	0.76 (0.23, 2.5)	1.15 (0.32, 4.19)	**0.2 (0.05, 0.82)**	**0.19 (0.06, 0.58)**	**0.3 (0.09, 0.93)**	**0.21 (0.05, 0.8)**	1.33 (0.58, 3.09)	PAN + ICTFU

Abbreviation: BEV, bevacizumab; CET, cetuximab; CTFU, fluorouracil-based combination chemotherapy; CTCA, capecitabine-based combination chemotherapy; ICTFU, intensified fluorouracil-based combination chemotherapy; PAN, panitumuma; SIRT, Selective internal radiation therapy.

#### 3.3.5 Subgroup analysis

For patients with liver-limited metastatic, the top five ranked regimens in terms of PFS were Atezolizumab + Bevacizumab + ICTFU (SUCRA 0.935), Bevacizumab + ICTFU (SUCRA 0.867), Panitumumab + ICTFU (SUCRA 0.706), Panitumumab + CTFU (SUCRA 0.67), and Cetuximab + CTFU (SUCRA 0.657). Compared to CTFU, the schemes that possessed significant advantages were: Atezolizumab + Bevacizumab + ICTFU (HR 0.32%, 95% CI 0.16–0.65), Bevacizumab + ICTFU (HR 0.44%, 95% CI 0.33–0.59), Panitumumab + ICTFU (HR 0.53%, 95% CI 0.34–0.82), Panitumumab + CTFU (HR 0.57%, 95% CI 0.46–0.69), Cetuximab + CTFU (HR 0.57%, 95% CI 0.46–0.7), Cetuximab + ICTFU (HR 0.59%, 95% CI 0.42–0.84), R/A + CTFU (HR 0.6%, 95% CI 0.45–0.8), and Bevacizumab + CTFU (HR 0.61%, 95% 0.5–0.74). In terms of OS, the top five ranked options were: Cetuximab + ICTFU (SUCRA 0.796), Bevacizumab + SingleCT (SUCRA 0.776), Cetuximab + CTFU (SUCRA 0.757), Panitumumab + CTFU (SUCRA 0.62), and R/A + CTFU (SUCRA 0.615). Compared to CTFU, the plans with significant advantages were: Cetuximab + ICTFU (HR 0.54, 95% CI 0.34–0.87), Cetuximab + CTFU (HR 0.58%, 95% CI 0.42–0.8), Panitumumab + CTFU (HR 0.65%, 95% CI 0.45–0.85), R/A + CTFU (HR 0.65%, 95% CI 0.47–0.88), Bevacizumab + ICTFU (HR 0.69%, 95% 0.47–1.00), and Bevacizumab + CTFU (HR 0.78%, 95% 0.61–0.99).

For patients with multiple-site metastases, the top five treatments in terms of PFS were: TAS-102+bevacizumab (SUCRA 0.94), Bevacizumab + SingleCT (SUCRA 0.908), GOLFIG (SUCRA 0.832), Panitumumab + ICTFU (SUCRA 0.602), and Tivozanib + CTFU (SUCRA 0.559). Compared to CTFU, the solutions with significant advantages were as follows: TAS-102+bevacizumab (HR 0.29%, 95% CI 0.17–0.51), Bevacizumab + SingleCT (HR 0.32%, 95% CI 0.21–0.48), GOLFIG (HR 0.39%, 95% CI 0.33–0.45), Panitumumab + ICTFU (HR 0.54%, 95% CI 0.4–0.73), Tivozanib + CTFU (HR 0.56%, 95% CI 0.36–0.87), Panitumumab + CTFU (HR 0.6%, 95% CI 0.57–0.63), Bevacizumab + CTFU (HR 0.6%, 95% CI 0.51–0.72). In terms of OS, the top five ranked solutions were: Bevacizumab + ICTFU (SUCRA 0.924), Cetuximab + CTFU (SUCRA 0.81), Panitumumab + CTFU (SUCRA 0.731), Cetuximab + ICTFU (SUCRA 0.709), and R/A + CTFU (SUCRA 0.652). Compared to CTFU, the treatments with significant advantages were as follows: Bevacizumab + ICTFU (HR 0.49%, 95% CI 0.33–0.72), Cetuximab + CTFU (HR 0.58%, 95% CI 0.41–0.83), Panitumumab + CTFU (HR 0.64%, 95% CI 0.53–0.78), and Bevacizumab + CTFU (HR 0.76%, 95% 0.61–0.93). Detailed results are presented in Figure 3 and Supplementary File S7, Supplementary Tables S4, S5.

#### 3.3.6 Heterogeneity and inconsistency assessment

Most of the comparisons showed minimal or low heterogeneity, as observed in the results of the heterogeneity test summarized in Supplementary File S10, 11. Nevertheless, comparisons of demonstrated moderate to high heterogeneity were as follows:

A. Bevacizumab + CTFU VS Bevacizumab + ICTFU (54.2%), Panitumumab + CTFU (78.3%), or CTFU (65.8%) for PFS in liver metastasis;
B. Bevacizumab + CTFU (54.3%) or Panitumumab + CTFU (56.9%) VS CTFU and Monotherapy VS NAT (66% for the long-term and 68% for the short-term) for PFS in multiple organ metastasis;
C. Bevacizumab + CTFU VS Bevacizumab + ICTFU (65%), Panitumumab + CTFU (60.5%), or CTFU (70.4%) for liver-limited PFS;
D. SingleCT VS BSC for Maintenance Treatment (OS, 98.4%; PFS, 98.6%).
E. In the network of mechanism comparison, MultiCT VS Local treatment + MultiCT (50.4%) and Target + MultiCT VS MultiCT (54.1%).

After conducting pairwise meta-analyses, good consistency was observed between direct and indirect evidence. During the analysis of node splitting, we found no significant discrepancies between direct and indirect estimates, as all *p* values in the inconsistency test exceeded 0.05. The trace plots indicated a favorable convergence of iterations (Supplementary File S12).

#### 3.3.7 Sensitivity analysis

Firstly, only RCTs that did not differentiate patients based on the level of target gene mutations were included, and the results were generally consistent with the baseline analysis: in terms of PFS, with CTFU as the reference, Atezolizumab + Bevacizumab + ICTFU (HR 0.34%, 95% 0.16–0.7) still ranked first, followed by Bevacizumab + ICTFU (HR 0.47%, 95% 0.33–0.66), and TAS-102+bevacizumab (HR 0.43%, 95% 0.19–0.94). Other treatments showed significant advantages in comparison to CTFU were S1+Bevacizumab + SingleCT (HR 0.59%, 95% 0.41–0.84), R/A + CTFU (HR 0.6%, 95% 0.45–0.8), Tivozanib + CTFU (HR 0.6%, 95% 0.37–0.97), Cetuximab + CTFU (HR 0.61, 95% 0.46–0.81), Cetuximab + ICTFU (HR 0.64%, 95% 0.43–0.95), and Bevacizumab + CTFU (HR 0.64%, 95% 0.49–0.84). OS results were consistent with the results of the base-case analysis, and treatments with significant advantages compared to CTFU were: R/A + SingleCT (HR 0.6%, 95% 0.37–0.97), Bevacizumab + SingleCT (HR 0.44%, 95% 0.21–0.94), Bevacizumab + ICTFU (HR 0.52%, 95% 0.37–0.73), R/A + CTFU (HR 0.54%, 95% 0.48–0.85), and Bevacizumab + CTFU (HR 0.67%, 95% 0.51–0.88).

Secondly, focus on patients with wild-type RAS/RAF. In terms of PFS, compared to CTFU, it was ranked from high to low as follows: Cetuximab + CTFU (HR 0.53%, 95% 0.39–0.72), Bevacizumab + ICTFU (HR 0.71%, 95% 0.45–1.14), Panitumumab + ICTFU (HR 0.74%, 95% 0.49–1.13), Panitumumab + CTFU (HR 0.79%, 95% 0.69–0.95), and Bevacizumab + CTFU (HR 0.79%, 95% 0.61–1.03). For OS, compared to CTFU, the best choices were still Cetuximab + CTFU (HR 0.61%, 95% 0.43–0.85), followed by Panitumumab + CTFU (HR 0.74%, 95% 0.61–0.91) and Bevacizumab + CTFU (HR 0.88%, 95% 0.68–1.14). More details are available in Supplementary File S8, Supplementary Tables S6, S7.

## 4 Discussion

### 4.1 Main findings

This study is the first to systematically evaluate the efficacy and safety of different treatment options for patients with CRLM. The key findings are summarized as follows:

1. For CRLM patients, the optimal treatment options were local treatment + chemotherapy and TAR + chemotherapy. In terms of overall survival (OS), the best choices were R/A+ SingleCT or CTFU, Cetuximab + ICTFU or CTFU, Bevacizumab + ICTFU or CTFU, and Panitumumab + CTFU. For progression-free survival (PFS), the top options were Immune + TAR + chemotherapy, MultiTAR, local treatment + chemotherapy, and TAR + chemotherapy. For patients with liver-limited metastasis, Cetuximab, Bevacizumab, and Panitumumab + chemotherapy were the best choices for both OS and PFS. For those with multiple metastatic sites, Bevacizumab + ICTFU, Cetuximab or Panitumumab + CTFU were the best for OS, while TAS-102 + Bevacizumab, Bevacizumab + SingleCT, and GOLFIG were optimal for PFS.
2. For maintenance treatment, Bevacizumab + SingleCT was the best choice for OS. For PFS, Adavosertib and SingleCT showed significant advantages compared to BSC.
3. For first-line treatments, combination therapy caused more SAEs compared to CTFU. Bevacizumab + chemotherapy was the safest among targeted combination therapies. For ORR, Panitumumab or Cetuximab + CTFU or ICTFU, and Bevacizumab + ICTFU showed significant advantages over CTFU. Bevacizumab + ICTFU had the best R0 resection rate, followed by Bevacizumab or Cetuximab + CTFU.
4. For RAS/RAF wild-type patients, Cetuximab + CTFU was the best choice for both PFS and OS.

In the base-case analysis, heterogeneity was observed in some networks, such as the PFS of the overall population, likely due to lack of limitation on target mutation levels. Sensitivity analysis confirmed that controlling for target expression levels reduced heterogeneity across all networks.

TAR + chemotherapy can produce a higher remission rate and improve resectability. EGFR or VEGF inhibitors combined with chemotherapy are the best choices for patients with unresectable CRLM. EGFR inhibitors, such as Cetuximab and Panitumumab, are associated with higher tumor response and expedited symptom relief. Anti-EGFR therapy may induce tumor-specific adaptive immune responses and immunogenic cell apoptosis. VEGF inhibitors, such as Bevacizumab and Aflibercept, normalize tumor vasculature, increasing tumor blood supply (Xie et al., 2020). Combining chemotherapy with targeted therapy can enhance patient survival, but it also increases SAEs, making it challenging to find a solution that is both effective and safe. Surprisingly, adding Cetuximab to ICTFU did not worsen safety compared to CTFU, and Bevacizumab + ICTFU is also acceptable in terms of safety. Thus, Bevacizumab + ICTFU and Cetuximab + ICTFU are optimal for multiple metastasis sites and liver-limited metastasis, respectively. For the WT population, Cetuximab + CTFU is the best choice. New therapies like ICI + TAR and MultiTAR showed great PFS performance but had poor safety profiles, and more OS data are needed to confirm their efficacy. More RCTs are needed to identify the applicable population for these new mechanisms in precision treatment for CRC.

Despite using fewer drugs, monotherapy has been relatively effective in maintaining treatment efficacy for both PFS and OS. Bevacizumab combined with SingleCT is the most effective for OS, but its safety has been poor (Simkens et al., 2015). On the other hand, Adavosertib has demonstrated the best PFS, but more data is needed to establish its safety profile. Overall, monotherapy may still be the ideal choice at present.

Right- and left-sided colorectal tumors exhibit distinct epidemiological, clinicopathological characteristics, gene expression profiles, genetic alterations, and prognoses. Thus, efficacy of EGFR inhibitors is significantly influenced by the primary tumor site. Moretto et al. (2016) demonstrated that anti-EGFR therapies are less effective in right-sided tumors compared to left-sided ones. Takayuki’s meta-analysis (Yoshino et al., 2024) further confirmed the superior efficacy of EGFR inhibitors in patients with left-sided primary tumors. Due to data limitations, our study could not differentiate the primary tumor site in patients with liver metastases. Future research should address this aspect more thoroughly.

Recent trials have explored the use of anti-EGFR monoclonal antibodies in maintenance therapy. Filippo et al.'s phase II trial indicated that using panitumumab alone was less effective for PFS compared to a combination of panitumumab with fluorouracil-leucovorin (Pietrantonio et al., 2019). Similarly, the ERMES study concluded that cetuximab alone was not as effective for maintenance following FOLFIRI/cetuximab induction and thus is not recommended (Pinto et al., 2024). Due to the focus on liver metastasis in our study and limited available data, we could not evaluate additional maintenance therapies, including anti-EGFR monoclonal antibodies.

The advantage of this research is clear. It is the first study to systematically compare the efficacy and safety of all first-line and maintenance treatment regimens for CRLM patients, providing significant reference value for clinical practice and guidelines. Unlike previous studies, this study meticulously analyzed the type and mechanism of chemotherapy to minimize heterogeneity. The low heterogeneity of the study population enhances the reliability of the conclusions. Sensitivity analysis and in-depth examination of heterogeneity sources confirmed the robustness of the basic analysis. Multiple subgroup analyses, including distinctions between liver-limited and multiple-site metastases and analyses of wild-type patients, support precision treatment for CRC. Our innovative conclusions offer a new direction for future clinical research and provide substantial evidence for clinical decision-making.

Due to the availability of data, this study has some limitations. First, we cannot analyze the RAS/KRAS or RAF mutation population. Second, limited by a lack of individual data, the majority of studies only reported HR. Therefore, we utilized the time-invariant HR methods for indirect comparison, as opposed to using other risk variable models. Third, in order to form more comparisons, we consider patients with multiple-site metastases to have liver metastasis, even though this proportion exceeds 90%, it also introduces some uncertainty. Fourth, the relative efficacy between a certain number of schemes is obtained through indirect comparison, and more direct evidence from RCTs is needed to validate our findings. Fifth, for the results related to immunotherapy, a more cautious interpretation is required, as the evidence is based on a small subset of patients. Additionally, more clinical evidence is needed to validate the conclusions of this study.

## 5 Conclusion

For unresectable CRLM patients without prior systemic therapy, local treatment or targeted therapy plus chemotherapy are optimal. R/A combined with SingleCT or CTFU performed best for OS, while Atezolizumab + Bevacizumab + ICTFU was the best for PFS. For maintenance treatment, Bevacizumab + SingleCT was optimal for OS, and Adavosertib for PFS. Cetuximab + CTFU was the best choice for RAS/RAF wild-type patients. Combination therapy resulted in more SAEs compared to standard chemotherapy, with Bevacizumab + chemotherapy being the safest among targeted combinations. Our findings offer additional supporting evidence for current guideline recommendations.

## Figures and Tables

**Table:** 

**CRLM**	colorectal liver metastases
**RCTs**	randomized controlled trials
**OS**	overall survival
**PFS**	progression-free survival
**ORR**	objective response rate
**SAE**	adverse effects graded as 3 or above/severe adverse effects
**HRs**	Hazards Ratios
**CI**	confidence intervals
**ORs**	odds ratios
**SingleCT**	single-drug chemotherapy
**TAR**	Targeted therapy
**MultiCT**	multi-drug chemotherapy
**R/A**	Resection or ablation
**ICTFU**	intensified fluorouracil-based combination chemotherapy
**MultiTAR**	multi-targeted therapy
**CTFU**	fluorouracil-based combination chemotherapy
**CTCA**	capecitabine-based combination
**CRC**	colorectal cancer
**MRI**	magnetic resonance imaging
**TACE**	transarterial chemoembolization
**AEs**	adverse effects
**EGFR**	epithelial growth factor receptor
**ICB**	immune checkpoint blockade
**MSI**	microsatellite instability
**MMR**	mismatch repair
**PRISMA**	Preferred Reporting Items for Systematic Reviews and Meta-Analyses
**IPD**	individual patient data
**ROB**	risk of bias
**NMA**	network meta-analysis
**SUCRA**	surface area under the cumulative ranking
**mCRC**	metastasis CRC
**ICI**	immune checkpoint inhibitor
**HAI**	hepatic artery infusion
**SIRT**	selective internal radiation therapy
**TSU-68**	Orantinib
**TAS-102**	trifluridine/tipiracil
**S1**	combined chemotherapy of tegafur, gimeracil, and oteracil

## Data Availability

The raw data supporting the conclusions of this article will be made available by the authors, without undue reservation.
