# Supplementary material for: A network meta-analysis of efficacy and safety for first-line and maintenance therapies in patients with unresectable colorectal liver metastases
Source: Front Pharmacol. 2024 Jul 26;15:1374136. doi: 10.3389/fphar.2024.1374136 (PMC11310042; doi:10.3389/fphar.2024.1374136)
Supplement: Supplementary file 1 [file DataSheet1.docx]

**Supplement File**

[Supplementary File 1. Checklist of the PRISMA extension for Systematic Review and Network Meta-Analysis 2](#_Toc22875)

[Supplementary File 2. Search terms for meta-analysis 6](#_Toc17949)

[Supplementary File 3. Characteristics of the included Trials and definitions of primary chemotherapy and targeted therapies 7](#_Toc6857)

[Supplementary File 4. Cochrane risk of the bias assessment tool 11](#_Toc9117)

[Supplementary File 5. Funnel plots to show publication bias 12](#_Toc11653)

[Supplementary File 6. Additional results of primary analyses for first-line treatments 13](#_Toc14290)

[Supplementary File 7. Additional results of subgroup analyses for first-line treatments 18](#_Toc8523)

[Supplementary File 8. Additional results of sensitive analyses 21](#_Toc2672)

[Supplementary File 9. Additional results of maintenance treatment 24](#_Toc25154)

[Supplementary File 10. Heterogeneity assessment results 25](#_Toc21821)

[Supplementary File 11. Forest plots depicting results of head-to-head comparisons and node-splitting analysis of inconsistency 26](#_Toc12390)

[Supplementary File 12. Brooks-Gelman-Rubin diagnostic 54](#_Toc22607)

# Supplementary File 1. Checklist of the PRISMA extension for Systematic Review and Network Meta-Analysis

| **Section/Topic** | **Item #** | **Checklist Item** | **Reported on Page #** |
| --- | --- | --- | --- |
| **TITLE** |  |  |  |
| Title | 1 | Identify the report as a systematic review *incorporating a network meta-analysis (or related form of meta-analysis).* | ***1*** |
|  |  |  |  |
| **ABSTRACT** |  |  |  |
| Structured summary | 2 | Provide a structured summary including, as applicable:  **Background:** main objectives  **Methods:** data sources; study eligibility criteria, participants, and interventions; study appraisal; and *synthesis methods, such as network meta-analysis.*  **Results:** number of studies and participants identified; summary estimates with corresponding confidence/credible intervals; *treatment rankings may also be discussed. Authors may choose to summarize pairwise comparisons against a chosen treatment included in their analyses for brevity.*  **Discussion/Conclusions:** limitations; conclusions and implications of findings.  **Other:** primary source of funding; systematic review registration number with registry name. | 2 |
|  |  |  |  |
| **INTRODUCTION** |  |  |  |
| Rationale | 3 | Describe the rationale for the review in the context of what is already known*, including mention of why a network meta-analysis has been conducted.* | ***3*** |
| Objectives | 4 | Provide an explicit statement of questions being addressed, with reference to participants, interventions, comparisons, outcomes, and study design (PICOS). | 3-4 |
|  |  |  |  |
| **METHODS** |  |  |  |
| Protocol and registration | 5 | Indicate whether a review protocol exists and if and where it can be accessed (e.g., Web address); and, if available, provide registration information, including registration number. | 5 |
| Eligibility criteria | 6 | Specify study characteristics (e.g., PICOS, length of follow-up) and report characteristics (e.g., years considered, language, publication status) used as criteria for eligibility, giving rationale. *Clearly describe eligible treatments included in the treatment network, and note whether any have been clustered or merged into the same node (with justification).* | ***5*** |
| Information sources | 7 | Describe all information sources (e.g., databases with dates of coverage, contact with study authors to identify additional studies) in the search and date last searched. | 5 |
| Search | 8 | Present full electronic search strategy for at least one database, including any limits used, such that it could be repeated. | 5 |
| Study selection | 9 | State the process for selecting studies (i.e., screening, eligibility, included in systematic review, and, if applicable, included in the meta-analysis). | 5 |
| Data collection process | 10 | Describe method of data extraction from reports (e.g., piloted forms, independently, in duplicate) and any processes for obtaining and confirming data from investigators. | 5 |
| Data items | 11 | List and define all variables for which data were sought (e.g., PICOS, funding sources) and any assumptions and simplifications made. | 5 |
| **Geometry of the network** | **S1** | Describe methods used to explore the geometry of the treatment network under study and potential biases related to it. This should include how the evidence base has been graphically summarized for presentation, and what characteristics were compiled and used to describe the evidence base to readers. | ***5-6*** |
| Risk of bias within individual studies | 12 | Describe methods used for assessing risk of bias of individual studies (including specification of whether this was done at the study or outcome level), and how this information is to be used in any data synthesis. | 5-6 |
| Summary measures | 13 | State the principal summary measures (e.g., risk ratio, difference in means). *Also describe the use of additional summary measures assessed, such as treatment rankings and surface under the cumulative ranking curve (SUCRA) values, as well as modified approaches used to present summary findings from meta-analyses.* | 5-6 |
| Planned methods of analysis | 14 | Describe the methods of handling data and combining results of studies for each network meta-analysis. This should include, but not be limited to:   - *Handling of multi-arm trials;* - *Selection of variance structure;* - *Selection of prior distributions in Bayesian analyses; and* - *Assessment of model fit.* | 6 |
| **Assessment of Inconsistency** | **S2** | Describe the statistical methods used to evaluate the agreement of direct and indirect evidence in the treatment network(s) studied. Describe efforts taken to address its presence when found. | 6 |
| Risk of bias across studies | 15 | Specify any assessment of risk of bias that may affect the cumulative evidence (e.g., publication bias, selective reporting within studies). | **5-6** |
| Additional analyses | 16 | Describe methods of additional analyses if done, indicating which were pre-specified. This may include, but not be limited to, the following:   - Sensitivity or subgroup analyses; - Meta-regression analyses; - *Alternative formulations of the treatment network; and* - *Use of alternative prior distributions for Bayesian analyses (if applicable).* | ***6*** |
|  |  |  |  |
| **RESULTS†** |  |  |  |
| Study selection | 17 | Give numbers of studies screened, assessed for eligibility, and included in the review, with reasons for exclusions at each stage, ideally with a flow diagram. | 6-7 |
| **Presentation of network structure** | **S3** | Provide a network graph of the included studies to enable visualization of the geometry of the treatment network. | ***7-9*** |
| **Summary of network geometry** | **S4** | Provide a brief overview of characteristics of the treatment network. This may include commentary on the abundance of trials and randomized patients for the different interventions and pairwise comparisons in the network, gaps of evidence in the treatment network, and potential biases reflected by the network structure. | ***7-9*** |
| Study characteristics | 18 | For each study, present characteristics for which data were extracted (e.g., study size, PICOS, follow-up period) and provide the citations. | 6-7 |
| Risk of bias within studies | 19 | Present data on risk of bias of each study and, if available, any outcome level assessment. | 7 |
| Results of individual studies | 20 | For all outcomes considered (benefits or harms), present, for each study: 1) simple summary data for each intervention group, and 2) effect estimates and confidence intervals. *Modified approaches may be needed to deal with information from larger networks.* | ***7-9*** |
| Synthesis of results | 21 | Present results of each meta-analysis done, including confidence/credible intervals. *In larger networks, authors may focus on comparisons versus a particular comparator (e.g. placebo or standard care), with full findings presented in an appendix. League tables and forest plots may be considered to summarize pairwise comparisons.* If additional summary measures were explored (such as treatment rankings), these should also be presented. | ***7-9*** |
| **Exploration for inconsistency** | **S5** | Describe results from investigations of inconsistency. This may include such information as measures of model fit to compare consistency and inconsistency models, *P* values from statistical tests, or summary of inconsistency estimates from different parts of the treatment network. | ***9-10*** |
| Risk of bias across studies | 22 | Present results of any assessment of risk of bias across studies for the evidence base being studied. | 7 |
| Results of additional analyses | 23 | Give results of additional analyses, if done (e.g., sensitivity or subgroup analyses, meta-regression analyses*, alternative network geometries studied, alternative choice of prior distributions for Bayesian analyses,* and so forth). | ***9-10*** |
|  |  |  |  |
| **DISCUSSION** |  |  |  |
| Summary of evidence | 24 | Summarize the main findings, including the strength of evidence for each main outcome; consider their relevance to key groups (e.g., healthcare providers, users, and policy-makers). | 10-11 |
| Limitations | 25 | Discuss limitations at study and outcome level (e.g., risk of bias), and at review level (e.g., incomplete retrieval of identified research, reporting bias). *Comment on the validity of the assumptions, such as transitivity and consistency. Comment on any concerns regarding network geometry (e.g., avoidance of certain comparisons).* | 12 |
| Conclusions | 26 | Provide a general interpretation of the results in the context of other evidence, and implications for future research. | 12 |
|  |  |  |  |
| **FUNDING** |  |  |  |
| Funding | 27 | Describe sources of funding for the systematic review and other support (e.g., supply of data); role of funders for the systematic review. This should also include information regarding whether funding has been received from manufacturers of treatments in the network and/or whether some of the authors are content experts with professional conflicts of interest that could affect use of treatments in the network. | ***1*** |

PICOS = population, intervention, comparators, outcomes, study design.

* Text in italics indicateS wording specific to reporting of network meta-analyses that has been added to guidance from the PRISMA statement.

† Authors may wish to plan for use of appendices to present all relevant information in full detail for items in this section.

*The Checklist of the PRISMA extension is formulated for RICE-META1st with the structure of PRISMA NMA Checklist of

Items to Include When Reporting a Systematic Review Involving a Network Meta-analysis in https://prismastatement.org/Extensions/NetworkMetaAnalysis

# Supplementary File 2. Search terms for meta-analysis

1. ("colorectal cancer"[Title] OR "colorectal liver metastasis"[Title] OR "colorectal liver-limited metastases"[Title])AND ("metastatic"[Title/Abstract] OR "advanced"[Title/Abstract]) AND "English"[Language] AND ("compare"[Title/Abstract] OR "comparison"[Title/Abstract] OR "comparative"[Title/Abstract] OR "comparing"[Title/Abstract] OR "versus"[Title/Abstract] OR "vs"[Title/Abstract]) AND ("randomized controlled trial"[Publication Type] OR "controlled clinical trial"[Publication Type] OR "Randomized"[Title/Abstract] OR "randomised"[Title/Abstract] OR "randomly"[Title/Abstract] OR "clinical"[Title/Abstract] OR "Trial"[Title/Abstract] OR "phase"[Title/Abstract])
2. (("colorectal liver metastases"[Title] OR "colorectal liver-limited metastases"[Title]) AND "English"[Language] OR"unresectable"[Title/Abstract] AND ("randomized controlled trial"[Publication Type] OR "controlled clinical trial"[Publication Type] OR "Randomized"[Title/Abstract] OR "randomised"[Title/Abstract] OR "randomly"[Title/Abstract] OR "Trial"[Title/Abstract] OR "phase"[Title/Abstract])) OR (("colorectal Carcinoma"[Title] OR "colorectal adenocarcinoma"[Title]) AND ("metastatic"[Title/Abstract] OR "advanced"[Title/Abstract]) AND "English"[Language] AND ("randomized controlled trial"[Publication Type] OR "controlled clinical trial"[Publication Type] OR "Randomized"[Title/Abstract] OR "randomised"[Title/Abstract] OR "randomly"[Title/Abstract] OR "Trial"[Title/Abstract] OR "phase"[Title/Abstract]))

# Supplementary File 3. Characteristics of the included Trials and definitions of primary chemotherapy and targeted therapies

Table S1 Characteristics of the included Trials

| Trial | Line | Regimen | | Number of patients | | | Age (Mean, range or SD) | | Sex (Male, %) | | ECOG（0/1%） | | Proportion of liver metastasis (%) | | Primary Outcome |
| --- | --- | --- | --- | --- | --- | --- | --- | --- | --- | --- | --- | --- | --- | --- | --- |
|  |  | T | C | T | C | | T | C | T | C | T | C | T | C |  |
| CAIRO5-Mu (1) | F | FOLFOX or FOLFIRI plus bevacizumab | FOLFOXIRI plus bevacizumab | 147 | 144 | | 61 (54-70) | 65 (57-70) | 64 | 60 | N | N | 100 | 100 | PFS |
| CAIRO5-WT (1) | F | FOLFOX or FOLFIRI plus bevacizumab | FOLFOX or FOLFIRI plus panitumumab | 114 | 116 | | 59 (53-67) | 60 (52-69) | 61 | 63 | N | N | 100 | 100 | PFS |
| PARADIGM (2) | F | Panitumumab+mFOLFOX6 | Bevacizumab+mFOLFOX6 | 400 | 402 | | 66.0 (32-79) | 66.0 (28-79) | 63 | 66.7 | 99.8 | 100 | 68.75 | 69.15 | PFS+OS |
| AIO KRK 0314 (3) | F+S | FOLFOX plus panitumumab | FOLFOX | 70 | 36 | | 61 (43-81) | 65 (44-77) | 71.4 | 86.1 | 98.6 | 100 | 100 | 100 | PFS+OS |
| TRIPLETE (4) | F | mFOLFOXIRI plus panitumumab | mFOLFOX plus panitumumab | 217 | 218 | | 59 (51-65) | 59 (51-64) | 64 | 62 | 99 | 99 | N | N | PFS |
| AtezoTRIBE (5) | F | FOLFOXIRI plus bevacizumab and atezolizumab | FOLFOXIRI plus bevacizumab | 145 | 73 | | 60 (52-67) | 61 (54-66) | 57 | 58 | 100 | 100 | N | N | PFS |
| FOCUS4-C (6) | M | Adavosertib | Active monitoring | 44 | 25 | | 59.2 (12.8) | 61.9 (12.2) | 70 | 60 | N | N | N | N | PFS |
| FOCUS4-N (7) | M | Capecitabine | Active monitoring | 127 | 127 | | 64.7 (9.6) | 63.7 (10.9) | 68 | 60 | N | N | N | N | PFS+OS |
| OBELICS (8) | F | Bevacizumab in combination With Standard Oxaliplatin-Based chemotherapy | modified FOLFOX-6/modified CAPOX | 115 | 115 | | 61 (53-68) | 63 (56-68) | 60 | 58.3 | 100 | 100 | N | N | PFS+OS |
| JCOG1007 (9) | F | Primary tumor resection plus mFOLFOX6 or CapeOX plus bevacizumab | mFOLFOX6 or CapeOX plus bevacizumab | 81 | 84 | | 65 (59-69) | 65 (59-71) | 56 | 54 | 76.5 | 76.2 | 74 | 71 | OS |
| Geng,2020 (10) | M | Capecitabine | Observation | 25 | 23 | | N | N | 60 | 59.1 | N | N | N | N | PFS+OS |
| FIRE-3 (11, 12) | F | FOLFIRI plus cetuximab | FOLFIRI plus bevacizumab | 297 | 295 | | 64 | 65 | 72.1 | 66.4 | 97.6 | 98.6 | 81.1 | 81.4 | OS |
| VISNÚ-1 (13) | F | bevacizumab plus FOLFOXIRI | Bevacizumab plus FOLFOX | 177 | 172 | | 59 (53-65) | 61 (54-66) | 67.2 | 68.6 | 100 | 100 | N | N | OS |
| TRIBE2 (14) | F+S | FOLFOXIRI plus bevacizumab and reintroduction after progression | mFOLFOX6 plus bevacizumab followed by FOLFIRI plus bevacizumab | 339 | 340 | | 60 (53-67) | 61 (52-67) | 53 | 61 | 100 | 100 | N | N | PFS+OS |
| NORDIC9 (15) | F | S-1 plus oxaliplatin | S-1 | 77 | 83 | | 78 (75-80) | 78 (76-81) | 51 | 52 | N | N | 57 | 70 | PFS+OS |
| TAILOR (16) | F | Cetuximab plus FOLFOX-4 | FOLFOX-4 | 193 | 200 | | 56(21-83) | 56(21-78) | 65.8 | 69.5 | 100 | 100 | N | N | PFS |
| IND.210 (17) | F | FOLFOX6+Bevacizumab+Pelareorep | FOLFOX6+Bevacizumab | 51 | 52 | | 60 (34-79) | 59 (31-78) | 63 | 60 | 96 | 96 | N | N | PFS |
| PRODIGE 9 (18) | M | Bevacizumab | Observation | 245 | 243 | | 64.2 (57.3, 72.3) | 65.0 (57.8, 72.7) | 62 | 67.5 | N | N | N | N | PFS+OS |
| TRICOLORE  (19, 20) | F | S-1 and irinotecan plus bevacizumab | mFOLFOX6 or CapeOX plus bevacizumab | 243 | 241 | | 65 (29-85) | 64 (22-87) | 58.8 | 62.7 | 100 | 100 | N | N | PFS |
| CAIRO3(21, 22) | M | Capecitabine and bevacizumab | Observation | 278 | 279 | | 63 (26-81) | 64 (31-81) | 65 | 64 | N | N | N | N | OS |
| BATON-CRC (23) | F | Tivozanib+mFOLFOX6 | Bevacizumab+mFOLFOX6 | 177 | 88 | | 61.9 (9.6) | 62.6 (11.2) | 66.7 | 62.5 | 100 | 100 | N | N | PFS |
| Luo,2016 (24) | F | Capecitabine | Observation | 136 | 138 | | 56 (27-78) | 54 (23-78) | 61.1 | 62.3 | 95.6 | 91.3 | 50.7 | 52.9 | PFS+OS |
| SIRFLOX,2016 (25) | F | SIRT+ mFOLFOX6 | mFOLFOX6 | 267 | 263 | | 63 (28-81) | 63 (23-89) | 68.2 | 66.2 | N | N | 72.3 | 92.2 | PFS |
| FFCD 2001-02 (26) | F | Fluorouracil | Irinotecan | 142 | 140 | | N | N | 52.8 | 54.3 | N | N | N | N | PFS+OS |
| OPTIMOX3  (27) | M | Bevacizumab+erlotinib | Bevacizumab | 224 | 228 | | 63 (57-70) | 63 (57-70) | 66 | 57 | N | N | 86 | 82 | PFS+OS |
| TRIBE  (28, 29) | F | FOLFOXIRI plus bevacizumab | FOLFIRI plus bevacizumab | 252 | 256 | | 60.5(29-75) | 60(29-75) | 59.5 | 60.9 | N | N | N | N | PFS+OS |
| OPUS (30-32) | F | FOLFOX4 plus cetuximab | FOLFOX4 | 169 | 168 | | 62(24-82) | 60(30-82) | 53 | 55 | 91 | 90 | 88 | 87 | PFS |
| FOXFIRE (33) | F | FOLFOX plus SIRT | FOLFOX | 554 | 549 | | 63.4 (28.-89.6) | 62.7 (23.1-89.0) | 66 | 66 | N | N | N | N | PFS+OS |
| SIRFLOX,2017 (33) | F | FOLFOX plus SIRT | FOLFOX | 554 | 549 | | 63.4 (28.-89.6) | 62.7 (23.1-89.0) | 66 | 66 | N | N | N | N | PFS+OS |
| FOXFIRE-Global (33) | F | FOLFOX plus SIRT | FOLFOX | 554 | 549 | | 63.4 (28.-89.6) | 62.7 (23.1-89.0) | 66 | 66 | N | N | N | N | PFS+OS |
| FIRE-4.5  (34) | F | FOLFOXIRI plus cetuximab | FOLFOXIRI plus bevacizumab | 72 | 35 | | 62 (31-78) | 64 (31-78) | 55.6 | 40 | 100 | 100 | 66.7 | 60 | PFS+OS |
| Cassidy, 2007 (35) | F | XELOX+bevacizumab | FOLFOX-4+bevacizumab | 1017 | 1017 | | N | N | 58.5 | 60.4 | 100 | 99.9 | N | N | PFS |
| MRC FOCUS (36) | F | Fluorouracil plus irinotecan | Fluorouracil plus oxaliplatin | 709 | | 711 | N | N | 67.7 | 68.6 | N | N | 76 | 77.9 | OS |
| PRODIGE-14 (37) | F | Intensified CTFU plus bevacizumab/cetuximab by RAS | CTFU plus bevacizumab/cetuximab by RAS | 130 | 126 | | 60 (27-78) | 61 (29-75) | 62.3 | 65.1 | 100 | 100 | 100 | 100 | PFS+OS |
| HU 2020 (38) | F | Cetuximab plus Intensified CTFU | Intensified CTFU | 67 | 34 | | 52(28-70) | 55(29-70) | 86.6 | 85.3 | 100 | 100 | 100 | 100 | PFS |
| ATOM (39) | F | CTFU plus Cetuximab | CTFU plus Bevacizumab | 61 | 61 | | 65 (42.0-79.0) | 64 (32.0-80.0) | 57.6 | 59.6 | 100 | 100 | 100 | 100 | PFS+OS |
| Ruers 2017 (40) | F | Local treatment plus CTFU | CTFU | 60 | 59 | | 64 (31-79) | 61 (38-79) | 61.7 | 71.2 | N | N | 100 | 100 | PFS+OS |
| OLIVIA (41) | F | Bevacizumab plus Intensified CTFU | Bevacizumab plus CTFU | 41 | 39 | | 63(32-77) | 57(28-80) | 71 | 46 | 95 | 100 | 100 | 100 | PFS |
| METHEP (42) | F | Intensified CTFU | CTFU | 92 | 30 | | N | 62.5(47-73) | 58.7 | 50 | N | N | 100 | 100 | PFS+OS |
| BECOME  (43) | F | Bevacizumab plus CTFU | CTFU | 121 | 120 | | 58 (29-75) | 59 (24-72) | 65.3 | 66.7 | 100 | 100 | 100 | 100 | PFS+OS |
| Ye 2013  (44) | F | Cetuximab plus CTFU | CTFU | 70 | 68 | | 57(26-75) | 59(35-75) | 65.7 | 61.8 | 100 | 100 | 100 | 100 | PFS+OS |
| FFCD 9601 (45) | F | Local treatment plus 5-fluorouracil | 5-fluorouracil | 156 | 60 | | 64 (±8.2) | 62 (±10) | 62 | 73 | N | N | 100 | 100 | PFS+OS |
| EORTC 40004 (46) | F | Local treatment plus CTFU | CTFU | 60 | 59 | | 64 (31-79) | 61 (38-79) | 61.7 | 71.2 | N | N | 100 | 100 | PFS+OS |
| FFCD 2000-05 (47) | F | CTFU | fluorouracil/leucovorin | 205 | 205 | | 68 (57-73) | 66 (56-72) | 63 | 60 | N | N | 91 | 86 | PFS+OS |
| Skof,2009 (48) | F | CTCA | FOLFIRI | 41 | 46 | | 63 (47-75) | 62 (34-75) | 63 | 59 | N | N | 100 | 100 | N |
| Rougier,1992 (49) | FS | HAI | fluorouracil | 81 | 82 | | 59±8 | 61±10 | 58 | 54 | N | N | 100 | 100 | OS |
| Hunt,1990  (50) | FS | HAI | BSC | 19 | 20 | | N | N | N | N | N | N | 100 | 100 | OS |
| PRIME  (51, 52) | F | Panitumumab plus CTFU | CTFU | 325 | 331 | | 62 (27, 85) | 61 (24, 82) | 67 | 62 | 94 | 94 | 68 | 69 | PFS |
| Lee2012(53) | F | TSU-68 plus S-1 plus oxaliplatin | S-1 plus oxaliplatin | 52 | 53 | | 58.1( 31.0-79.0) | 58.1(28.0-74.0) | 61.5 | 73.6 | 100 | 100 | N | N | PFS |
| The GOLFIG-2 (54) | F | GOLFIG | CTFU | 62 | 62 | | 66 (27-82) | 68 (37-83) | 39 | 45 | 85.5 | 87.1 | 53.2 | 30.6 | PFS |
| SOFT  (55, 56) | F | Bevacizumab plus CTFU | S-1 plus oxaliplatin plus bevacizumab | 255 | 256 | | 63 (39-79) | 63 (33-79) | 62 | 66 | N | N | 64 | 66 | PFS+OS |
| Douillard 2013 (57) | F | Panitumumab plus CTFU | CTFU | 259 | 253 | | N | N | N | N | N | N | N | N | PFS+OS |
| RESPECT  (58) | F | Sorafenib plus CTFU | CTFU | 97 | 101 | | 59.2 (33-82) | 60.3 (44-77) | 43.3 | 62.4 | 100 | 100 | 81.4 | 80.2 | PFS+OS |
| Hong 2012  (59) | F | S-1 plus oxaliplatin | CTCA | 168 | 172 | | 61 (53-66) | 60 (52-66) | 65 | 59 | 98 | 98 | 63 | 65 | PFS+OS |
| HORIZON II (60) | F | FOLFOX/CAPOX + cediranib | FOLFOX/CAPOX | 502 | 358 | | 58(2-83) | 59( 22-82) | 59.6 | 59.2 | N | N | N | N | PFS |
| MRC COIN (61) | F | Cetuximab plus XELOX | XELOX | 815 | 815 | | 63 (58-70) | 63 (56-69) | 67 | 64 | N | N | 51 | 53 | PFS+OS |
| Kab 2005 (62) | F | Bevacizumab plus fluorouracil/leucovorin | fluorouracil/leucovorin | 104 | 105 | | 71.3 | 70.7 | 56 | 51 | 92 | 94 | N | N | PFS+OS |

Abbreviation: BSC, best supportive care; C, control group; CTFU, fluorouracil-based combination chemotherapy; CTCA, capecitabine-based combination chemotherapy; F, first-line treatment; ICTFU, intensified fluorouracil-based combination chemotherapy; OS, overall survival; PFS, progression-free survival; M, maintenance treatment; N, not mentioned; S, second-line treatment; SIRT, Selective internal radiation therapy; T, treatment group.

Table S2 Definitions of primary chemotherapy and targeted therapies

| Treatment | Medication details |
| --- | --- |
| Bevacizumab | 5 mg/kg |
| Capecitabine | 750 mg/m2,twice daily |
| Cetuximab | 500 mg/m2 |
| FOLFIRI | irinotecan 180 mg/m2 on day 1, leucovorin 200 mg/m2 on days 1 and 2, followed by 5-fluorouracil 400 mg/m2 bolus and then 5-fluorouracil 600 mg/m2 both on days 1 and 2, every two weeks |
| FOLFOX/FLOX | every 2 weeks with oxaliplatin 85 mg/m2 on day 1, and 5-fluorouracil 500 mg/m2 bolus injection followed 30 min later by FA 60 mg/m2 bolus on days 1 and 2 |
| FOLFOX-4 | oxaliplatin, 85 mg/m2 at day 1, and leucovorin calcium, 200 mg/m2, and fluorouracil, 400-mg/m2 bolus followed by 600-mg/m2 continuous 24-hour influorouracilsion at days 1 and 2, every 2 weeks |
| Fluorouracil | 2,600 mg/m2 as a 24-hour influorouracilsion alone weekly |
| Fluorouracil+leucovorin | Fluorouracil 425 mg/m2 i.v. weekly; leucovorin 20 mg/m2 on days 1 to 5 and repeated on day 28 |
| Irinotecan | 300 or 350 mg/m2 every 3 weeks |
| modified CAPOX/XELOX | intravenous oxaliplatin, 85 mg/m2, on day 1 plus oral capecitabine, 1000 mg/m2, twice daily on days 1 to 10) |
| modified FOLFOX-6 | intravenous oxaliplatin, 85 mg/m2, on day 1, followed by intravenous levo–folinic acid, 200 mg/m 2,plus bolus fluorouracil, 400 mg/m 2, and a 46-hour intravenous administration of fluorouracil, 2400mg/m2 |
| OXA(OXAFAFU) | Oxaliplatin 85 mg/m2 i.v. on day 1; 6S-leucovo rin 250 mg/m2 i.v. (2 h), followed by fluorouracil 850 mg/m2 i.v. bolus on day 2, every 2 weeks |
| oxaliplatin | 85 mg/m2, per two weeks |
| OXXEL | oxaliplatin 100 mg/m2 i.v on day 1; capecitabine 1,000 mg/m2 orally twice daily from day 1 to day 11,every 2 weeks |
| Panitumumab | 6 mg/kg [1-hour influorouracilsion for the first administration; 30-minute influorouracilsion thereafter] |
| S1 | fluorouracilll-dose: 30 mg/m2 orally twice daily on days 1–14,q3w;reduced-dose S1: 20 mg/m2 orally twice daily on days 1–14,q3w |
| SIRT | Selective internal radiotherapy (SIRT) is an involeucovorining injection of yttrium-90 microspheres |

# Supplementary File 4. Cochrane risk of the bias assessment tool


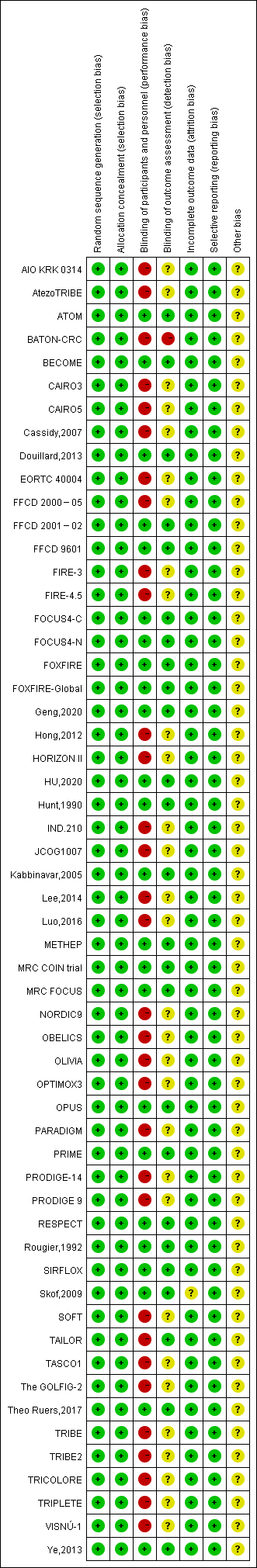


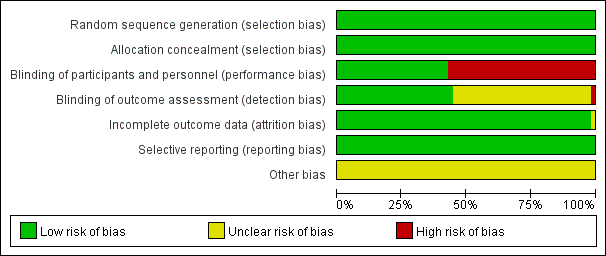


Supplementary File 5. Funnel plots to show publication bias

| OS | PFS |
| --- | --- |
| 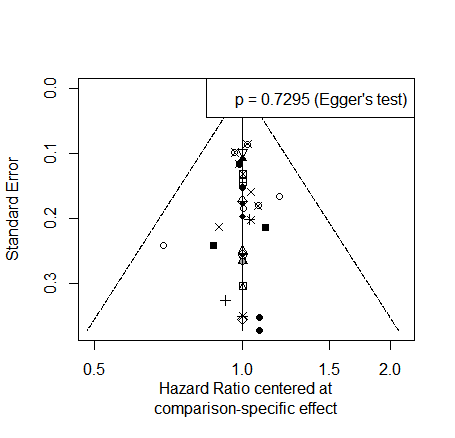 | 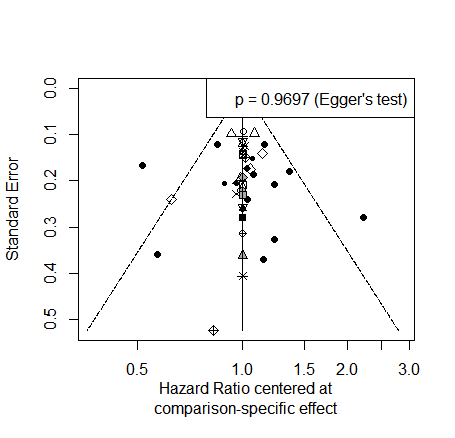 |

# Supplementary File 6. Additional results of primary analyses for first-line treatments

Figure S1 Forest plots illustrating the comparison results of first-line treatment mechanism


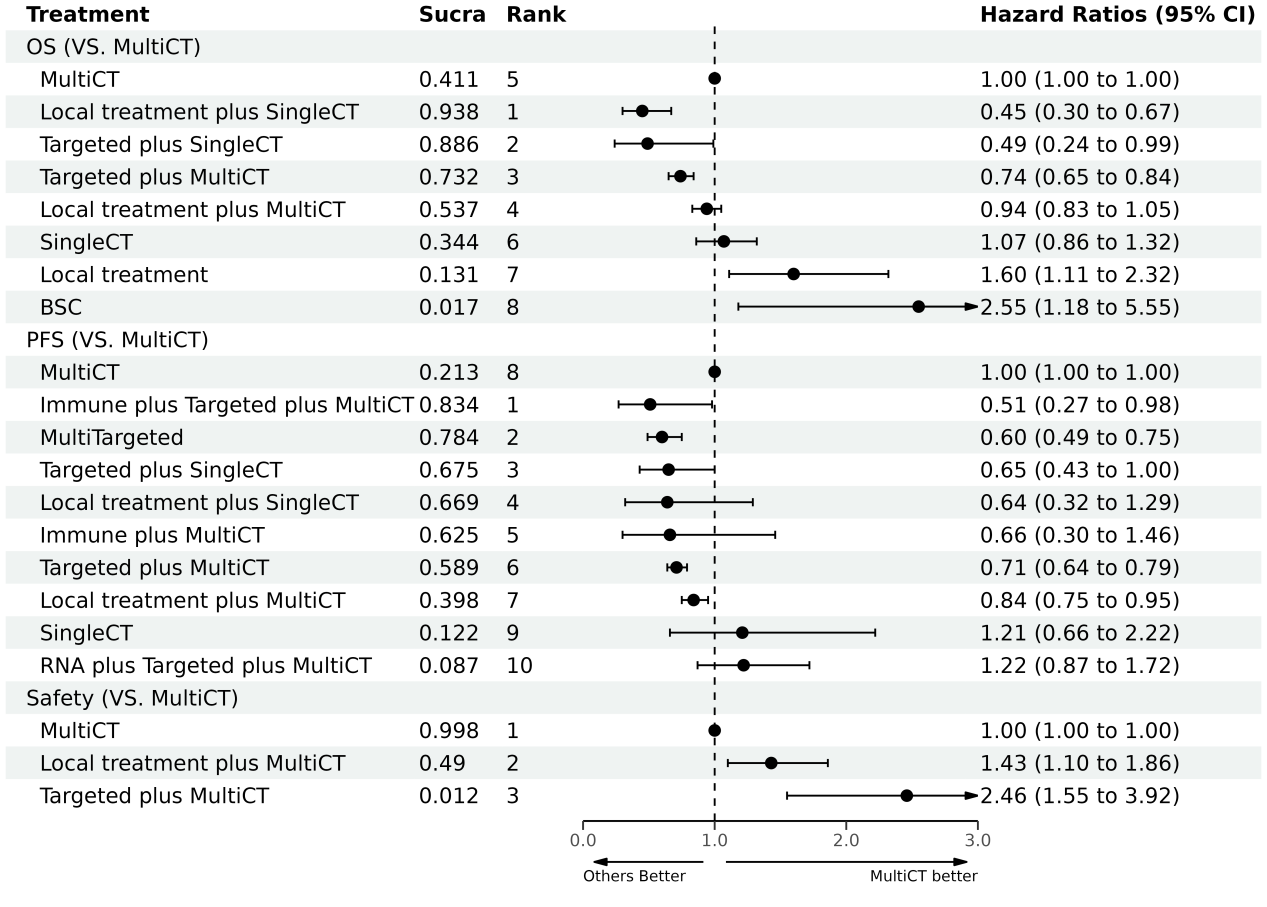


Abbreviation: BSC, best supportive care; CTFU, fluorouracil-based combination chemotherapy; CTCA, capecitabine-based combination chemotherapy; ICTFU, intensified fluorouracil-based combination chemotherapy; Immune, Immune checkpoint inhibitor; MultiCT, multi-drug chemotherapy; MultiTargeted, multi-targeted therapy; SingleCT, single-drug chemotherapy; Targeted, targeted therapy.

Table S3 League table for overall survival and progression-free survival

| Progression-free Survival | Overall Survival | | | | | | | | | | | | | | | | | | | | | | | | | | | |
| --- | --- | --- | --- | --- | --- | --- | --- | --- | --- | --- | --- | --- | --- | --- | --- | --- | --- | --- | --- | --- | --- | --- | --- | --- | --- | --- | --- | --- |
|  | ATE+BEV+ICTFU | N | N | N | N | N | N | N | N | N | N | N | N | N | N | N | N | N | N | N | N | N | N | N | N | N | N | N |
|  | 0.46 (0.22, 0.98) | BEV+CTCA | N | N | N | N | N | N | N | N | N | N | N | N | N | N | N | N | N | N | N | N | N | N | N | N | N | N |
|  | 0.55 (0.28, 1.08) | 1.19 (0.85, 1.66) | BEV+CTFU | 0.77 (0.62, 0.96) | 0.66 (0.33, 1.34) | 3.39 (1.53, 7.47) | N | 0.75 (0.56, 0.99) | 0.7 (0.45, 1.09) | 1.05 (0.61, 1.79) | 1.33 (1.13, 1.56) | 1.38 (1.12, 1.7) | 2.13 (1.43, 3.17) | N | 0.97 (0.53, 1.8) | 0.86 (0.63, 1.18) | 0.6 (0.39, 0.92) | 0.91 (0.77, 1.06) | N | N | 0.61 (0.28, 1.36) | 1.08 (0.83, 1.39) | 0.93 (0.51, 1.71) | 1.42 (1.09, 1.85) | 1.41 (0.93, 2.14) | N | N | N |
|  | 0.72 (0.38, 1.37) | 1.55 (1.06, 2.28) | 1.3 (1.07, 1.57) | BEV+ICTFU | 0.86 (0.41, 1.79) | 4.38 (1.92, 9.94) | N | 0.97 (0.68, 1.38) | 0.91 (0.56, 1.48) | 1.36 (0.76, 2.42) | 1.72 (1.32, 2.25) | 1.78 (1.32, 2.4) | 2.76 (1.75, 4.33) | N | 1.26 (0.66, 2.41) | 1.11 (0.76, 1.63) | 0.77 (0.47, 1.26) | 1.17 (0.9, 1.54) | N | N | 0.79 (0.35, 1.82) | 1.39 (1, 1.95) | 1.21 (0.63, 2.31) | 1.84 (1.31, 2.58) | 1.82 (1.14, 2.92) | N | N | N |
|  | 0.72 (0.27, 1.91) | 1.54 (0.69, 3.42) | 1.29 (0.62, 2.67) | 0.99 (0.47, 2.1) | BEV+SCT | 5.12 (1.77, 14.75) | N | 1.13 (0.53, 2.42) | 1.06 (0.46, 2.44) | 1.59 (0.65, 3.84) | 2.01 (0.97, 4.13) | 2.08 (1, 4.33) | 3.22 (1.43, 7.26) | N | 1.47 (0.58, 3.74) | 1.3 (0.6, 2.82) | 0.9 (0.39, 2.07) | 1.37 (0.66, 2.83) | N | N | 0.93 (0.32, 2.7) | 1.63 (0.77, 3.46) | 1.41 (0.56, 3.58) | 2.15 (1.01, 4.56) | 2.13 (0.94, 4.82) | N | N | N |
|  | N | N | N | N | N | BSC | N | 0.22 (0.1, 0.51) | 0.21 (0.08, 0.51) | 0.31 (0.12, 0.78) | 0.39 (0.18, 0.85) | 0.41 (0.18, 0.89) | 0.63 (0.32, 1.25) | N | 0.29 (0.11, 0.76) | 0.25 (0.11, 0.58) | 0.18 (0.08, 0.4) | 0.27 (0.12, 0.59) | N | N | 0.18 (0.06, 0.55) | 0.32 (0.14, 0.73) | 0.28 (0.1, 0.73) | 0.42 (0.2, 0.88) | 0.42 (0.17, 0.99) | N | N | N |
|  | 0.3 (0.13, 0.73) | 0.66 (0.34, 1.25) | 0.55 (0.32, 0.96) | 0.42 (0.24, 0.76) | 0.43 (0.17, 1.06) | N | CED+CTCA | N | N | N | N | N | N | N | N | N | N | N | N | N | N | N | N | N | N | N | N | N |
|  | 0.61 (0.3, 1.23) | 1.31 (0.87, 1.96) | 1.1 (0.87, 1.38) | 0.84 (0.62, 1.14) | 0.85 (0.4, 1.82) | N | 1.99 (1.13, 3.51) | CET+CTFU | 0.94 (0.67, 1.32) | 1.4 (0.77, 2.53) | 1.77 (1.32, 2.39) | 1.84 (1.32, 2.55) | 2.85 (1.78, 4.56) | N | 1.3 (0.67, 2.52) | 1.15 (0.76, 1.73) | 0.8 (0.48, 1.32) | 1.21 (0.89, 1.66) | N | N | 0.82 (0.35, 1.9) | 1.44 (0.98, 2.1) | 1.25 (0.65, 2.41) | 1.9 (1.32, 2.73) | 1.88 (1.16, 3.06) | N | N | N |
|  | 0.59 (0.28, 1.24) | 1.27 (0.8, 2.02) | 1.06 (0.77, 1.48) | 0.82 (0.56, 1.19) | 0.82 (0.37, 1.83) | N | 1.93 (1.05, 3.56) | 0.97 (0.75, 1.25) | CET+ICTFU | 1.49 (0.75, 2.94) | 1.89 (1.2, 2.96) | 1.95 (1.22, 3.14) | 3.03 (1.69, 5.42) | N | 1.38 (0.65, 2.91) | 1.23 (0.72, 2.08) | 0.85 (0.46, 1.55) | 1.29 (0.81, 2.04) | N | N | 0.87 (0.35, 2.15) | 1.53 (0.92, 2.55) | 1.33 (0.63, 2.77) | 2.02 (1.22, 3.32) | 2 (1.1, 3.62) | N | N | N |
|  | 0.31 (0.14, 0.71) | 0.68 (0.38, 1.21) | 0.57 (0.35, 0.91) | 0.44 (0.26, 0.73) | 0.44 (0.18, 1.05) | N | 1.03 (0.78, 1.37) | 0.52 (0.32, 0.85) | 0.53 (0.31, 0.92) | CTCA | 1.27 (0.76, 2.11) | 1.31 (0.77, 2.23) | 2.03 (1.08, 3.82) | N | 0.93 (0.42, 2.03) | 0.82 (0.46, 1.48) | 0.57 (0.3, 1.1) | 0.87 (0.51, 1.48) | N | N | 0.59 (0.32, 1.06) | 1.03 (0.57, 1.87) | 0.89 (0.67, 1.18) | 1.36 (0.78, 2.36) | 1.34 (0.71, 2.55) | N | N | N |
|  | 0.35 (0.17, 0.68) | 0.74 (0.51, 1.08) | 0.62 (0.54, 0.73) | 0.48 (0.38, 0.61) | 0.48 (0.23, 1.02) | N | 1.13 (0.67, 1.93) | 0.57 (0.47, 0.69) | 0.59 (0.43, 0.79) | 1.1 (0.7, 1.73) | CTFU | 1.04 (0.9, 1.19) | 1.61 (1.12, 2.31) | N | 0.73 (0.4, 1.32) | 0.65 (0.49, 0.87) | 0.45 (0.3, 0.67) | 0.68 (0.58, 0.81) | N | N | 0.46 (0.21, 1.01) | 0.81 (0.6, 1.1) | 0.7 (0.39, 1.26) | 1.07 (0.87, 1.32) | 1.06 (0.72, 1.56) | N | N | N |
|  | 0.38 (0.19, 0.77) | 0.83 (0.56, 1.22) | 0.69 (0.57, 0.85) | 0.53 (0.4, 0.7) | 0.54 (0.25, 1.14) | N | 1.26 (0.73, 2.18) | 0.63 (0.5, 0.8) | 0.65 (0.47, 0.9) | 1.22 (0.76, 1.95) | 1.11 (0.98, 1.26) | CTFU+SIRT | 1.55 (1.05, 2.29) | N | 0.71 (0.38, 1.3) | 0.63 (0.46, 0.86) | 0.43 (0.28, 0.67) | 0.66 (0.53, 0.82) | N | N | 0.45 (0.2, 0.99) | 0.78 (0.56, 1.09) | 0.68 (0.37, 1.24) | 1.03 (0.8, 1.33) | 1.02 (0.68, 1.54) | N | N | N |
|  | 0.52 (0.18, 1.5) | 1.13 (0.46, 2.71) | 0.94 (0.42, 2.13) | 0.72 (0.32, 1.67) | 0.73 (0.25, 2.18) | N | 1.71 (0.66, 4.48) | 0.86 (0.38, 1.96) | 0.89 (0.38, 2.08) | 1.66 (0.67, 4.16) | 1.51 (0.68, 3.36) | 1.36 (0.61, 3.06) | GOLFIG | N | N | N | N | N | N | N | N | N | N | N | N | N | N | N |
|  | N | N | N | N | N | N | N | N | N | N | N | N | N | HAI | 0.46 (0.23, 0.91) | 0.4 (0.25, 0.64) | 0.28 (0.18, 0.44) | 0.43 (0.29, 0.64) | N | N | 0.29 (0.12, 0.68) | 0.51 (0.31, 0.81) | 0.44 (0.22, 0.87) | 0.67 (0.49, 0.9) | 0.66 (0.39, 1.12) | N | N | N |
|  | 0.45 (0.21, 0.95) | 0.97 (0.6, 1.55) | 0.81 (0.58, 1.13) | 0.62 (0.42, 0.92) | 0.63 (0.28, 1.4) | N | 1.47 (0.8, 2.71) | 0.74 (0.53, 1.03) | 0.76 (0.54, 1.08) | 1.43 (0.83, 2.45) | 1.3 (0.96, 1.75) | 1.17 (0.84, 1.62) | 0.86 (0.37, 2.01) | N | ICTFU | 0.89 (0.46, 1.71) | 0.61 (0.3, 1.26) | 0.93 (0.5, 1.73) | N | N | 0.63 (0.24, 1.69) | 1.11 (0.57, 2.16) | 0.96 (0.42, 2.21) | 1.46 (0.78, 2.75) | 1.45 (0.71, 2.94) | N | N | N |
|  | 0.58 (0.27, 1.21) | 1.24 (0.78, 1.98) | 1.04 (0.75, 1.44) | 0.8 (0.55, 1.17) | 0.81 (0.36, 1.79) | N | 1.89 (1.03, 3.47) | 0.95 (0.67, 1.34) | 0.98 (0.65, 1.48) | 1.84 (1.08, 3.13) | 1.67 (1.25, 2.22) | 1.5 (1.1, 2.06) | 1.1 (0.47, 2.58) | N | 1.29 (0.85, 1.94) | R/A+CTFU | 0.69 (0.42, 1.14) | 1.05 (0.76, 1.46) | N | N | 0.71 (0.31, 1.64) | 1.25 (0.83, 1.88) | 1.08 (0.56, 2.09) | 1.65 (1.15, 2.36) | 1.63 (1.01, 2.65) | N | N | N |
|  | 0.73 (0.24, 2.26) | 1.57 (0.59, 4.13) | 1.32 (0.53, 3.27) | 1.01 (0.4, 2.57) | 1.02 (0.59, 1.77) | N | 2.39 (0.82, 6.94) | 1.2 (0.47, 3.06) | 1.24 (0.47, 3.25) | 2.32 (0.83, 6.47) | 2.11 (0.84, 5.3) | 1.9 (0.74, 4.81) | 1.39 (0.41, 4.72) | N | 1.62 (0.61, 4.27) | 1.26 (0.48, 3.31) | R/A+SCT | 1.52 (0.98, 2.36) | N | N | 1.03 (0.43, 2.48) | 1.81 (1.09, 3) | 1.56 (0.76, 3.18) | 2.38 (1.68, 3.37) | 2.36 (1.35, 4.13) | N | N | N |
|  | 0.49 (0.25, 0.97) | 1.05 (0.73, 1.52) | 0.88 (0.76, 1.03) | 0.68 (0.53, 0.87) | 0.68 (0.33, 1.44) | N | 1.6 (0.92, 2.79) | 0.81 (0.63, 1.02) | 0.83 (0.59, 1.16) | 1.56 (0.97, 2.5) | 1.41 (1.22, 1.65) | 1.27 (1.04, 1.56) | 0.94 (0.42, 2.11) | N | 1.09 (0.78, 1.52) | 0.85 (0.61, 1.17) | 0.67 (0.27, 1.69) | PAN+CTFU | N | N | 0.68 (0.3, 1.5) | 1.19 (0.88, 1.61) | 1.03 (0.56, 1.89) | 1.57 (1.2, 2.05) | 1.55 (1.02, 2.36) | N | N | N |
|  | 0.52 (0.24, 1.14) | 1.12 (0.66, 1.9) | 0.94 (0.62, 1.42) | 0.72 (0.46, 1.13) | 0.73 (0.32, 1.68) | N | 1.71 (0.88, 3.34) | 0.86 (0.55, 1.34) | 0.88 (0.53, 1.46) | 1.66 (0.9, 3.04) | 1.51 (1, 2.27) | 1.36 (0.88, 2.08) | 1 (0.41, 2.44) | N | 1.16 (0.7, 1.92) | 0.9 (0.55, 1.48) | 0.71 (0.26, 1.95) | 1.06 (0.73, 1.56) | PAN+ICTFU | N | N | N | N | N | N | N | N | N |
|  | 0.32 (0.15, 0.68) | 0.69 (0.43, 1.11) | 0.58 (0.42, 0.81) | 0.45 (0.31, 0.65) | 0.45 (0.2, 1) | N | 1.06 (0.55, 2.01) | 0.53 (0.35, 0.79) | 0.55 (0.34, 0.87) | 1.03 (0.57, 1.83) | 0.93 (0.65, 1.34) | 0.84 (0.57, 1.23) | 0.62 (0.26, 1.48) | N | 0.72 (0.45, 1.14) | 0.56 (0.35, 0.88) | 0.44 (0.17, 1.17) | 0.66 (0.46, 0.95) | 0.62 (0.37, 1.04) | PEL+BEV+CTFU | N | N | N | N | N | N | N | N |
|  | 0.6 (0.2, 1.82) | 1.29 (0.49, 3.34) | 1.08 (0.44, 2.64) | 0.83 (0.33, 2.07) | 0.84 (0.26, 2.64) | N | 1.96 (0.87, 4.4) | 0.98 (0.4, 2.42) | 1.01 (0.4, 2.57) | 1.9 (0.89, 4.06) | 1.73 (0.71, 4.16) | 1.56 (0.64, 3.78) | 1.14 (0.35, 3.77) | N | 1.33 (0.53, 3.37) | 1.04 (0.41, 2.62) | 0.82 (0.23, 2.94) | 1.22 (0.5, 2.98) | 1.15 (0.43, 3.03) | 1.85 (0.71, 4.83) | S1 | 1.76 (0.76, 4.07) | 1.52 (0.9, 2.56) | 2.31 (1.03, 5.22) | 2.29 (0.96, 5.51) | N | N | N |
|  | 0.6 (0.3, 1.23) | 1.3 (1.02, 1.65) | 1.09 (0.86, 1.38) | 0.84 (0.62, 1.13) | 0.84 (0.39, 1.81) | N | 1.98 (1.09, 3.61) | 0.99 (0.71, 1.38) | 1.02 (0.68, 1.53) | 1.92 (1.13, 3.26) | 1.75 (1.32, 2.31) | 1.57 (1.15, 2.14) | 1.15 (0.5, 2.69) | N | 1.34 (0.89, 2.02) | 1.05 (0.7, 1.56) | 0.83 (0.32, 2.12) | 1.23 (0.93, 1.64) | 1.16 (0.72, 1.86) | 1.87 (1.25, 2.8) | 1.01 (0.4, 2.55) | S1+BEV+SCT | 0.87 (0.45, 1.67) | 1.52 (0.82, 2.84) | 1.51 (0.75, 3.04) | N | N | N |
|  | 0.38 (0.16, 0.91) | 0.82 (0.43, 1.57) | 0.69 (0.4, 1.2) | 0.53 (0.3, 0.95) | 0.54 (0.22, 1.33) | N | 1.26 (0.85, 1.87) | 0.63 (0.36, 1.11) | 0.65 (0.35, 1.2) | 1.22 (0.92, 1.61) | 1.11 (0.65, 1.88) | 1 (0.58, 1.72) | 0.73 (0.28, 1.91) | N | 0.85 (0.47, 1.56) | 0.66 (0.36, 1.21) | 0.53 (0.18, 1.53) | 0.78 (0.45, 1.36) | 0.74 (0.38, 1.43) | 1.19 (0.63, 2.26) | 0.64 (0.32, 1.3) | 0.63 (0.35, 1.15) | S1+SCT | 1.32 (0.91, 1.91) | 1.31 (0.8, 2.13) | N | N | N |
|  | 0.39 (0.13, 1.13) | 0.83 (0.33, 2.06) | 0.7 (0.3, 1.62) | 0.54 (0.22, 1.27) | 0.54 (0.35, 0.83) | N | 1.27 (0.46, 3.47) | 0.64 (0.26, 1.53) | 0.66 (0.26, 1.62) | 1.23 (0.46, 3.23) | 1.12 (0.47, 2.63) | 1.01 (0.42, 2.39) | 0.74 (0.23, 2.39) | N | 0.86 (0.35, 2.13) | 0.67 (0.27, 1.65) | 0.53 (0.38, 0.74) | 0.79 (0.33, 1.86) | 0.74 (0.29, 1.9) | 1.2 (0.49, 2.97) | 0.65 (0.19, 2.22) | 0.64 (0.27, 1.54) | 1.01 (0.37, 2.76) | SCT | 0.99 (0.64, 1.54) | N | N | N |
|  | 0.4 (0.19, 0.87) | 0.87 (0.52, 1.45) | 0.73 (0.49, 1.08) | 0.56 (0.36, 0.86) | 0.56 (0.25, 1.28) | N | 1.32 (0.69, 2.51) | 0.66 (0.44, 1) | 0.68 (0.43, 1.09) | 1.28 (0.72, 2.28) | 1.16 (0.81, 1.67) | 1.05 (0.71, 1.54) | 0.77 (0.32, 1.85) | N | 0.9 (0.56, 1.43) | 0.7 (0.44, 1.1) | 0.55 (0.2, 1.49) | 0.82 (0.55, 1.22) | 0.77 (0.45, 1.33) | 1.25 (0.75, 2.08) | 0.67 (0.26, 1.75) | 0.67 (0.42, 1.05) | 1.05 (0.55, 1.99) | 1.04 (0.41, 2.65) | SOR+CTFU | N | N | N |
|  | 0.83 (0.3, 2.25) | 1.79 (0.79, 4.06) | 1.5 (0.71, 3.17) | 1.16 (0.53, 2.5) | 1.16 (0.97, 1.39) | N | 2.73 (1.08, 6.9) | 1.37 (0.62, 2.99) | 1.41 (0.62, 3.19) | 2.65 (1.09, 6.41) | 2.41 (1.12, 5.15) | 2.17 (1, 4.69) | 1.59 (0.53, 4.81) | N | 1.85 (0.82, 4.2) | 1.44 (0.64, 3.26) | 1.14 (0.64, 2.03) | 1.7 (0.79, 3.65) | 1.6 (0.68, 3.75) | 2.58 (1.14, 5.84) | 1.39 (0.44, 4.46) | 1.38 (0.63, 3.02) | 2.17 (0.86, 5.47) | 2.15 (1.35, 3.43) | 2.07 (0.89, 4.8) | TAS102+BEV | N | N |
|  | 0.59 (0.27, 1.3) | 1.28 (0.76, 2.17) | 1.07 (0.72, 1.61) | 0.83 (0.53, 1.29) | 0.83 (0.36, 1.91) | N | 1.95 (0.98, 3.87) | 0.98 (0.62, 1.56) | 1.01 (0.6, 1.7) | 1.89 (1.01, 3.53) | 1.72 (1.12, 2.65) | 1.55 (0.99, 2.44) | 1.14 (0.46, 2.83) | N | 1.32 (0.78, 2.23) | 1.03 (0.61, 1.73) | 0.82 (0.3, 2.21) | 1.22 (0.79, 1.87) | 1.14 (0.64, 2.03) | 1.85 (1.1, 3.11) | 0.99 (0.37, 2.66) | 0.99 (0.62, 1.58) | 1.55 (0.78, 3.07) | 1.54 (0.6, 3.93) | 1.48 (0.84, 2.6) | 0.72 (0.31, 1.67) | TIV+CTFU | N |
|  | 0.32 (0.11, 0.92) | 0.69 (0.28, 1.66) | 0.57 (0.25, 1.31) | 0.44 (0.19, 1.03) | 0.44 (0.15, 1.33) | N | 1.04 (0.5, 2.17) | 0.52 (0.23, 1.21) | 0.54 (0.23, 1.28) | 1.01 (0.52, 1.99) | 0.92 (0.41, 2.06) | 0.83 (0.36, 1.88) | 0.61 (0.2, 1.9) | N | 0.71 (0.3, 1.68) | 0.55 (0.23, 1.3) | 0.44 (0.13, 1.49) | 0.65 (0.28, 1.48) | 0.61 (0.25, 1.51) | 0.99 (0.4, 2.4) | 0.53 (0.21, 1.36) | 0.53 (0.22, 1.24) | 0.83 (0.45, 1.53) | 0.82 (0.25, 2.69) | 0.79 (0.32, 1.92) | 0.38 (0.13, 1.16) | 0.53 (0.21, 1.33) | TSU68+S1+SCT |

Abbreviation: ATE, Atezolizumab; BEV, Bevacizumab; BSC, best supportive care; CED, Cediranib; CET, Cetuximab; CTFU, fluorouracil-based combination chemotherapy; CTCA, capecitabine-based combination chemotherapy; HAI, hepatic artery infusion; ICTFU, intensified fluorouracil-based combination chemotherapy; N, not available; PAN, panitumuma; PEL, pelareorep; R/A, resection or ablation; SIRT, Selective internal radiation therapy; SCT, single-drug chemotherapy; SOR, sorafenib.

Figure S2 Network plots for objective response rate, adverse events graded as 3 or above, and R0 liver resection rate

| A Safety | B ORR | C R0 liver resection rate |
| --- | --- | --- |
| 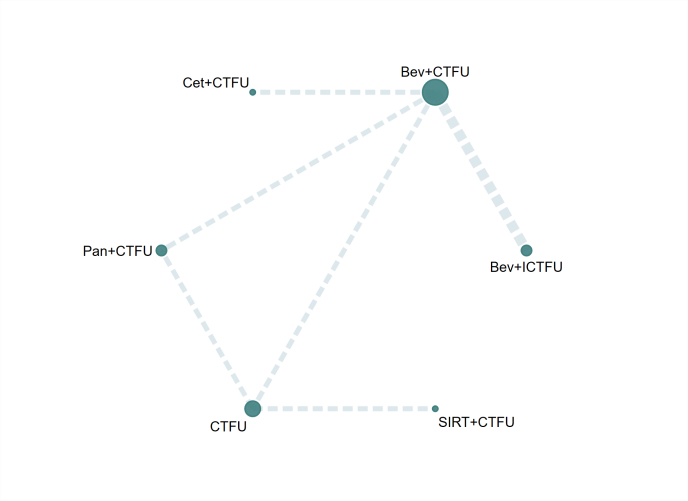 | 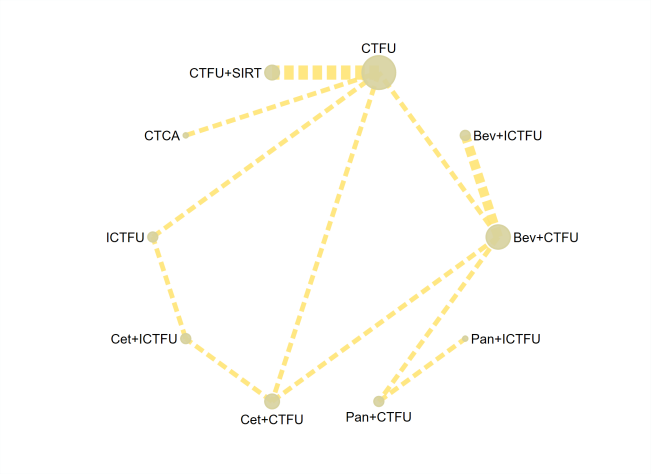 | 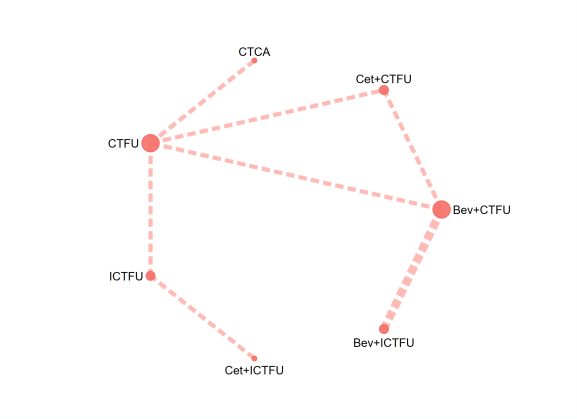 |

Figure S3 Forest plots illustrating the comparison results of objective response rate, adverse events graded as 3 or above, and R0 liver resection rate


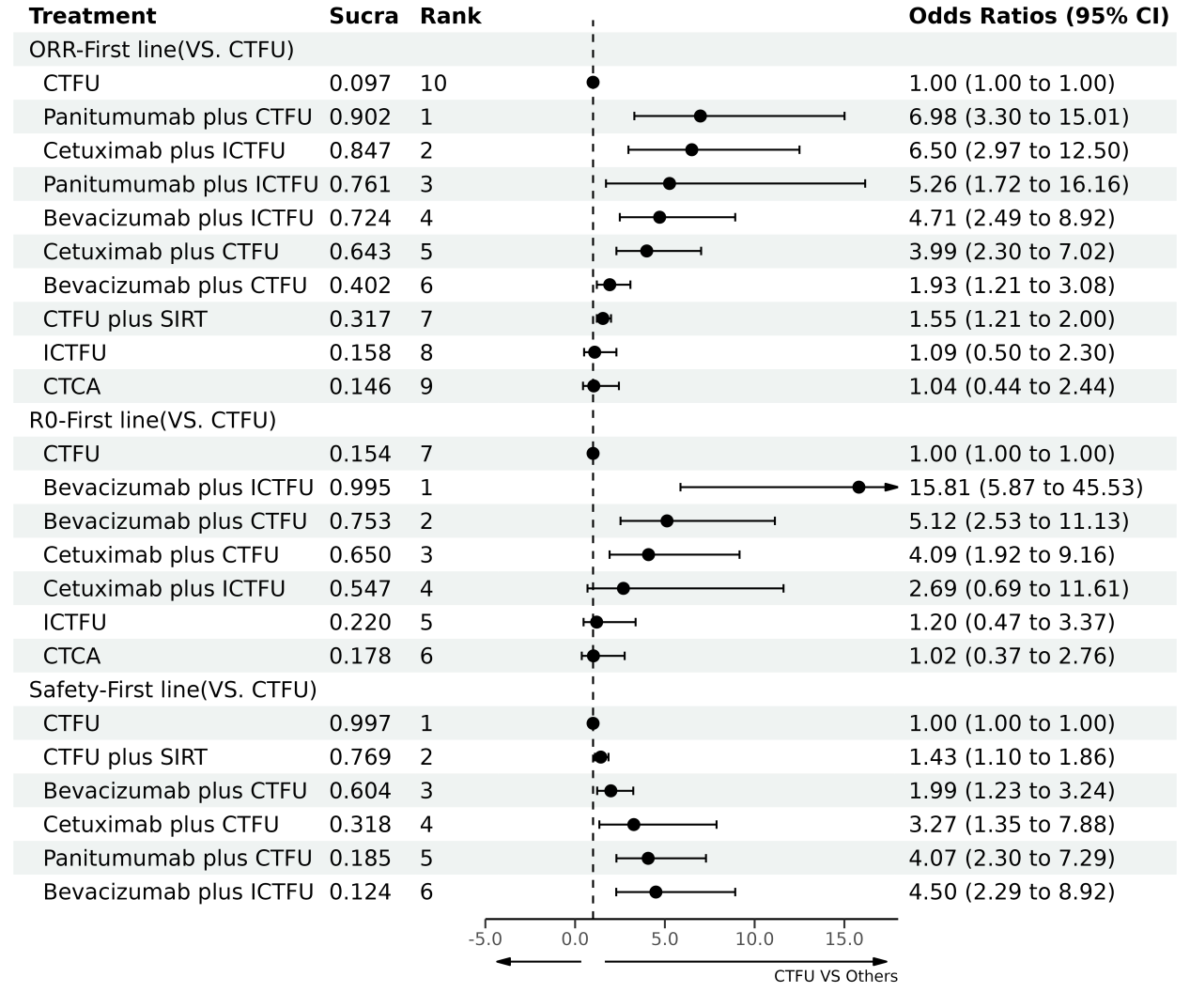


Abbreviation: CTFU, fluorouracil-based combination chemotherapy; CTCA, capecitabine-based combination chemotherapy; ICTFU, intensified fluorouracil-based combination chemotherapy; SIRT, Selective internal radiation therapy; SingleCT, single-drug chemotherapy

# Supplementary File 7. Additional results of subgroup analyses for first-line treatments

Table S4 League table for overall survival and progression-free survival of liver-limited metastatic patients

| Progression-free Survival | Overall Survival | | | | | | | | | | | | | | | |
| --- | --- | --- | --- | --- | --- | --- | --- | --- | --- | --- | --- | --- | --- | --- | --- | --- |
|  | ATE+BEV+ICTFU | N | N | N | N | N | N | N | N | N | N | N | N | N | N | N |
|  | 0.52 (0.27, 1.04) | BEV+CTFU | 0.88 (0.66, 1.18) | 0.66 (0.33, 1.34) | 0.74 (0.56, 0.99) | 0.7 (0.45, 1.09) | 1.02 (0.58, 1.8) | 1.29 (1.01, 1.64) | 1.29 (0.96, 1.73) | N | 0.94 (0.5, 1.79) | 0.83 (0.56, 1.24) | 0.83 (0.68, 1.02) | N | N | N |
|  | 0.72 (0.38, 1.37) | 1.37 (1.1, 1.71) | BEV+ICTFU | 0.75 (0.35, 1.6) | 0.84 (0.56, 1.26) | 0.79 (0.46, 1.34) | 1.15 (0.61, 2.17) | 1.46 (1, 2.13) | 1.46 (0.97, 2.2) | N | 1.06 (0.53, 2.15) | 0.94 (0.58, 1.54) | 0.94 (0.67, 1.34) | N | N | N |
|  | 0.27 (0.02, 3.92) | 0.51 (0.04, 6.85) | 0.37 (0.03, 5.04) | BEV+SCT | 1.12 (0.52, 2.4) | 1.05 (0.46, 2.42) | 1.54 (0.62, 3.78) | 1.95 (0.92, 4.1) | 1.95 (0.91, 4.18) | N | 1.42 (0.55, 3.68) | 1.26 (0.56, 2.82) | 1.26 (0.61, 2.62) | N | N | N |
|  | 0.56 (0.27, 1.16) | 1.07 (0.83, 1.38) | 0.78 (0.56, 1.09) | 2.08 (0.15, 27.98) | CET+CTFU | 0.94 (0.67, 1.32) | 1.37 (0.75, 2.52) | 1.74 (1.26, 2.4) | 1.74 (1.21, 2.5) | N | 1.27 (0.65, 2.5) | 1.12 (0.71, 1.76) | 1.13 (0.8, 1.58) | N | N | N |
|  | 0.54 (0.25, 1.17) | 1.03 (0.7, 1.5) | 0.75 (0.48, 1.16) | 2 (0.15, 27.33) | 0.96 (0.73, 1.27) | CET+ICTFU | 1.46 (0.73, 2.93) | 1.85 (1.16, 2.95) | 1.85 (1.12, 3.04) | N | 1.35 (0.64, 2.88) | 1.19 (0.68, 2.1) | 1.2 (0.74, 1.93) | N | N | N |
|  | 0.29 (0.13, 0.67) | 0.55 (0.34, 0.9) | 0.4 (0.23, 0.69) | 1.08 (0.08, 14.92) | 0.52 (0.31, 0.85) | 0.54 (0.3, 0.95) | CTCA | 1.27 (0.76, 2.12) | 1.27 (0.74, 2.18) | N | 0.93 (0.42, 2.03) | 0.82 (0.45, 1.5) | 0.82 (0.46, 1.47) | N | N | N |
|  | 0.32 (0.16, 0.65) | 0.61 (0.5, 0.74) | 0.44 (0.33, 0.59) | 1.18 (0.09, 15.85) | 0.57 (0.46, 0.7) | 0.59 (0.42, 0.84) | 1.1 (0.7, 1.73) | CTFU | 1 (0.85, 1.18) | N | 0.73 (0.4, 1.33) | 0.65 (0.47, 0.88) | 0.65 (0.49, 0.85) | N | N | N |
|  | 0.35 (0.17, 0.75) | 0.67 (0.49, 0.92) | 0.49 (0.33, 0.72) | 1.31 (0.1, 17.85) | 0.63 (0.46, 0.87) | 0.66 (0.43, 1.01) | 1.22 (0.73, 2.04) | 1.11 (0.87, 1.43) | CTFU+SIRT | N | 0.73 (0.39, 1.36) | 0.65 (0.45, 0.92) | 0.65 (0.47, 0.9) | N | N | N |
|  | 0.48 (0.17, 1.39) | 0.92 (0.4, 2.09) | 0.67 (0.29, 1.57) | 1.78 (0.12, 26.87) | 0.86 (0.38, 1.96) | 0.89 (0.38, 2.14) | 1.66 (0.66, 4.16) | 1.51 (0.68, 3.36) | 1.36 (0.59, 3.13) | GOLFIG | N | N | N | N | N | N |
|  | 0.42 (0.19, 0.92) | 0.8 (0.54, 1.18) | 0.58 (0.37, 0.91) | 1.56 (0.11, 21.29) | 0.75 (0.5, 1.12) | 0.78 (0.48, 1.27) | 1.45 (0.82, 2.55) | 1.32 (0.94, 1.85) | 1.19 (0.78, 1.81) | 0.87 (0.37, 2.07) | ICTFU | 0.88 (0.45, 1.73) | 0.89 (0.46, 1.71) | N | N | N |
|  | 0.53 (0.25, 1.14) | 1.01 (0.72, 1.43) | 0.74 (0.49, 1.11) | 1.98 (0.14, 26.86) | 0.95 (0.67, 1.35) | 0.99 (0.63, 1.55) | 1.83 (1.08, 3.14) | 1.67 (1.25, 2.22) | 1.5 (1.03, 2.19) | 1.11 (0.47, 2.58) | 1.27 (0.81, 1.98) | R/A+CTFU | 1 (0.66, 1.53) | N | N | N |
|  | 0.56 (0.29, 1.12) | 1.07 (0.97, 1.19) | 0.78 (0.62, 1) | 2.09 (0.16, 27.96) | 1.01 (0.77, 1.31) | 1.05 (0.71, 1.54) | 1.94 (1.19, 3.19) | 1.77 (1.44, 2.17) | 1.59 (1.15, 2.19) | 1.17 (0.51, 2.66) | 1.34 (0.9, 2) | 1.06 (0.75, 1.51) | PAN+CTFU | N | N | N |
|  | 0.6 (0.27, 1.31) | 1.14 (0.77, 1.7) | 0.83 (0.53, 1.31) | 2.23 (0.16, 30.67) | 1.07 (0.67, 1.7) | 1.11 (0.65, 1.92) | 2.07 (1.11, 3.86) | 1.88 (1.22, 2.9) | 1.69 (1.03, 2.79) | 1.25 (0.5, 3.09) | 1.43 (0.82, 2.48) | 1.13 (0.67, 1.9) | 1.06 (0.73, 1.56) | PAN+ICTFU | N | N |
|  | 0.15 (0.01, 2.19) | 0.28 (0.02, 3.83) | 0.2 (0.01, 2.81) | 0.54 (0.35, 0.83) | 0.26 (0.02, 3.65) | 0.27 (0.02, 3.86) | 0.5 (0.04, 7.28) | 0.46 (0.03, 6.39) | 0.41 (0.03, 5.79) | 0.3 (0.02, 4.71) | 0.35 (0.02, 4.92) | 0.27 (0.02, 3.92) | 0.26 (0.02, 3.57) | 0.24 (0.02, 3.44) | SCT | N |
|  | 0.11 (0, 3.18) | 0.21 (0.01, 5.62) | 0.15 (0.01, 4.15) | 0.41 (0.05, 3.15) | 0.2 (0.01, 5.33) | 0.2 (0.01, 5.62) | 0.38 (0.01, 10.57) | 0.35 (0.01, 9.31) | 0.31 (0.01, 8.5) | 0.23 (0.01, 6.87) | 0.26 (0.01, 7.2) | 0.21 (0.01, 5.67) | 0.19 (0.01, 5.24) | 0.18 (0.01, 5.04) | 0.75 (0.09, 6.12) | TAS102+BEV |

Abbreviation: ATE, Atezolizumab; BEV, Bevacizumab; BSC, best supportive care; CET, Cetuximab; CTFU, fluorouracil-based combination chemotherapy; CTCA, capecitabine-based combination chemotherapy; ICTFU, intensified fluorouracil-based combination chemotherapy; N, not available; PAN, panitumuma; SCT, single-drug chemotherapy.

Table S5 League table for overall survival and progression-free survival of liver-limited metastatic patients

| Overall Survival | | | | | | | | | | | | | | | | |
| --- | --- | --- | --- | --- | --- | --- | --- | --- | --- | --- | --- | --- | --- | --- | --- | --- |
| Progression-free Survival | BEV+CTCA | 0.65 (0.47, 0.9) | 1 (0.64, 1.54) | 0.77 (0.58, 1.02) | 0.81 (0.4, 1.65) | 1.32 (1.07, 1.64) | 1.42 (1.04, 1.93) | N | 0.88 (0.44, 1.76) | 0.85 (0.71, 1.02) | N | 1.23 (0.93, 1.64) | 1.42 (1.05, 1.91) | 2.49 (1.31, 4.75) | N | N |
|  | 1.22 (0.57, 2.62) | BEV+CTFU | 1.53 (0.89, 2.64) | 1.18 (0.77, 1.82) | 1.25 (0.66, 2.35) | 2.04 (1.38, 3) | 2.18 (1.39, 3.41) | N | 1.35 (0.63, 2.9) | 1.31 (0.9, 1.9) | N | 1.9 (1.23, 2.92) | 2.18 (1.4, 3.39) | 3.83 (1.86, 7.87) | N | N |
|  | 2.31 (0.99, 5.4) | 1.89 (1.3, 2.74) | BEV+SCT | 0.77 (0.46, 1.3) | 0.81 (0.35, 1.88) | 1.33 (0.82, 2.16) | 1.42 (0.83, 2.42) | N | 0.88 (0.39, 2) | 0.85 (0.53, 1.37) | N | 1.24 (0.74, 2.08) | 1.42 (0.84, 2.41) | 2.5 (1.15, 5.44) | N | N |
|  | 0.87 (0.38, 2.03) | 0.72 (0.5, 1.03) | 0.38 (0.23, 0.63) | CET+CTFU | 1.05 (0.49, 2.26) | 1.72 (1.21, 2.44) | 1.84 (1.22, 2.79) | N | 1.14 (0.54, 2.41) | 1.1 (0.79, 1.55) | N | 1.6 (1.07, 2.39) | 1.84 (1.22, 2.77) | 3.24 (1.6, 6.54) | N | N |
|  | N | N | N | N | CET+ICTFU | 1.63 (0.78, 3.42) | 1.74 (0.8, 3.79) | N | 1.08 (0.4, 2.93) | 1.05 (0.5, 2.18) | N | 1.52 (0.71, 3.27) | 1.74 (0.81, 3.77) | 3.06 (1.17, 8.02) | N | N |
|  | 0.74 (0.34, 1.62) | 0.6 (0.51, 0.72) | 0.32 (0.21, 0.48) | 0.84 (0.62, 1.16) | N | CTFU | 1.07 (0.86, 1.34) | N | 0.67 (0.32, 1.37) | 0.64 (0.53, 0.78) | N | 0.93 (0.65, 1.33) | 1.07 (0.86, 1.32) | 1.88 (1.02, 3.47) | N | N |
|  | 0.75 (0.33, 1.74) | 0.62 (0.44, 0.86) | 0.33 (0.2, 0.54) | 0.86 (0.56, 1.32) | N | 1.02 (0.76, 1.36) | CTFU+SIRT | N | 0.62 (0.29, 1.33) | 0.6 (0.45, 0.81) | N | 0.87 (0.57, 1.32) | 1 (0.73, 1.36) | 1.76 (0.92, 3.38) | N | N |
|  | 1.92 (0.87, 4.26) | 1.57 (1.24, 1.99) | 0.83 (0.53, 1.29) | 2.19 (1.55, 3.12) | N | 2.6 (2.22, 3.04) | 2.55 (1.83, 3.54) | GOLFIG | N | N | N | N | N | N | N | N |
|  | N | N | N | N | N | N | N | N | R/A+CTFU | 0.96 (0.47, 1.98) | N | 1.4 (0.66, 2.98) | 1.61 (0.75, 3.43) | 2.83 (1.1, 7.31) | N | N |
|  | 1.23 (0.57, 2.7) | 1.01 (0.85, 1.2) | 0.53 (0.35, 0.81) | 1.41 (1.03, 1.94) | N | 1.67 (1.58, 1.76) | 1.64 (1.22, 2.2) | 0.64 (0.54, 0.76) | N | PAN+CTFU | N | 1.45 (1.03, 2.04) | 1.67 (1.25, 2.22) | 2.93 (1.55, 5.57) | N | N |
|  | 1.37 (0.59, 3.16) | 1.12 (0.79, 1.59) | 0.59 (0.36, 0.99) | 1.57 (1.01, 2.42) | N | 1.86 (1.37, 2.52) | 1.82 (1.2, 2.77) | 0.71 (0.51, 1.01) | N | 1.11 (0.82, 1.5) | PAN+ICTFU | N | N | N | N | N |
|  | 1.28 (0.98, 1.67) | 1.05 (0.51, 2.14) | 0.56 (0.25, 1.25) | 1.46 (0.66, 3.26) | N | 1.74 (0.83, 3.62) | 1.7 (0.77, 3.75) | 0.67 (0.32, 1.42) | N | 1.04 (0.5, 2.16) | 0.94 (0.42, 2.07) | S1+BEV+SCT | 1.15 (0.76, 1.74) | 2.02 (1, 4.08) | N | N |
|  | N | N | N | N | N | N | N | N | N | N | N | N | SCT | 1.76 (0.92, 3.36) | N | N |
|  | 0.77 (0.16, 3.76) | 0.63 (0.16, 2.52) | 0.33 (0.08, 1.4) | 0.88 (0.22, 3.61) | N | 1.04 (0.26, 4.13) | 1.02 (0.25, 4.16) | 0.4 (0.1, 1.6) | N | 0.62 (0.16, 2.48) | 0.56 (0.14, 2.29) | 0.6 (0.13, 2.88) | N | SOR+CTFU | N | N |
|  | 2.51 (1, 6.31) | 2.05 (1.23, 3.45) | 1.09 (0.76, 1.56) | 2.87 (1.53, 5.4) | N | 3.4 (1.97, 5.89) | 3.33 (1.8, 6.19) | 1.31 (0.74, 2.32) | N | 2.03 (1.18, 3.52) | 1.83 (0.99, 3.42) | 1.96 (0.81, 4.73) | N | 3.27 (0.75, 14.33) | TAS102+BEV | N |
|  | 1.31 (0.56, 3.12) | 1.08 (0.72, 1.61) | 0.57 (0.33, 0.99) | 1.5 (0.88, 2.58) | N | 1.78 (1.15, 2.76) | 1.74 (1.03, 2.95) | 0.68 (0.43, 1.09) | N | 1.07 (0.69, 1.65) | 0.96 (0.56, 1.63) | 1.02 (0.45, 2.33) | N | 1.71 (0.4, 7.22) | 0.52 (0.27, 1.01) | TIV+CTFU |

Abbreviation: BEV, Bevacizumab; BSC, best supportive care; CED, Cediranib; CET, Cetuximab; CTFU, fluorouracil-based combination chemotherapy; CTCA, capecitabine-based combination chemotherapy; HAI, hepatic artery infusion; ICTFU, intensified fluorouracil-based combination chemotherapy; N, not available; PAN, panitumuma; R/A, resection or ablation; SIRT, Selective internal radiation therapy; SCT, single-drug chemotherapy; SOR, sorafenib; TIV, tivozanib.

# Supplementary File 8. Additional results of sensitive analyses

Table S6 League table for overall survival and progression-free survival of wild-type RAS/RAF patients

| Progression-free Survival | Overall Survival | | | | | |
| --- | --- | --- | --- | --- | --- | --- |
|  | BEV+CTFU | N | 0.69 (0.5, 0.95) | 1.14 (0.88, 1.47) | 0.84 (0.7, 1.02) | N |
|  | 1.11 (0.76, 1.62) | BEV+ICTFU | N | N | N | N |
|  | 1.5 (1, 2.26) | 1.35 (0.77, 2.37) | CET+CTFU | 1.65 (1.17, 2.33) | 1.23 (0.87, 1.72) | N |
|  | 0.79 (0.61, 1.03) | 0.71 (0.45, 1.14) | 0.53 (0.39, 0.72) | CTFU | 0.74 (0.61, 0.91) | N |
|  | 1 (0.82, 1.21) | 0.9 (0.59, 1.38) | 0.67 (0.46, 0.96) | 1.26 (1.05, 1.52) | PAN+CTFU | N |
|  | 1.06 (0.69, 1.63) | 0.96 (0.54, 1.7) | 0.71 (0.42, 1.2) | 1.35 (0.88, 2.06) | 1.06 (0.73, 1.56) | PAN+ICTFU |

Abbreviation: BEV, Bevacizumab; CET, Cetuximab; CTFU, fluorouracil-based combination chemotherapy; CTCA, capecitabine-based combination chemotherapy; ICTFU, intensified fluorouracil-based combination chemotherapy; N, not available; PAN, panitumuma.

Table S7 League table for overall survival and progression-free survival of wild-type patients

| Progression-free Survival | Overall Survival | | | | | | | | | | | | | | | | | | | | | | | | | |
| --- | --- | --- | --- | --- | --- | --- | --- | --- | --- | --- | --- | --- | --- | --- | --- | --- | --- | --- | --- | --- | --- | --- | --- | --- | --- | --- |
|  | ATE+BEV+ICTFU | N | N | N | N | N | N | N | N | N | N | N | N | N | N | N | N | N | N | N | N | N | N | N | N | N |
|  | 0.44 (0.21, 0.94) | BEV+CTCA | N | N | N | N | N | N | N | N | N | N | N | N | N | N | N | N | N | N | N | N | N | N | N | N |
|  | 0.52 (0.27, 1.03) | 1.19 (0.85, 1.66) | BEV+CTFU | 0.77 (0.62, 0.96) | 0.66 (0.33, 1.34) | 3.8 (1.67, 8.65) | N | 0.83 (0.44, 1.56) | 0.77 (0.38, 1.6) | 1.18 (0.66, 2.1) | 1.49 (1.14, 1.95) | 1.55 (1.15, 2.09) | 2.4 (1.52, 3.78) | N | 1.09 (0.57, 2.09) | 0.95 (0.66, 1.37) | 0.67 (0.41, 1.09) | N | 0.69 (0.3, 1.59) | 1.08 (0.83, 1.39) | 1.05 (0.55, 2) | 1.6 (1.14, 2.25) | 1.58 (0.99, 2.54) | N | N | N |
|  | 0.72 (0.38, 1.37) | 1.63 (1.1, 2.44) | 1.37 (1.1, 1.71) | BEV+ICTFU | 0.86 (0.41, 1.79) | 4.92 (2.1, 11.51) | N | 1.07 (0.55, 2.09) | 1 (0.47, 2.13) | 1.52 (0.82, 2.83) | 1.93 (1.37, 2.73) | 2 (1.39, 2.9) | 3.1 (1.88, 5.12) | N | 1.41 (0.71, 2.8) | 1.23 (0.8, 1.87) | 0.87 (0.51, 1.48) | N | 0.89 (0.38, 2.11) | 1.39 (1, 1.95) | 1.36 (0.69, 2.68) | 2.07 (1.38, 3.1) | 2.05 (1.22, 3.45) | N | N | N |
|  | 0.68 (0.25, 1.83) | 1.54 (0.69, 3.43) | 1.29 (0.62, 2.67) | 0.94 (0.44, 2.02) | BEV+SCT | 5.75 (1.94, 17.07) | N | 1.25 (0.48, 3.24) | 1.17 (0.43, 3.23) | 1.78 (0.71, 4.46) | 2.26 (1.06, 4.83) | 2.34 (1.09, 5.07) | 3.63 (1.57, 8.4) | N | 1.65 (0.63, 4.32) | 1.44 (0.65, 3.19) | 1.02 (0.43, 2.41) | N | 1.04 (0.35, 3.11) | 1.63 (0.77, 3.46) | 1.59 (0.61, 4.15) | 2.42 (1.1, 5.32) | 2.39 (1.02, 5.63) | N | N | N |
|  | N | N | N | N | N | BSC | N | 0.22 (0.08, 0.61) | 0.2 (0.07, 0.61) | 0.31 (0.12, 0.79) | 0.39 (0.18, 0.85) | 0.41 (0.18, 0.9) | 0.63 (0.32, 1.25) | N | 0.29 (0.11, 0.77) | 0.25 (0.11, 0.57) | 0.18 (0.08, 0.4) | N | 0.18 (0.06, 0.55) | 0.28 (0.12, 0.67) | 0.28 (0.1, 0.73) | 0.42 (0.2, 0.89) | 0.42 (0.17, 0.99) | N | N | N |
|  | 0.3 (0.12, 0.73) | 0.67 (0.34, 1.34) | 0.57 (0.31, 1.03) | 0.41 (0.22, 0.78) | 0.44 (0.17, 1.12) | N | CED+CTCA | N | N | N | N | N | N | N | N | N | N | N | N | N | N | N | N | N | N | N |
|  | 0.55 (0.26, 1.16) | 1.24 (0.78, 1.97) | 1.04 (0.76, 1.43) | 0.76 (0.52, 1.12) | 0.81 (0.37, 1.79) | N | 1.85 (1.01, 3.38) | CET+CTFU | 0.94 (0.67, 1.32) | 1.43 (0.6, 3.38) | 1.81 (0.91, 3.6) | 1.87 (0.93, 3.79) | 2.9 (1.33, 6.35) | N | 1.32 (0.53, 3.27) | 1.15 (0.55, 2.39) | 0.81 (0.36, 1.81) | N | 0.84 (0.29, 2.39) | 1.31 (0.66, 2.59) | 1.27 (0.51, 3.14) | 1.93 (0.94, 3.98) | 1.92 (0.87, 4.23) | N | N | N |
|  | 0.53 (0.24, 1.17) | 1.2 (0.7, 2.05) | 1 (0.66, 1.53) | 0.73 (0.45, 1.18) | 0.78 (0.33, 1.81) | N | 1.78 (0.91, 3.44) | 0.96 (0.73, 1.27) | CET+ICTFU | 1.52 (0.6, 3.82) | 1.93 (0.89, 4.14) | 2 (0.91, 4.35) | 3.09 (1.31, 7.24) | N | 1.41 (0.53, 3.71) | 1.22 (0.55, 2.74) | 0.87 (0.36, 2.07) | N | 0.89 (0.3, 2.67) | 1.39 (0.65, 2.99) | 1.36 (0.51, 3.55) | 2.06 (0.93, 4.57) | 2.04 (0.86, 4.82) | N | N | N |
|  | 0.31 (0.13, 0.72) | 0.69 (0.37, 1.3) | 0.58 (0.34, 0.99) | 0.42 (0.24, 0.75) | 0.45 (0.18, 1.11) | N | 1.03 (0.78, 1.37) | 0.56 (0.33, 0.95) | 0.58 (0.32, 1.06) | CTCA | 1.27 (0.76, 2.12) | 1.31 (0.77, 2.23) | 2.03 (1.08, 3.83) | N | 0.92 (0.42, 2.03) | 0.8 (0.45, 1.46) | 0.57 (0.3, 1.1) | N | 0.59 (0.32, 1.06) | 0.91 (0.48, 1.72) | 0.89 (0.67, 1.18) | 1.36 (0.78, 2.37) | 1.34 (0.71, 2.56) | N | N | N |
|  | 0.34 (0.16, 0.7) | 0.76 (0.5, 1.17) | 0.64 (0.49, 0.84) | 0.47 (0.33, 0.66) | 0.5 (0.23, 1.08) | N | 1.13 (0.66, 1.93) | 0.61 (0.46, 0.81) | 0.64 (0.43, 0.95) | 1.1 (0.7, 1.73) | CTFU | 1.04 (0.9, 1.19) | 1.61 (1.11, 2.32) | N | 0.73 (0.4, 1.32) | 0.64 (0.48, 0.85) | 0.45 (0.3, 0.68) | N | 0.46 (0.21, 1.01) | 0.72 (0.5, 1.05) | 0.7 (0.39, 1.26) | 1.07 (0.87, 1.32) | 1.06 (0.72, 1.56) | N | N | N |
|  | 0.37 (0.18, 0.78) | 0.85 (0.54, 1.33) | 0.71 (0.53, 0.96) | 0.52 (0.36, 0.75) | 0.55 (0.25, 1.21) | N | 1.26 (0.73, 2.18) | 0.68 (0.5, 0.93) | 0.71 (0.47, 1.08) | 1.22 (0.76, 1.95) | 1.11 (0.98, 1.26) | CTFU+SIRT | 1.55 (1.05, 2.29) | N | 0.7 (0.38, 1.3) | 0.61 (0.45, 0.85) | 0.43 (0.28, 0.67) | N | 0.45 (0.2, 0.99) | 0.7 (0.47, 1.03) | 0.68 (0.37, 1.24) | 1.03 (0.8, 1.33) | 1.02 (0.68, 1.54) | N | N | N |
|  | N | N | N | N | N | N | N | N | N | N | N | N | HAI | N | 0.45 (0.23, 0.92) | 0.4 (0.25, 0.63) | 0.28 (0.18, 0.44) | N | 0.29 (0.12, 0.68) | 0.45 (0.27, 0.76) | 0.44 (0.22, 0.87) | 0.67 (0.49, 0.9) | 0.66 (0.39, 1.13) | N | N | N |
|  | 0.51 (0.17, 1.5) | 1.15 (0.47, 2.84) | 0.97 (0.42, 2.24) | 0.71 (0.3, 1.68) | 0.75 (0.25, 2.28) | N | 1.71 (0.66, 4.44) | 0.93 (0.4, 2.16) | 0.96 (0.4, 2.35) | 1.66 (0.66, 4.14) | 1.51 (0.68, 3.34) | 1.36 (0.61, 3.04) | N | GOLFIG | N | N | N | N | N | N | N | N | N | N | N | N |
|  | 0.44 (0.2, 0.99) | 1 (0.58, 1.74) | 0.84 (0.55, 1.3) | 0.61 (0.38, 1) | 0.65 (0.28, 1.53) | N | 1.49 (0.79, 2.8) | 0.81 (0.52, 1.26) | 0.84 (0.5, 1.42) | 1.45 (0.82, 2.55) | 1.32 (0.93, 1.85) | 1.19 (0.82, 1.71) | N | 0.87 (0.37, 2.08) | ICTFU | 0.87 (0.45, 1.68) | 0.62 (0.3, 1.26) | N | 0.63 (0.24, 1.69) | 0.99 (0.49, 1.99) | 0.96 (0.42, 2.21) | 1.47 (0.78, 2.75) | 1.45 (0.71, 2.95) | N | N | N |
|  | 0.56 (0.26, 1.23) | 1.27 (0.76, 2.14) | 1.07 (0.72, 1.58) | 0.78 (0.5, 1.22) | 0.83 (0.36, 1.89) | N | 1.89 (1.03, 3.47) | 1.02 (0.68, 1.53) | 1.06 (0.65, 1.74) | 1.83 (1.07, 3.14) | 1.67 (1.25, 2.22) | 1.5 (1.1, 2.06) | N | 1.11 (0.48, 2.57) | 1.27 (0.81, 1.98) | R/A+CTFU | 0.71 (0.43, 1.16) | N | 0.73 (0.31, 1.68) | 1.14 (0.73, 1.77) | 1.11 (0.57, 2.13) | 1.69 (1.17, 2.41) | 1.67 (1.03, 2.71) | N | N | N |
|  | 0.69 (0.22, 2.16) | 1.57 (0.59, 4.16) | 1.32 (0.53, 3.27) | 0.96 (0.38, 2.45) | 1.02 (0.59, 1.77) | N | 2.33 (0.78, 6.92) | 1.26 (0.48, 3.31) | 1.31 (0.48, 3.58) | 2.26 (0.79, 6.47) | 2.05 (0.8, 5.32) | 1.85 (0.71, 4.83) | N | 1.36 (0.39, 4.68) | 1.56 (0.57, 4.3) | 1.23 (0.46, 3.31) | R/A+SCT | N | 1.03 (0.42, 2.49) | 1.6 (0.92, 2.79) | 1.56 (0.76, 3.19) | 2.38 (1.68, 3.36) | 2.36 (1.34, 4.13) | N | N | N |
|  | 0.3 (0.14, 0.65) | 0.69 (0.43, 1.1) | 0.58 (0.42, 0.81) | 0.42 (0.29, 0.63) | 0.45 (0.2, 1) | N | 1.03 (0.52, 2.03) | 0.56 (0.35, 0.88) | 0.58 (0.34, 0.99) | 1 (0.54, 1.86) | 0.91 (0.59, 1.39) | 0.82 (0.52, 1.27) | N | 0.6 (0.24, 1.47) | 0.69 (0.4, 1.19) | 0.54 (0.32, 0.91) | 0.44 (0.17, 1.16) | PEL+BEV+CTFU | N | N | N | N | N | N | N | N |
|  | 0.58 (0.19, 1.83) | 1.32 (0.5, 3.51) | 1.11 (0.44, 2.78) | 0.81 (0.31, 2.08) | 0.86 (0.27, 2.77) | N | 1.96 (0.87, 4.38) | 1.06 (0.42, 2.68) | 1.1 (0.42, 2.89) | 1.9 (0.89, 4.04) | 1.73 (0.72, 4.16) | 1.56 (0.64, 3.78) | N | 1.15 (0.35, 3.74) | 1.32 (0.51, 3.37) | 1.04 (0.41, 2.61) | 0.84 (0.23, 3.05) | 1.91 (0.72, 5.06) | S1 | 1.56 (0.65, 3.73) | 1.52 (0.9, 2.56) | 2.31 (1.03, 5.26) | 2.29 (0.96, 5.52) | N | N | N |
|  | 0.57 (0.28, 1.17) | 1.3 (1.02, 1.65) | 1.09 (0.86, 1.38) | 0.79 (0.58, 1.1) | 0.84 (0.39, 1.81) | N | 1.93 (1.02, 3.66) | 1.04 (0.7, 1.55) | 1.09 (0.67, 1.77) | 1.87 (1.05, 3.33) | 1.7 (1.19, 2.44) | 1.53 (1.05, 2.24) | N | 1.13 (0.47, 2.69) | 1.29 (0.79, 2.12) | 1.02 (0.64, 1.61) | 0.83 (0.32, 2.12) | 1.87 (1.25, 2.81) | 0.98 (0.38, 2.54) | S1+BEV+SCT | 1.03 (0.51, 2.06) | 1.52 (0.82, 2.85) | 1.51 (0.75, 3.05) | N | N | N |
|  | 0.37 (0.15, 0.92) | 0.85 (0.43, 1.68) | 0.71 (0.39, 1.29) | 0.52 (0.27, 0.98) | 0.55 (0.22, 1.41) | N | 1.26 (0.85, 1.86) | 0.68 (0.37, 1.24) | 0.71 (0.37, 1.37) | 1.22 (0.92, 1.61) | 1.11 (0.65, 1.89) | 1 (0.58, 1.73) | N | 0.73 (0.28, 1.91) | 0.84 (0.45, 1.58) | 0.66 (0.36, 1.22) | 0.54 (0.18, 1.6) | 1.22 (0.62, 2.42) | 0.64 (0.32, 1.3) | 0.65 (0.34, 1.24) | S1+SCT | 1.48 (0.97, 2.27) | 1.47 (0.86, 2.51) | N | N | N |
|  | 0.37 (0.12, 1.09) | 0.83 (0.33, 2.07) | 0.7 (0.3, 1.62) | 0.51 (0.21, 1.22) | 0.54 (0.35, 0.83) | N | 1.23 (0.44, 3.46) | 0.67 (0.27, 1.65) | 0.69 (0.27, 1.79) | 1.2 (0.44, 3.24) | 1.09 (0.45, 2.64) | 0.98 (0.4, 2.41) | N | 0.72 (0.22, 2.36) | 0.83 (0.32, 2.15) | 0.65 (0.26, 1.65) | 0.53 (0.38, 0.75) | 1.2 (0.48, 2.98) | 0.63 (0.18, 2.19) | 0.64 (0.27, 1.54) | 0.98 (0.35, 2.74) | SCT | 0.99 (0.64, 1.54) | N | N | N |
|  | 0.39 (0.17, 0.88) | 0.89 (0.51, 1.56) | 0.74 (0.47, 1.17) | 0.54 (0.33, 0.9) | 0.58 (0.24, 1.36) | N | 1.32 (0.69, 2.51) | 0.71 (0.45, 1.13) | 0.74 (0.43, 1.27) | 1.28 (0.72, 2.28) | 1.16 (0.81, 1.67) | 1.05 (0.71, 1.54) | N | 0.77 (0.32, 1.85) | 0.88 (0.54, 1.45) | 0.7 (0.44, 1.1) | 0.57 (0.2, 1.56) | 1.28 (0.73, 2.24) | 0.67 (0.26, 1.74) | 0.68 (0.41, 1.14) | 1.05 (0.55, 2) | 1.07 (0.41, 2.78) | SOR+CTFU | N | N | N |
|  | 0.79 (0.29, 2.16) | 1.79 (0.79, 4.06) | 1.5 (0.71, 3.17) | 1.09 (0.5, 2.39) | 1.16 (0.97, 1.39) | N | 2.65 (1.02, 6.91) | 1.44 (0.64, 3.24) | 1.49 (0.63, 3.53) | 2.57 (1.03, 6.43) | 2.34 (1.06, 5.19) | 2.11 (0.95, 4.72) | N | 1.55 (0.5, 4.77) | 1.78 (0.75, 4.23) | 1.4 (0.6, 3.26) | 1.14 (0.64, 2.03) | 2.58 (1.14, 5.86) | 1.35 (0.42, 4.43) | 1.38 (0.63, 3.01) | 2.11 (0.81, 5.48) | 2.15 (1.35, 3.43) | 2.02 (0.84, 4.83) | TAS102+BEV | N | N |
|  | 0.56 (0.26, 1.24) | 1.28 (0.76, 2.16) | 1.08 (0.72, 1.61) | 0.78 (0.49, 1.24) | 0.83 (0.36, 1.91) | N | 1.9 (0.92, 3.9) | 1.03 (0.62, 1.72) | 1.07 (0.6, 1.92) | 1.85 (0.95, 3.58) | 1.68 (1.03, 2.72) | 1.51 (0.92, 2.5) | N | 1.11 (0.44, 2.82) | 1.27 (0.7, 2.31) | 1 (0.57, 1.76) | 0.82 (0.3, 2.21) | 1.85 (1.1, 3.11) | 0.97 (0.36, 2.65) | 0.99 (0.62, 1.57) | 1.51 (0.74, 3.11) | 1.54 (0.6, 3.92) | 1.44 (0.79, 2.64) | 0.72 (0.31, 1.67) | TIV+CTFU | N |
|  | 0.31 (0.1, 0.92) | 0.7 (0.28, 1.75) | 0.59 (0.25, 1.39) | 0.43 (0.18, 1.03) | 0.46 (0.15, 1.4) | N | 1.04 (0.51, 2.15) | 0.56 (0.24, 1.33) | 0.59 (0.24, 1.45) | 1.01 (0.52, 1.98) | 0.92 (0.41, 2.07) | 0.83 (0.37, 1.89) | N | 0.61 (0.2, 1.9) | 0.7 (0.29, 1.68) | 0.55 (0.23, 1.3) | 0.45 (0.13, 1.56) | 1.02 (0.41, 2.53) | 0.53 (0.21, 1.35) | 0.54 (0.22, 1.31) | 0.83 (0.45, 1.53) | 0.85 (0.25, 2.81) | 0.79 (0.33, 1.93) | 0.39 (0.13, 1.22) | 0.55 (0.21, 1.41) | TSU68+S1+SCT |

Abbreviation: ATE, Atezolizumab; BEV, Bevacizumab; BSC, best supportive care; CED, Cediranib; CET, Cetuximab; CTFU, fluorouracil-based combination chemotherapy; CTCA, capecitabine-based combination chemotherapy; HAI, hepatic artery infusion; ICTFU, intensified fluorouracil-based combination chemotherapy; N, not available; PAN, panitumuma; pelareorep, PAL; R/A, resection or ablation; SIRT, Selective internal radiation therapy; SCT, single-drug chemotherapy; SOR, sorafenib; TIV, tivozanib.

# Supplementary File 9. Additional results of maintenance treatment

| Trail^*^ | Treatment | | SAEs, % | |
| --- | --- | --- | --- | --- |
|  | T | C | T | C |
| Geng 2020[10] | Capecitabine | Observation | 32 | 21.7 |
| PRODIGE 9[18] | Bevacizumab | Observation | 80 | 79 |
| CAIRO3[21,22] | Capecitabine+Bevacizumab | Observation | 60 | 34 |

*: lack of safety data for patients with previously untreated unresectable colorectal liver metastases

Abbreviation: SAE, adverse events graded as 3 or above.

# Supplementary File 10. Heterogeneity assessment results

| Nework | Data points | Ratios | I^2 |
| --- | --- | --- | --- |
| OS for First-line Liver Metastases | 31 | 0.8238 | 0% |
| PFS for First-line Liver Metastases | 42 | 1.075 | 9% |
| OS for First-line Multiple-organ Metastases | 14 | 0.9358 | 1% |
| PFS for First-line Multiple-organ Metastases | 17 | 1.288 | 27% |
| OS for First-line Liver-limited Metastases | 16 | 0.8923 | 0% |
| PFS for First-line Liver-limited Metastases | 24 | 1.196 | 20% |
| OS for Maintenance Treatment | 6 | 1.756 | 53% |
| PFS for Maintenance Treatment | 6 | 1.78 | 53% |
| AE for Maintenance Treatment | 10 | 0.9324 | 3% |
| AE for First-line Treatment | 42 | 0.927 | 0% |
| ORR for First-line Treatment | 28 | 0.9609 | 0% |
| R0 resection rate for First-line Treatment | 16 | 0.8928 | 0% |
| OS for First-line Liver Metastases (RAS/RAF Wild-type) | 6 | 0.5835 | 0% |
| OS for First-line Liver Metastases (Wild-type Patients) | 24 | 0.9028 | 0% |
| PFS for First-line Liver Metastases (RAS/RAF Wild-type) | 8 | 0.8617 | 0% |
| PFS for First-line Liver Metastases (Wild-type Patients) | 32 | 1.052 | 8% |

# Supplementary File 11. Forest plots depicting results of head-to-head comparisons and node-splitting analysis of inconsistency

1. OS for First-line Liver Metastases


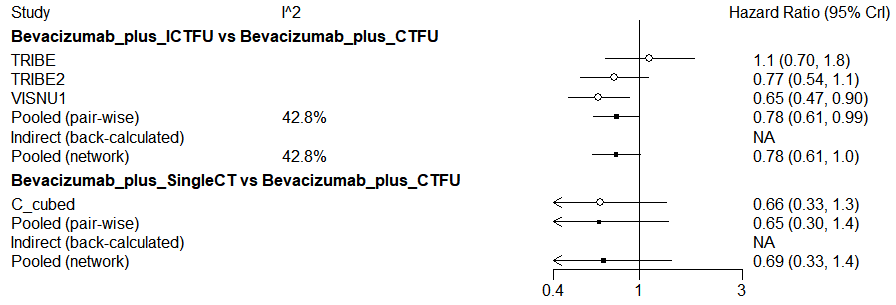


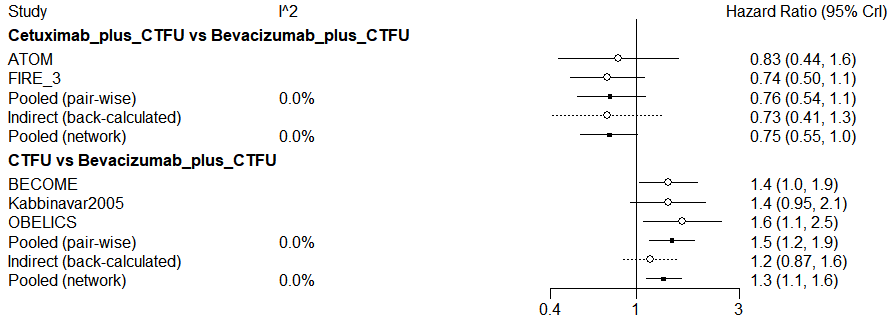


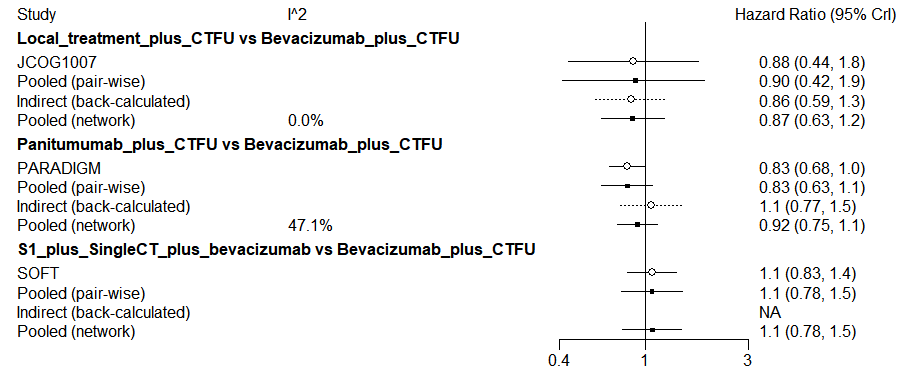


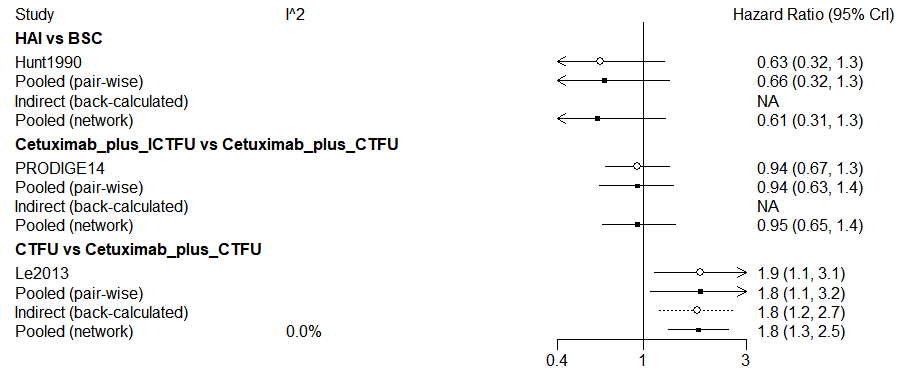


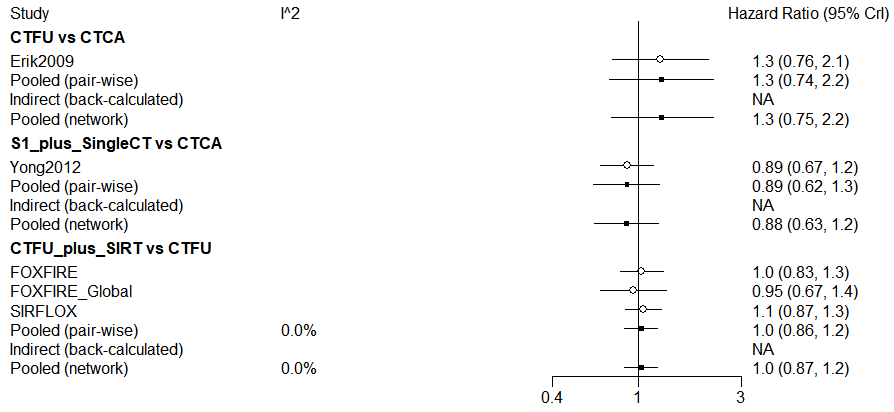


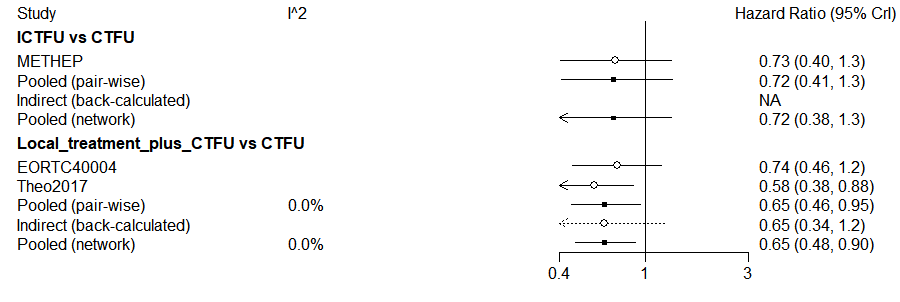


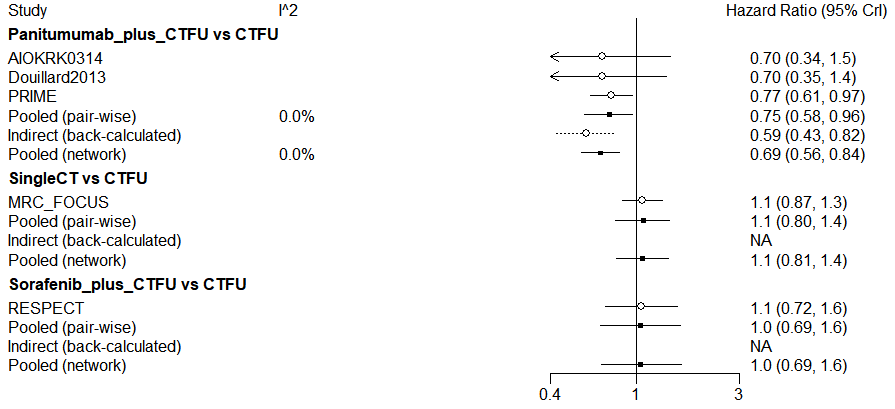


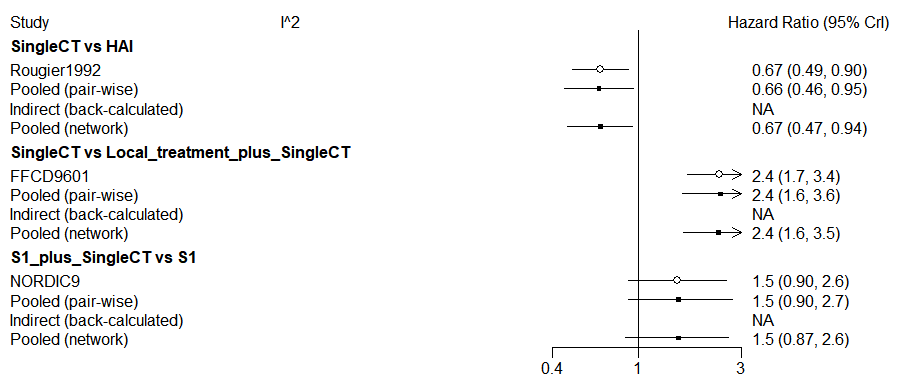


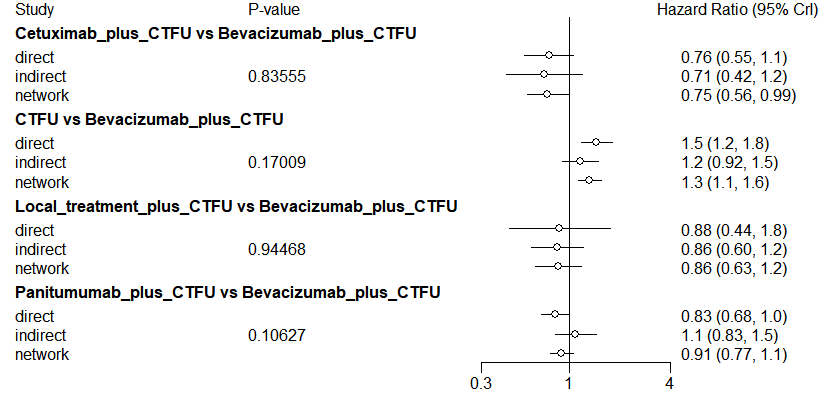


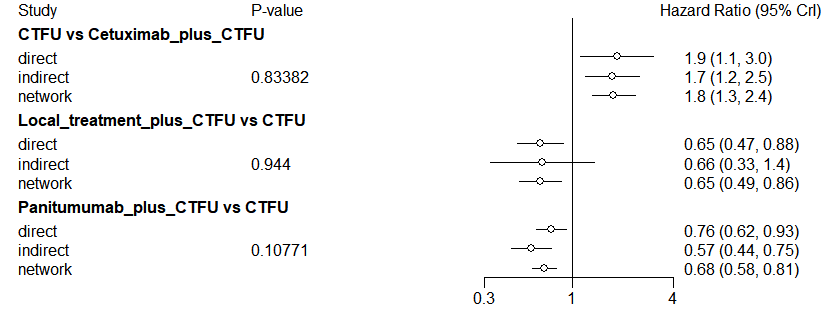


1. PFS for First-line Liver Metastases


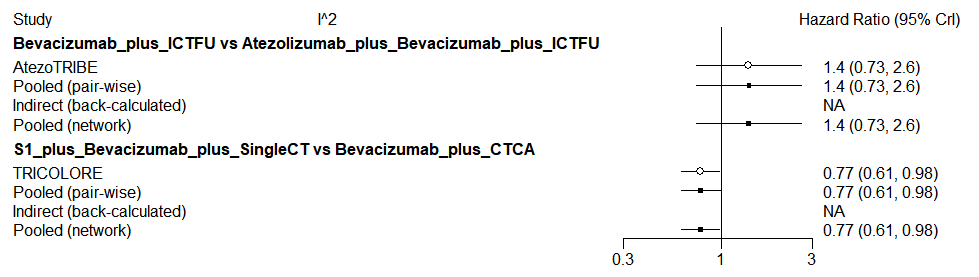


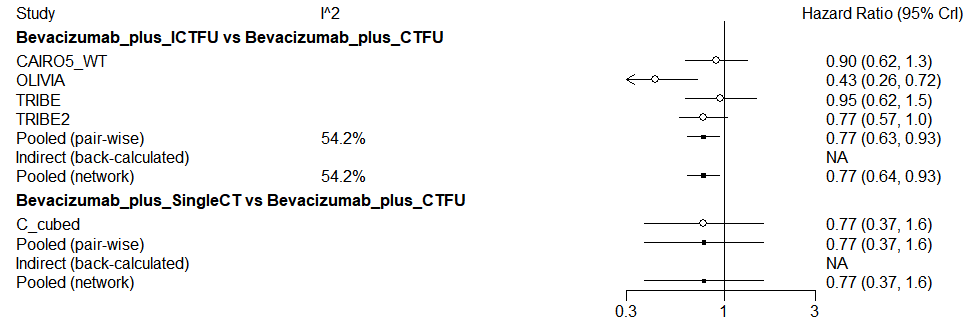


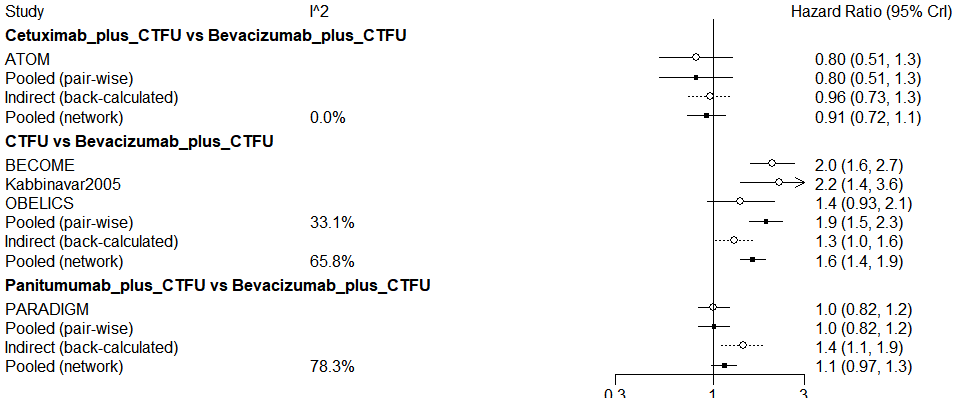


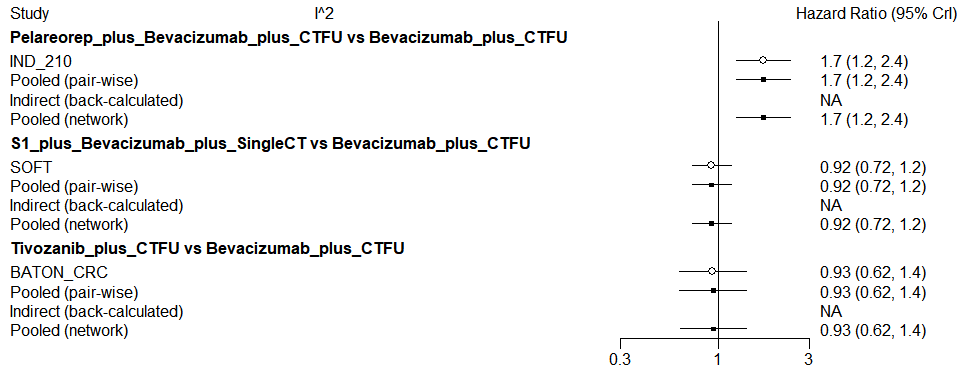


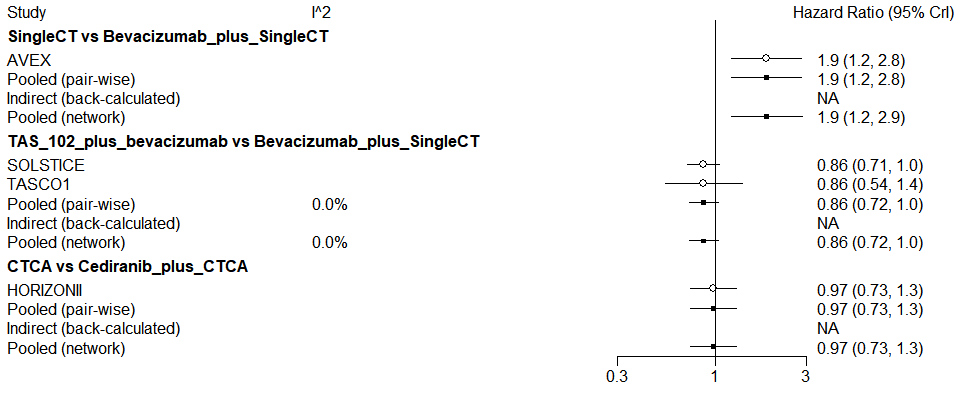


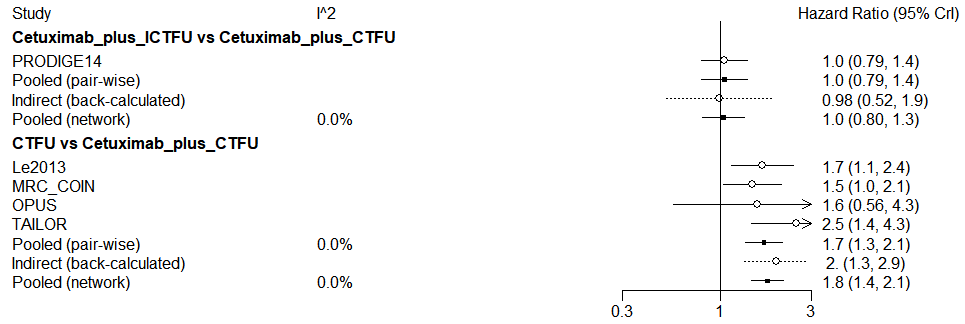


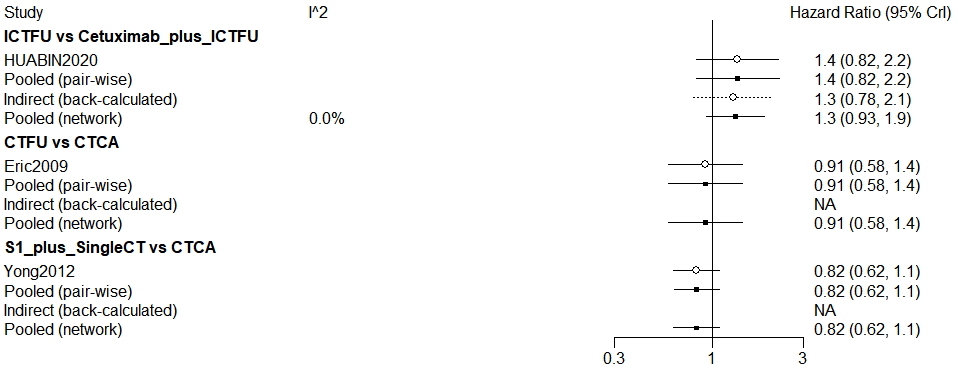


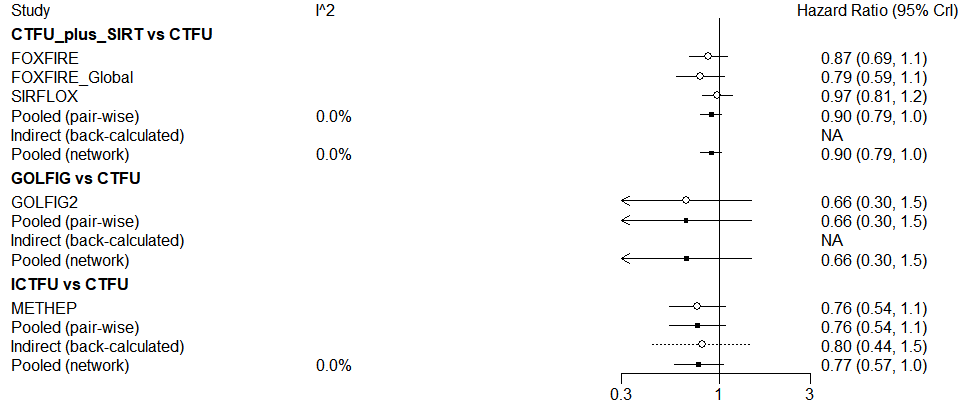


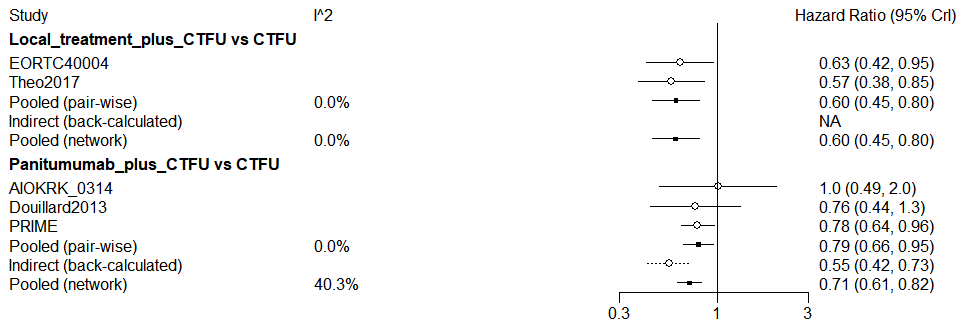


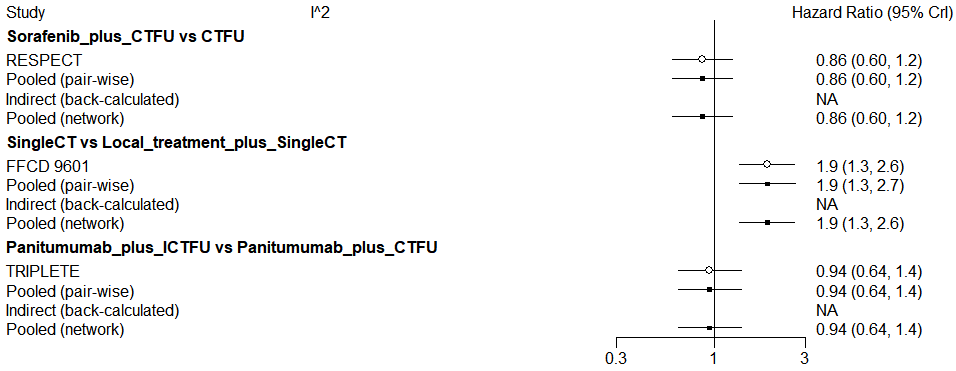


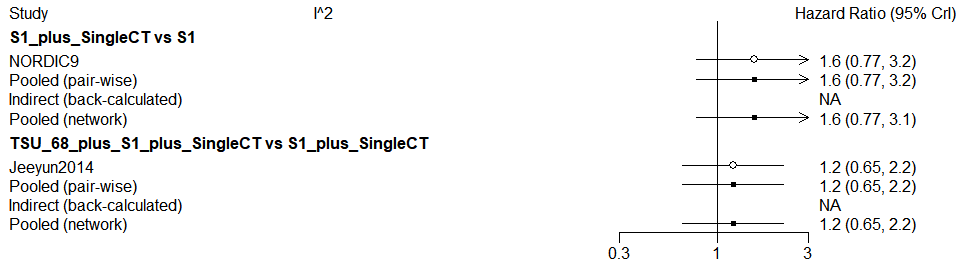


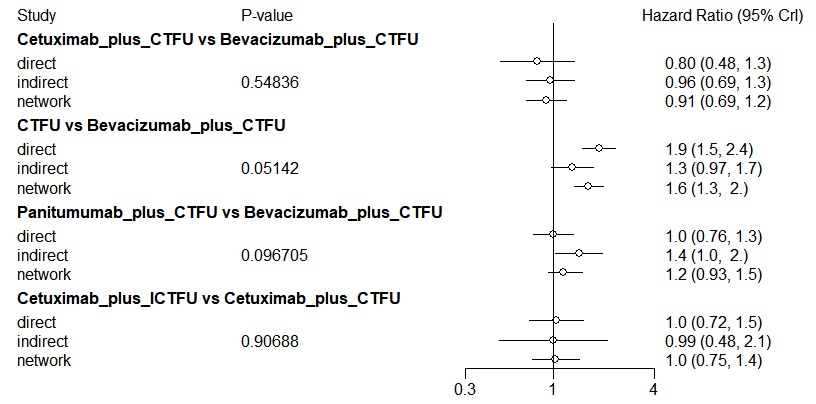


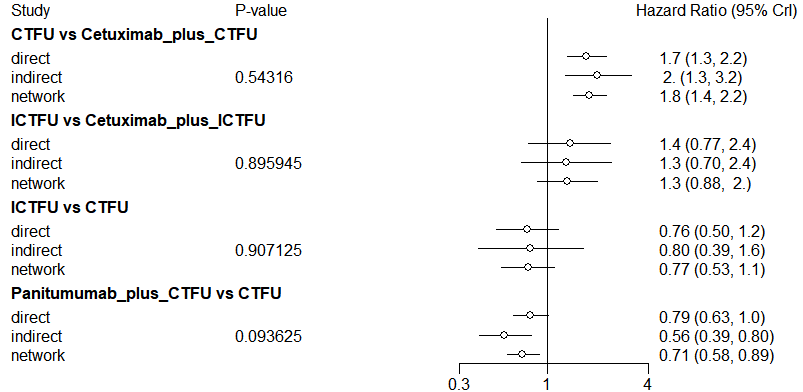


1. OS for First-line Multiple-organ Metastases


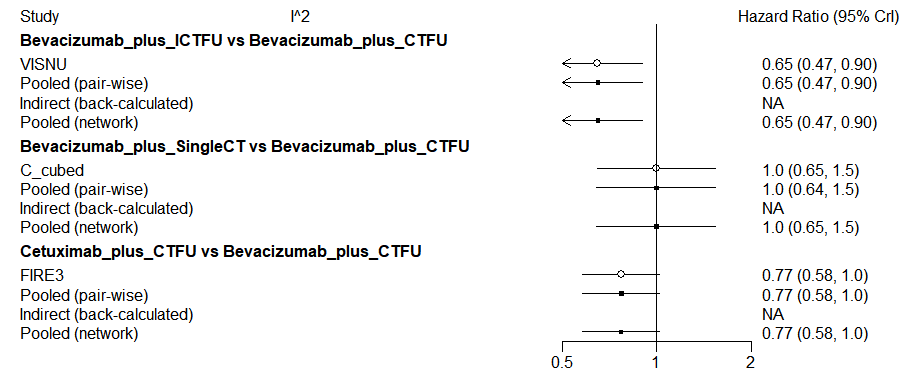


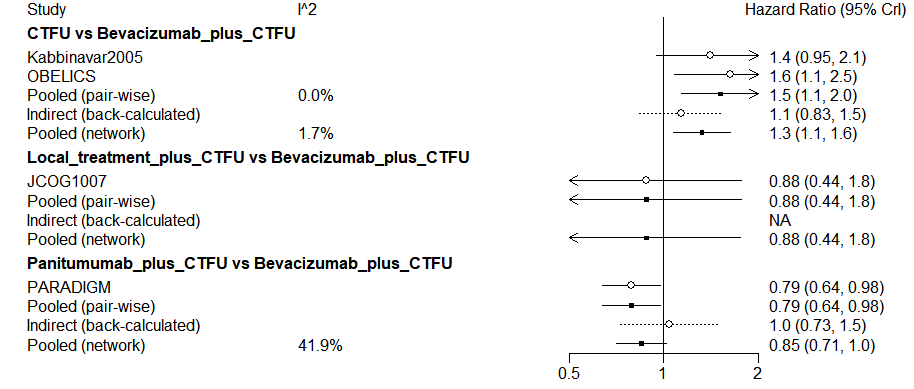


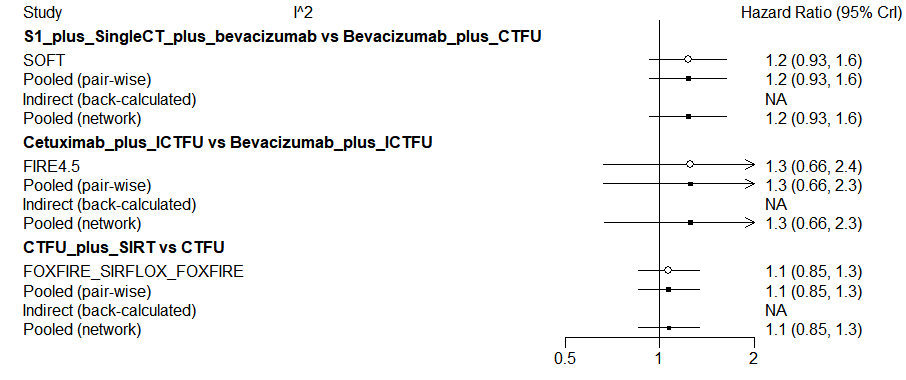


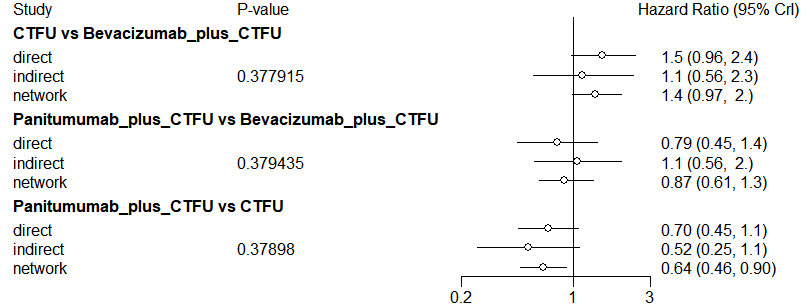


1. PFS for First-line Multiple-organ Metastases


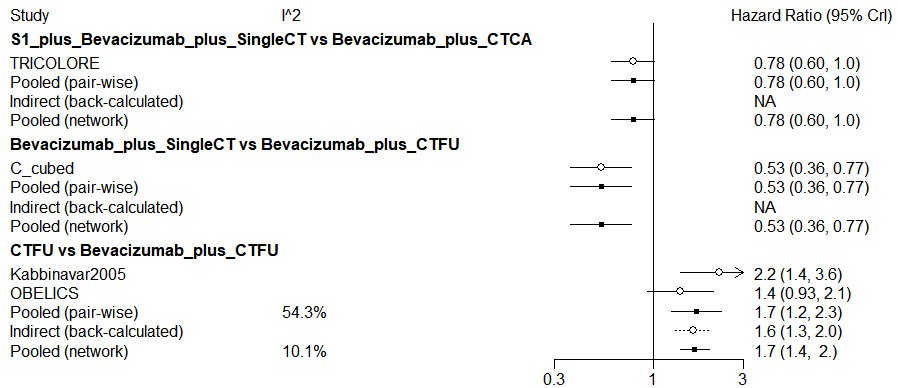


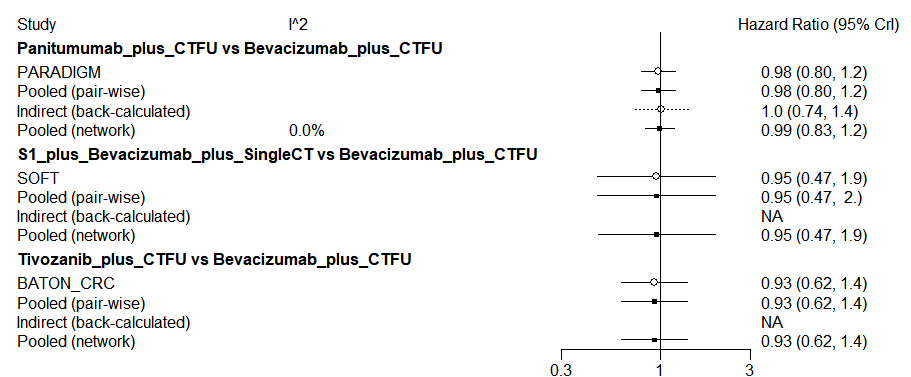


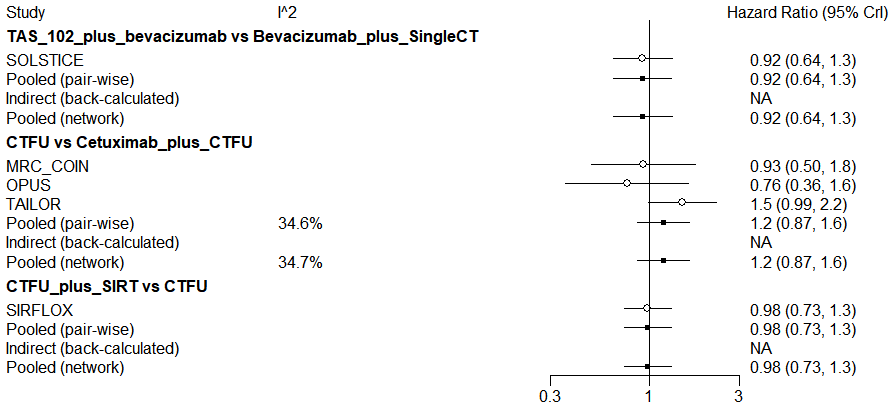


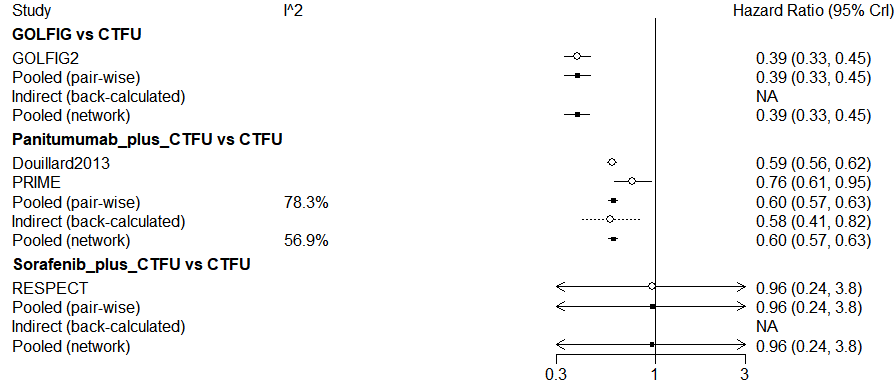


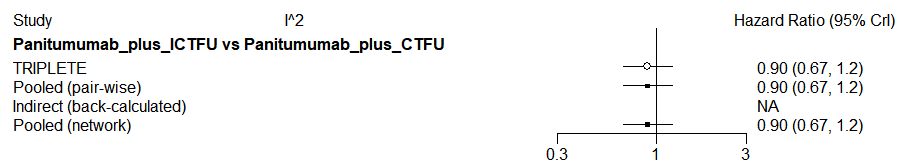


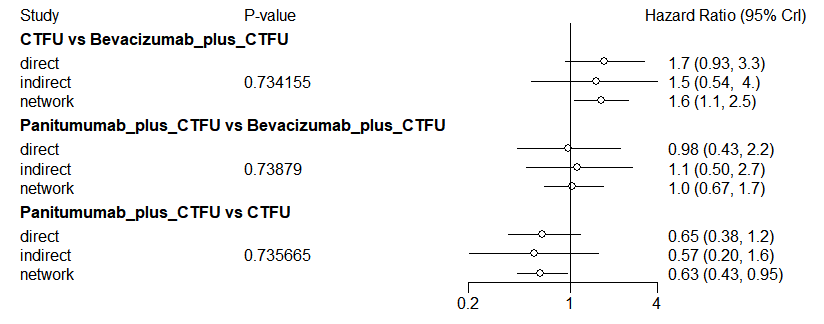


1. OS for First-line Liver-limited Metastases


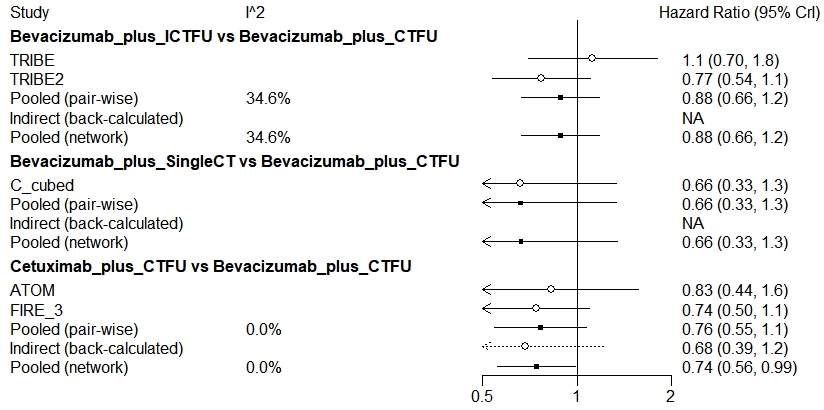


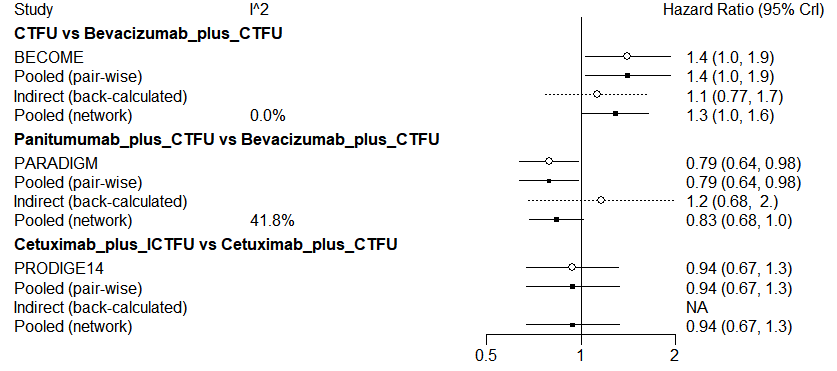


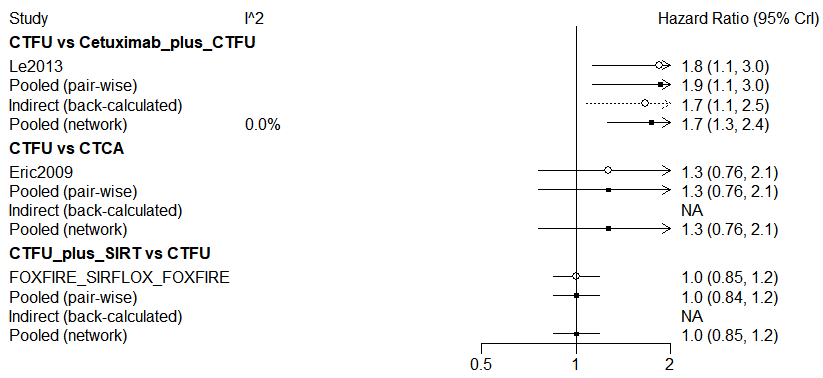


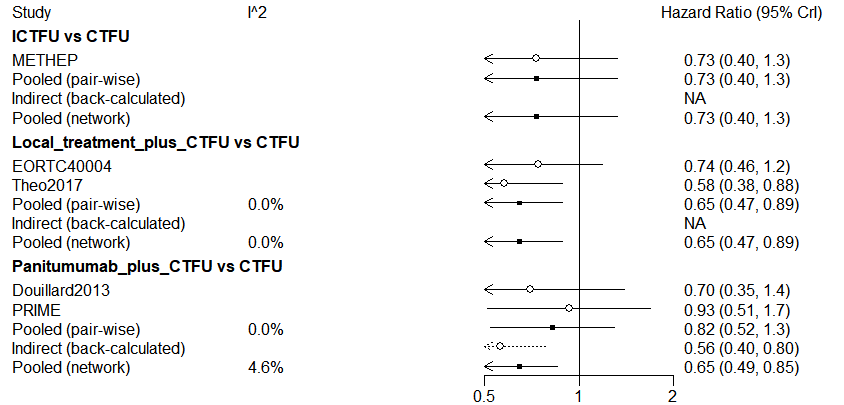


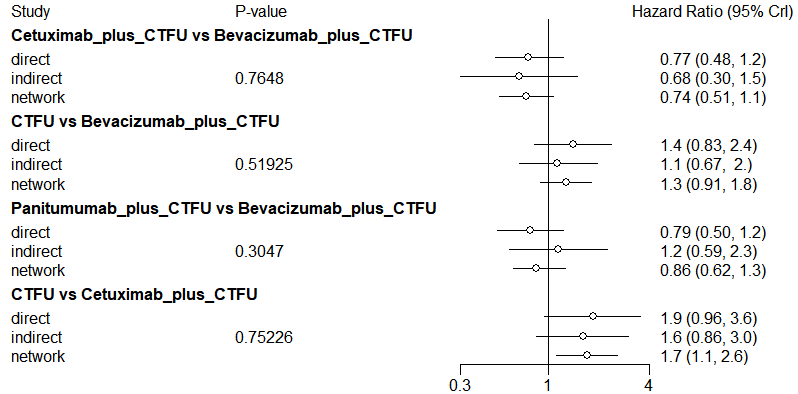


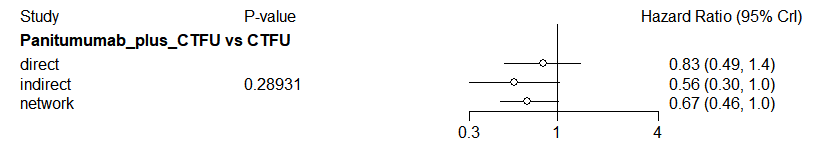


1. PFS for First-line Liver-limited Metastases


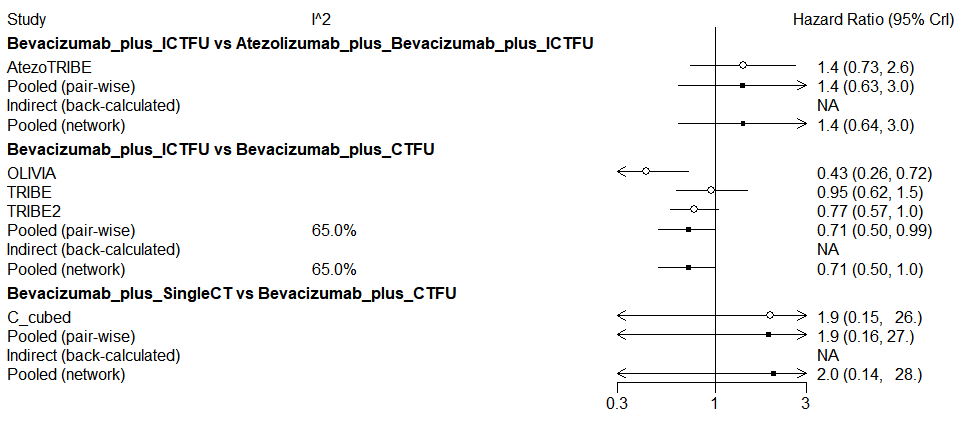


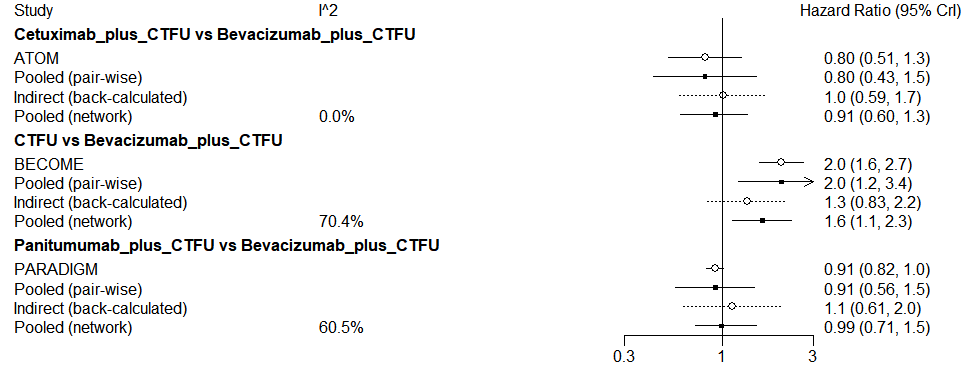


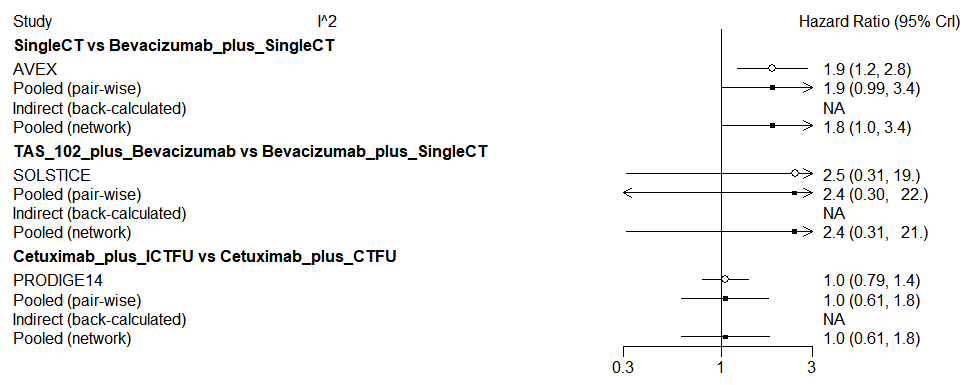


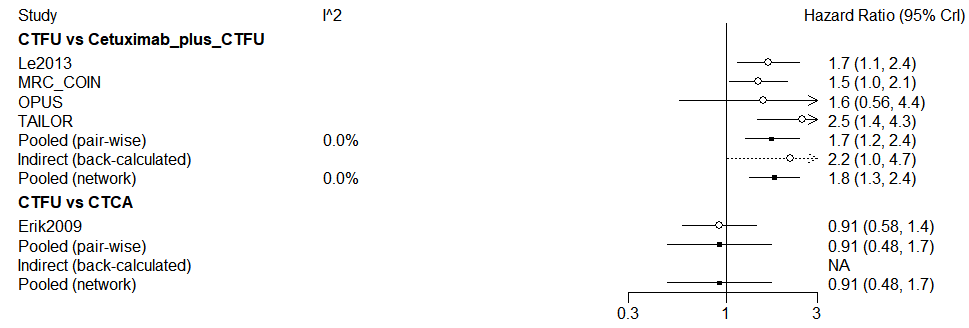


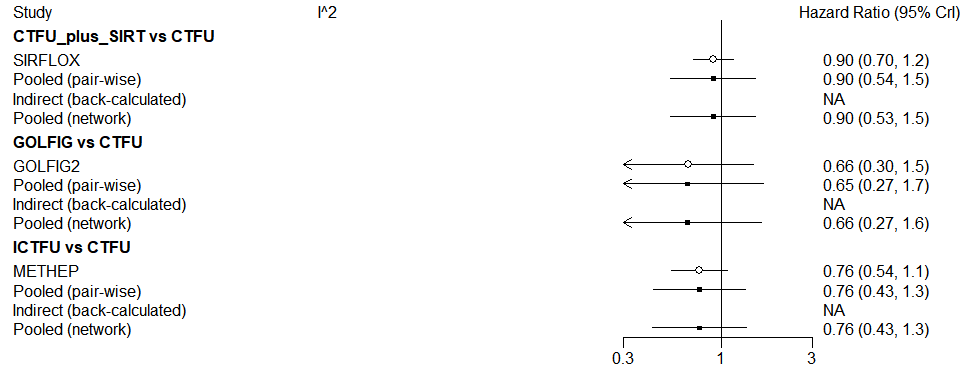


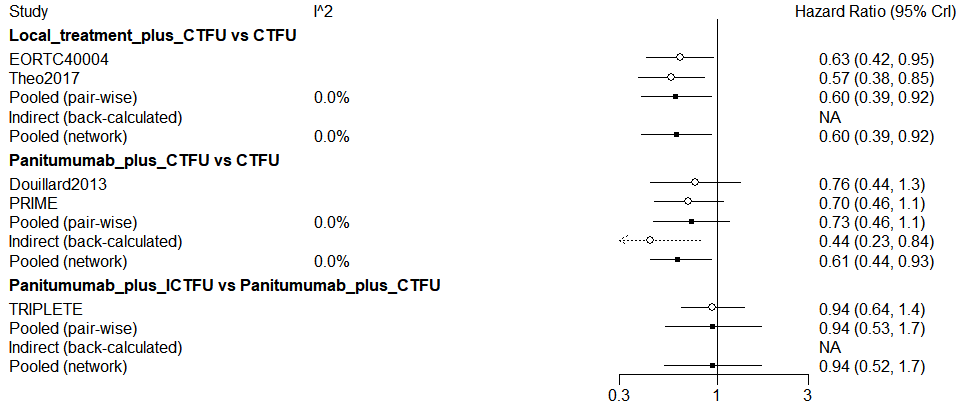


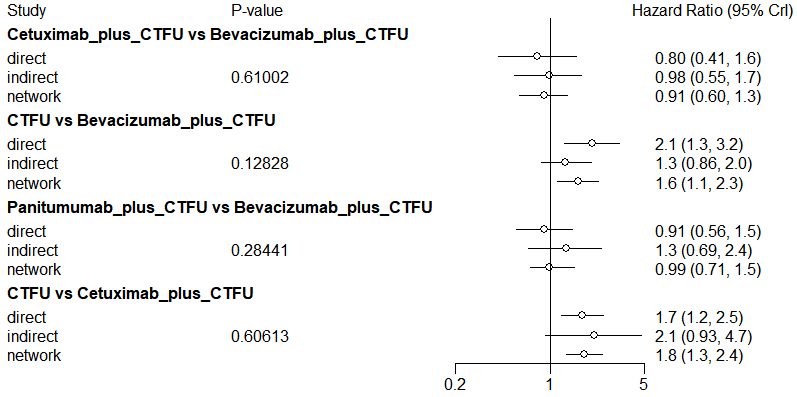


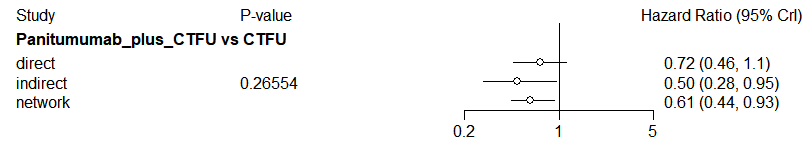


1. OS for Maintenance Treatment


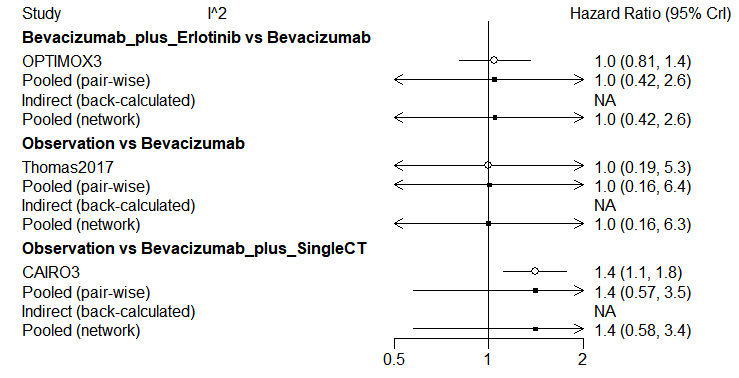


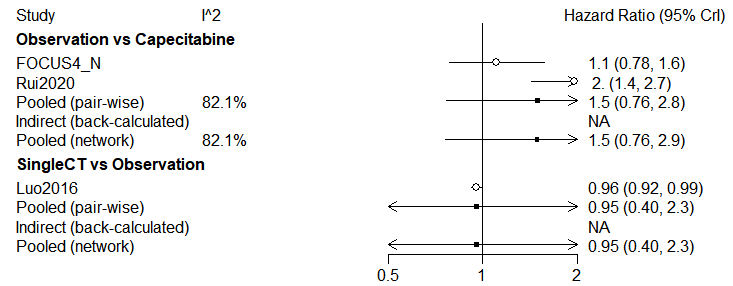


1. PFS for Maintenance Treatment


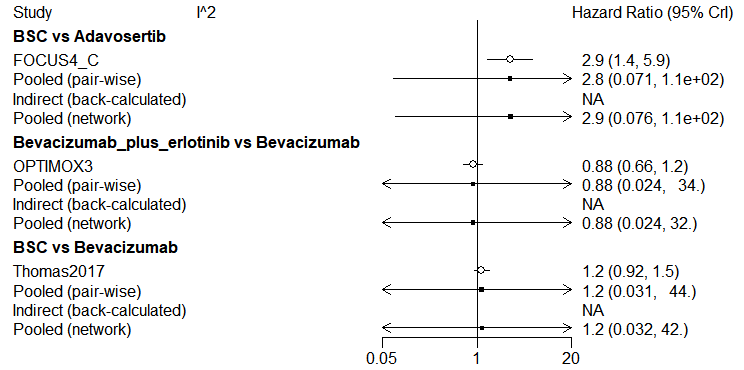


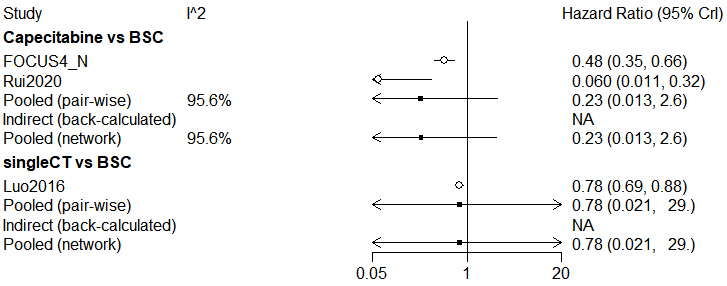


1. AE for Maintenance Treatment


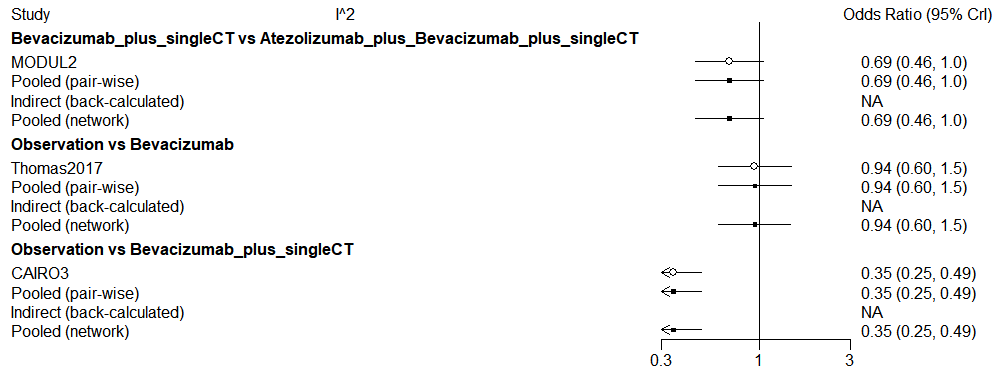


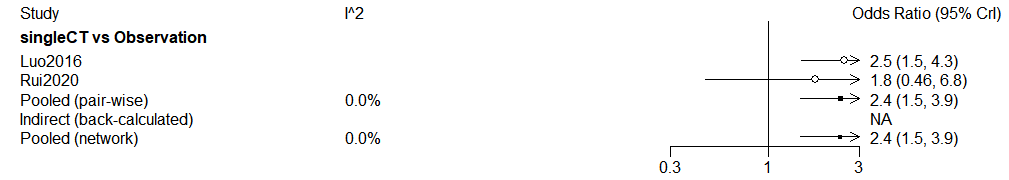


1. AE for First-line Treatment


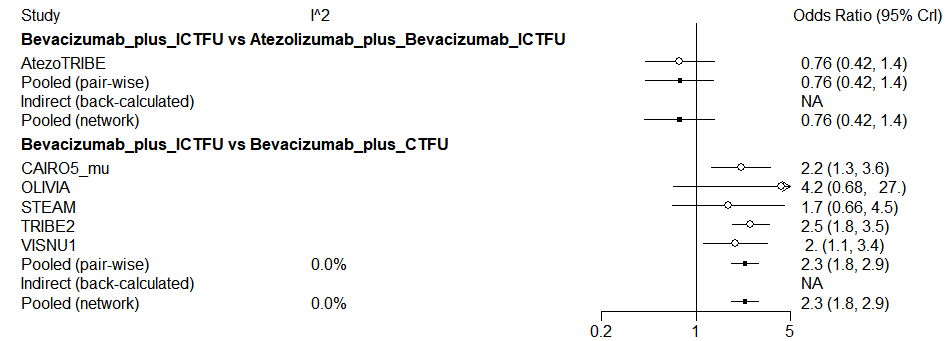


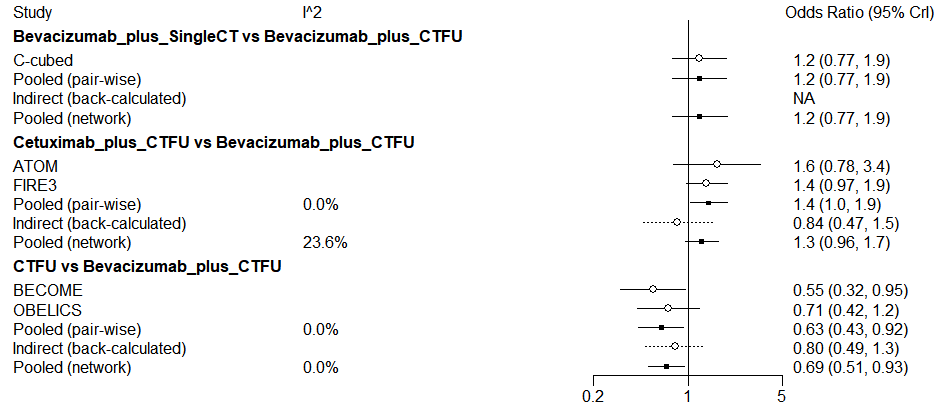


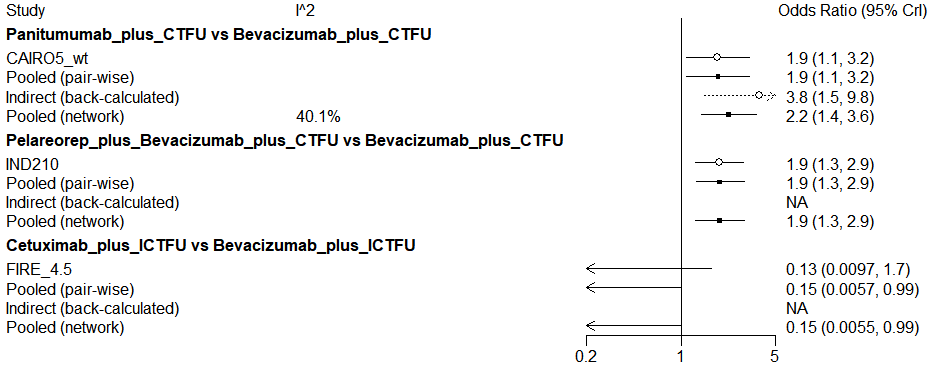


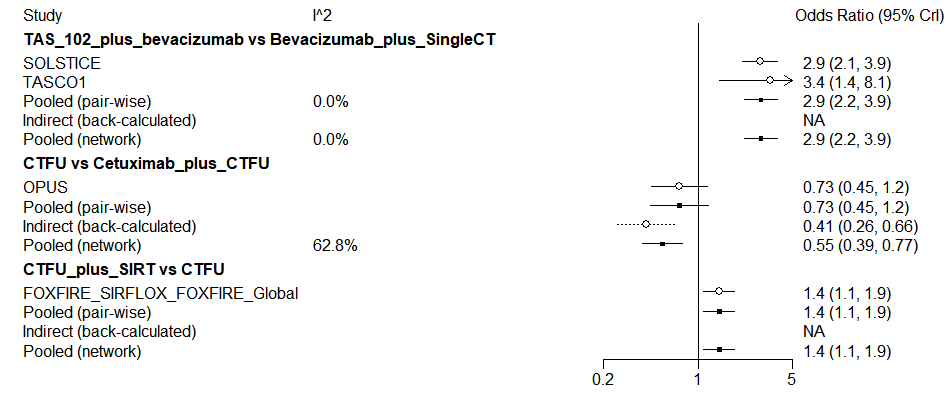


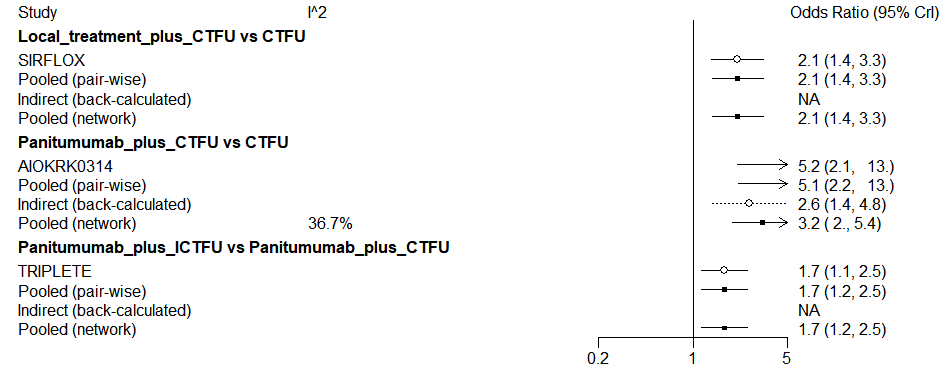


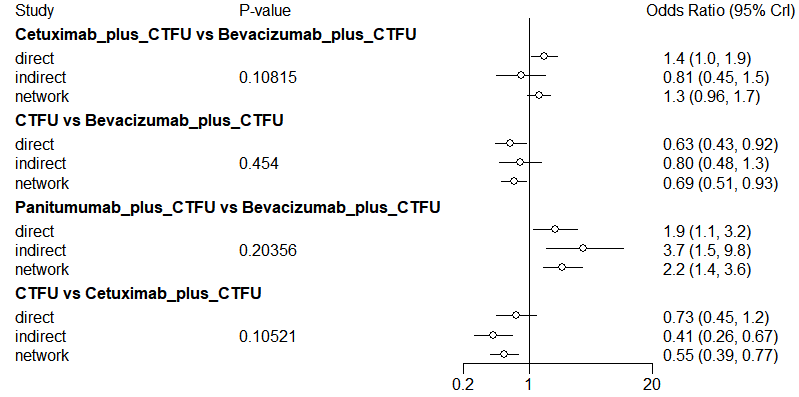


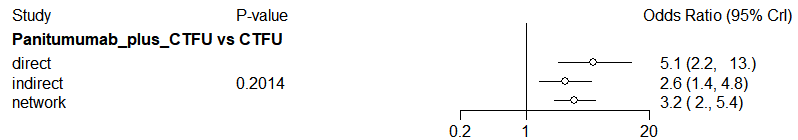


1. ORR for First-line Treatment


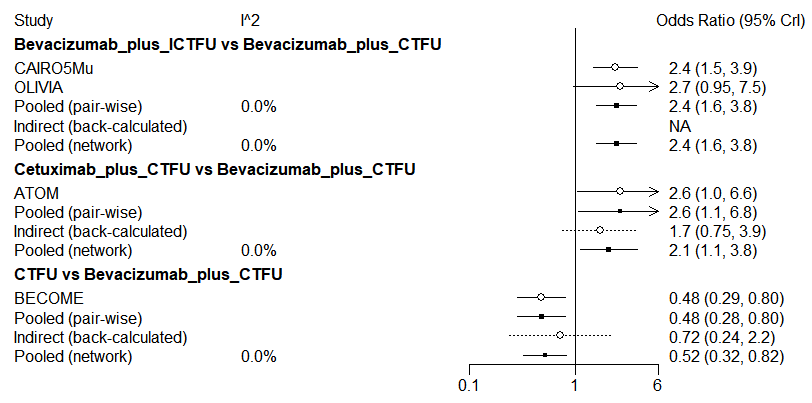


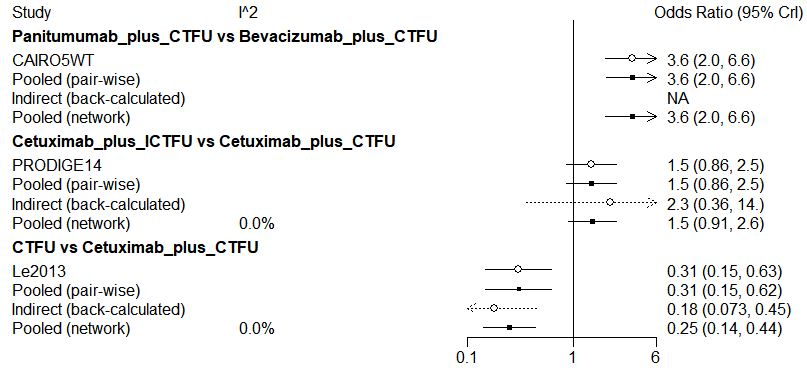


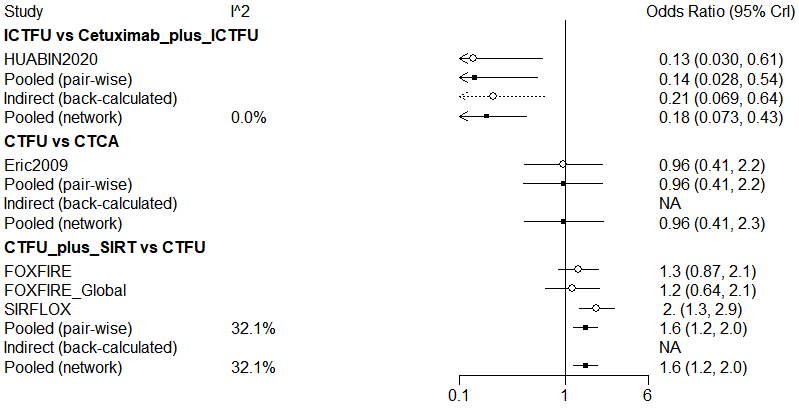


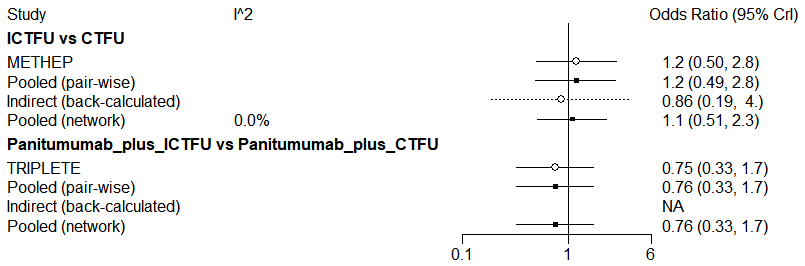


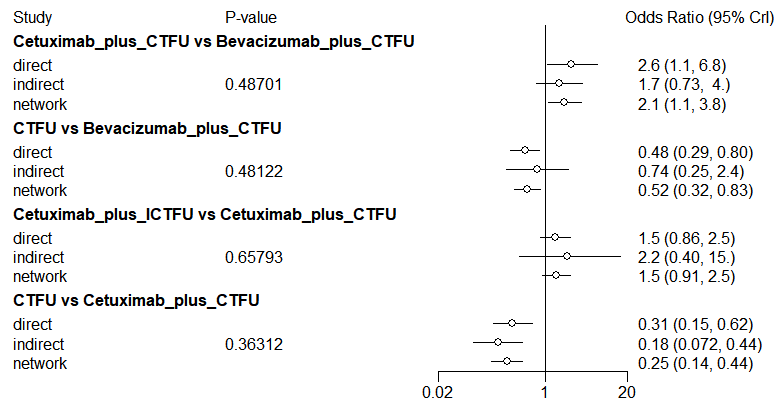


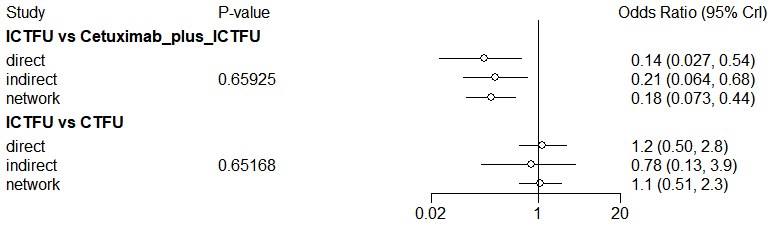


1. R0 resection rate for First-line Treatment


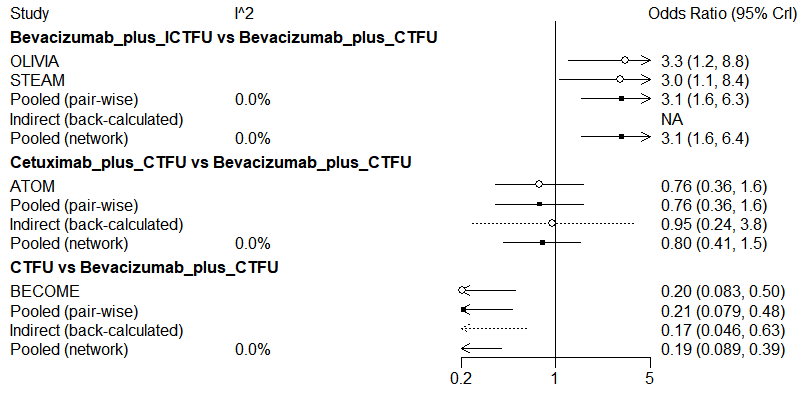


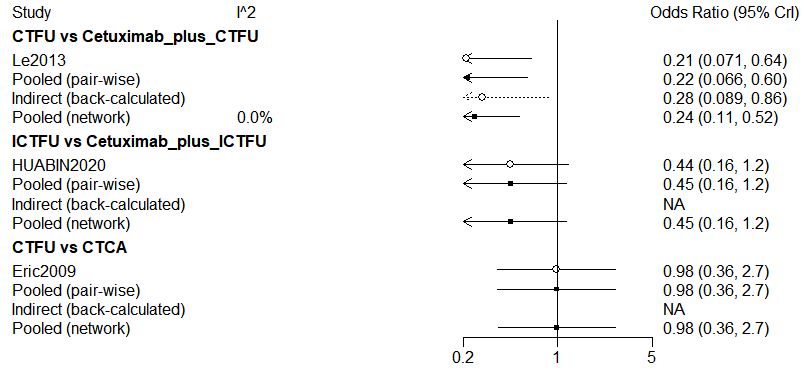


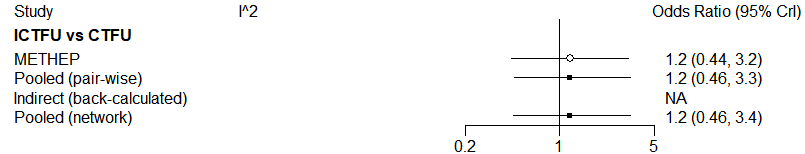

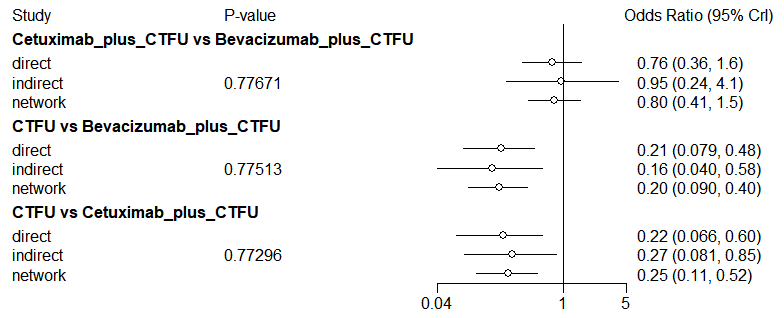


1. OS for First-line Liver Metastases (RAS/RAF Wild-type)


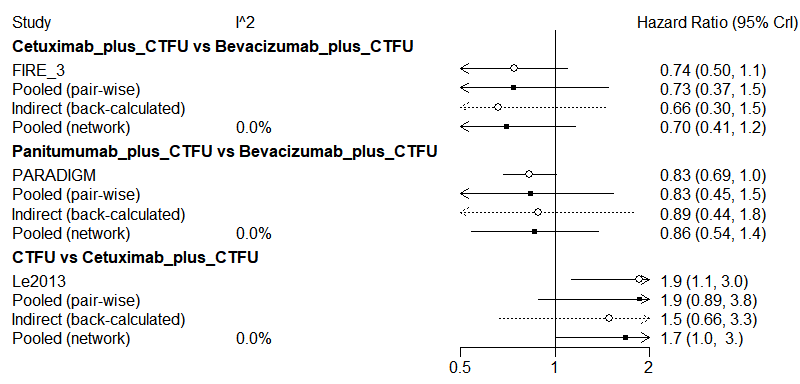


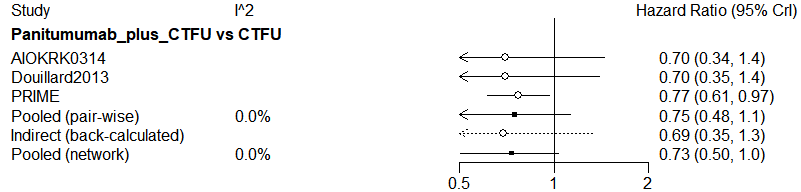


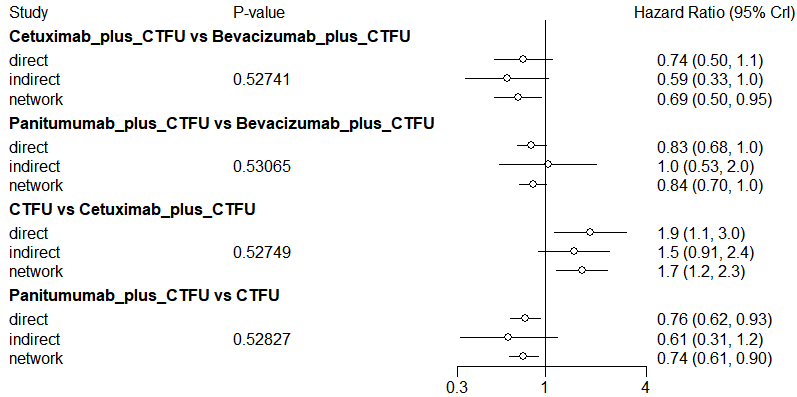


1. OS for First-line Liver Metastases (Wild-type Patients)


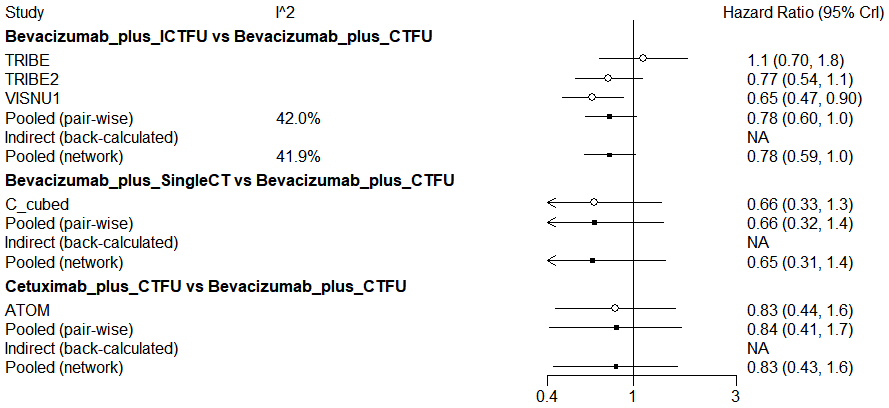


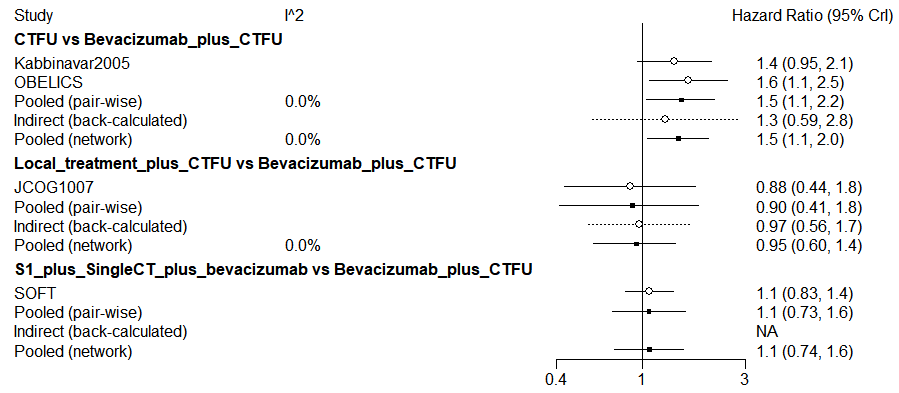


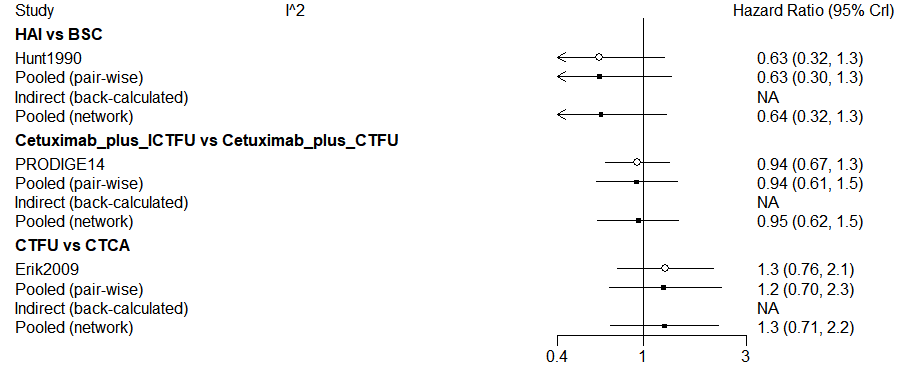


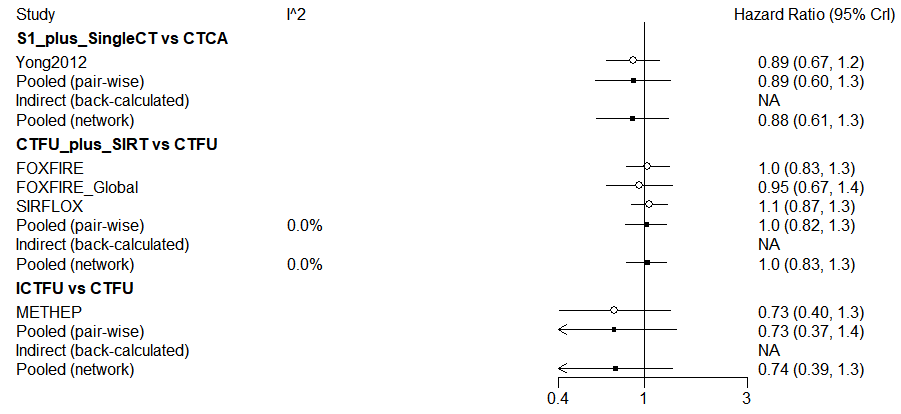


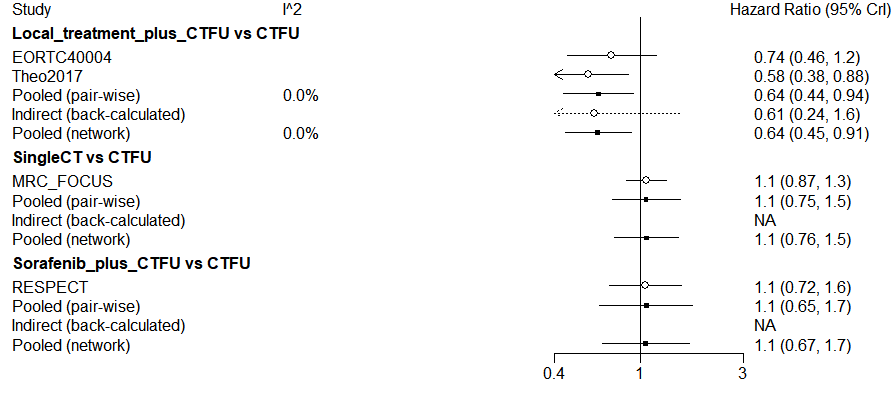


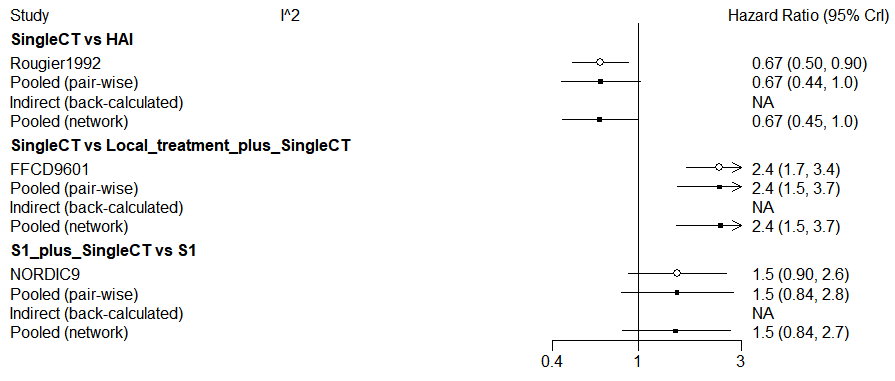


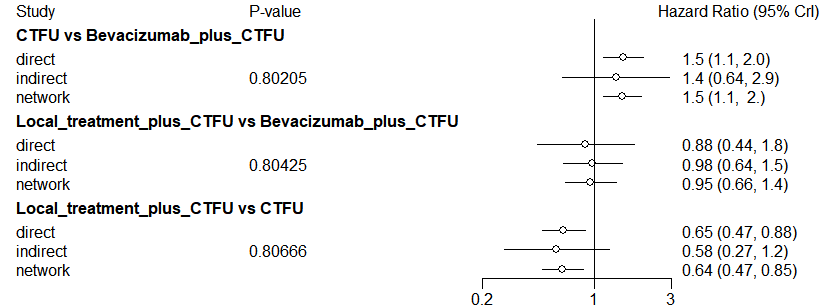


1. PFS for First-line Liver Metastases (RAS/RAF Wild-type)


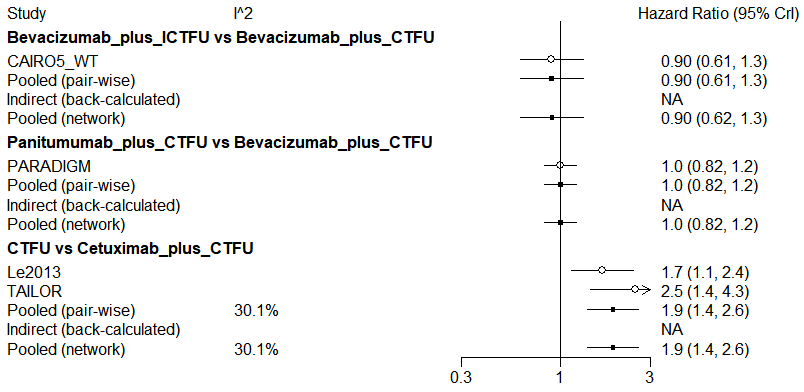


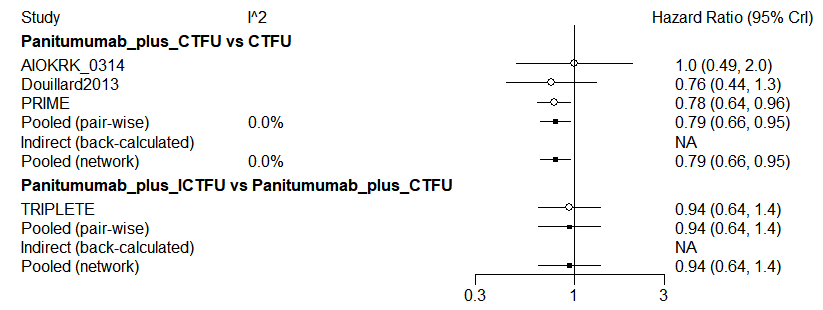


1. PFS for First-line Liver Metastases (Wild-type Patients)


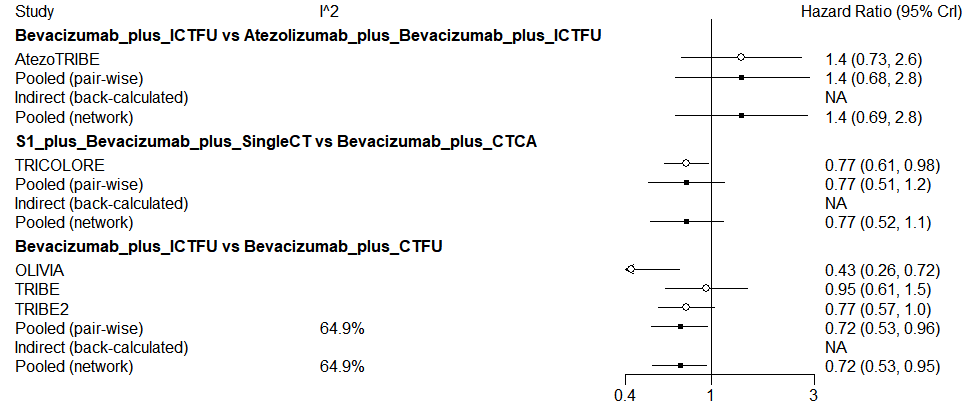


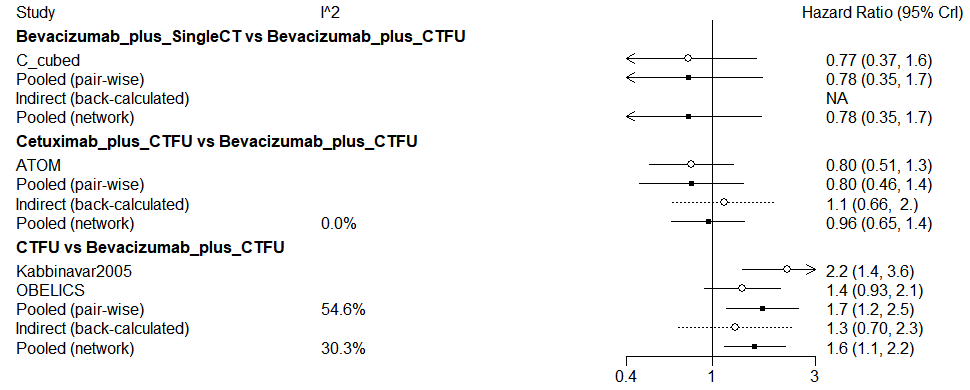


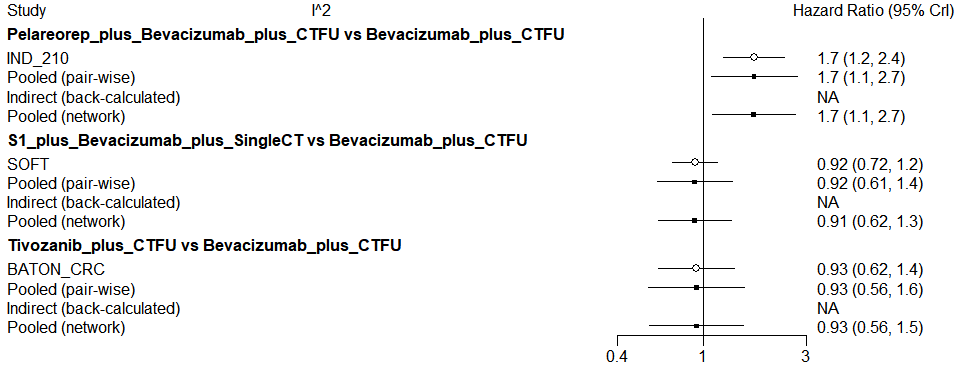


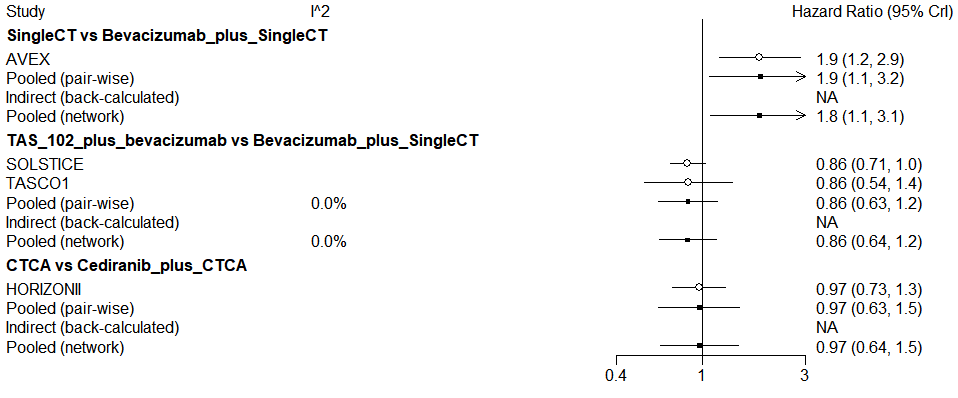


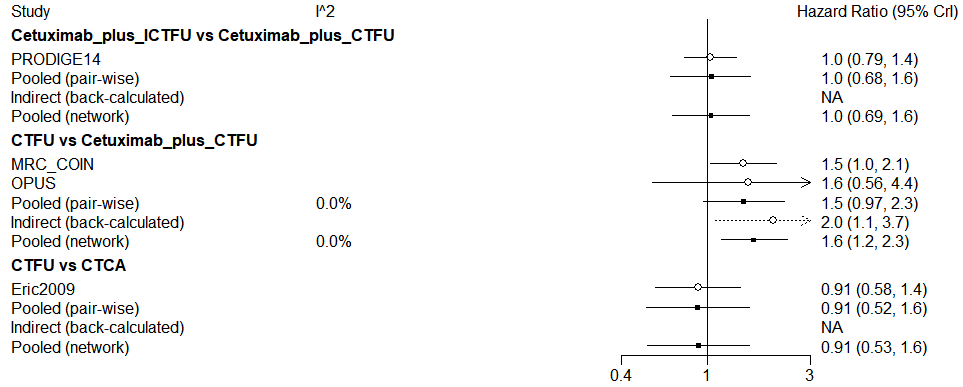


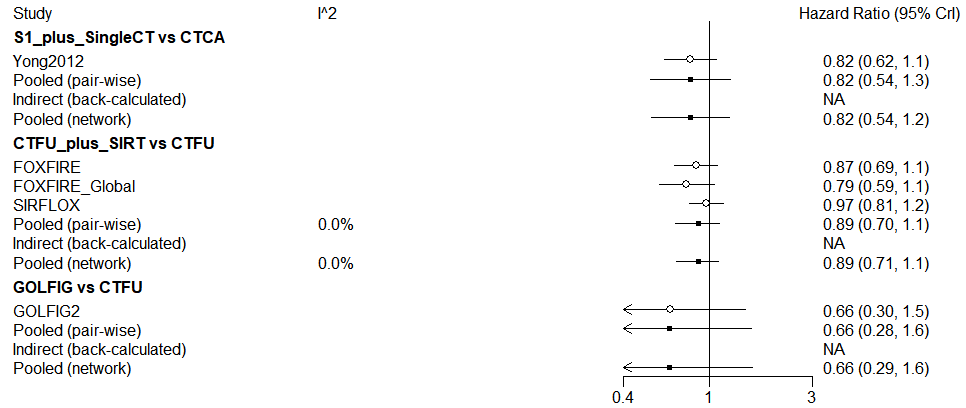


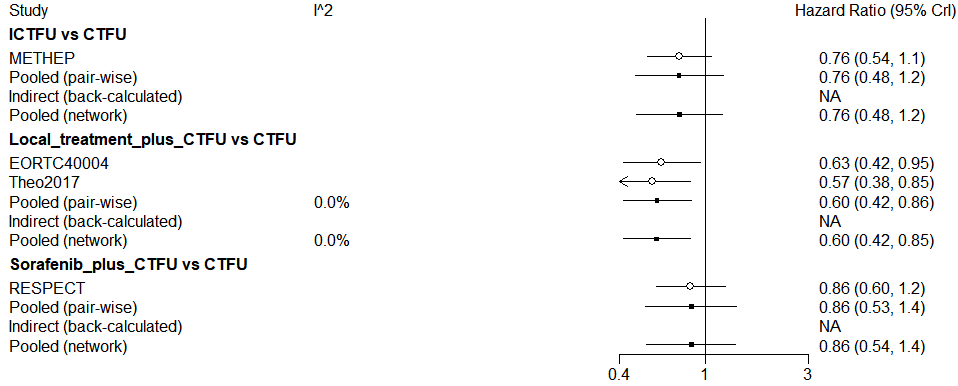


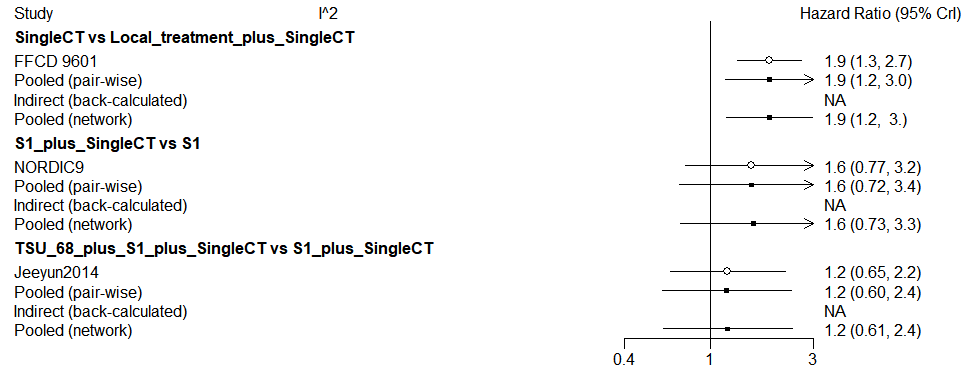


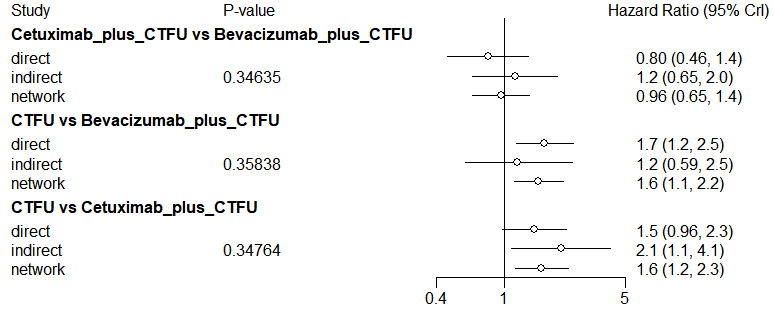


# Supplementary File 12. Brooks-Gelman-Rubin diagnostic

1. OS for First-line Liver Metastases

1. PFS for First-line Liver Metastases

1. OS for First-line Multiple-organ Metastases

1. PFS for First-line Multiple-organ Metastases

1. OS for First-line Liver-limited Metastases

1. PFS for First-line Liver-limited Metastases

1. OS for Maintenance Treatment

1. PFS for Maintenance Treatment

1. AE for Maintenance Treatment

1. AE for First-line Treatment

1. R0 resection rate for First-line Treatment

**References**

[1]. Bond, M., et al., First-line systemic treatment strategies in patients with initially unresectable colorectal cancer liver metastases (CAIRO5): an open-label, multicentre, randomised, controlled, phase 3 study from the Dutch Colorectal Cancer Group. Lancet Oncol, 2023. 24(7): p. 757-771.

[2]. Watanabe, J., et al., Panitumumab vs Bevacizumab Added to Standard First-line Chemotherapy and Overall Survival Among Patients With RAS Wild-type, Left-Sided Metastatic Colorectal Cancer: A Randomized Clinical Trial. JAMA, 2023. 329(15): p. 1271-1282.

[3]. Modest, D.P., et al., FOLFOX plus panitumumab or FOLFOX alone as additive therapy following R0/1 resection of RAS wild-type colorectal cancer liver metastases - The PARLIM trial (AIO KRK 0314). Eur J Cancer, 2022. 173: p. 297-306.

[4]. Rossini, D., et al., Upfront Modified Fluorouracil, Leucovorin, Oxaliplatin, and Irinotecan Plus Panitumumab Versus Fluorouracil, Leucovorin, and Oxaliplatin Plus Panitumumab for Patients With RAS/BRAF Wild-Type Metastatic Colorectal Cancer: The Phase III TRIPLETE Study by GONO. J Clin Oncol, 2022. 40(25): p. 2878-2888.

[5]. Antoniotti, C., et al., Upfront FOLFOXIRI plus bevacizumab with or without atezolizumab in the treatment of patients with metastatic colorectal cancer (AtezoTRIBE): a multicentre, open-label, randomised, controlled, phase 2 trial. Lancet Oncol, 2022. 23(7): p. 876-887.

[6]. Seligmann, J.F., et al., Inhibition of WEE1 Is Effective in TP53- and RAS-Mutant Metastatic Colorectal Cancer: A Randomized Trial (FOCUS4-C) Comparing Adavosertib (AZD1775) With Active Monitoring. J Clin Oncol, 2021. 39(33): p. 3705-3715.

[7]. Adams, R.A., et al., Capecitabine Versus Active Monitoring in Stable or Responding Metastatic Colorectal Cancer After 16 Weeks of First-Line Therapy: Results of the Randomized FOCUS4-N Trial. J Clin Oncol, 2021. 39(33): p. 3693-3704.

[8]. Avallone, A., et al., Effect of Bevacizumab in Combination With Standard Oxaliplatin-Based Regimens in Patients With Metastatic Colorectal Cancer: A Randomized Clinical Trial. JAMA Netw Open, 2021. 4(7): p. e2118475.

[9]. Kanemitsu, Y., et al., Primary Tumor Resection Plus Chemotherapy Versus Chemotherapy Alone for Colorectal Cancer Patients With Asymptomatic, Synchronous Unresectable Metastases (JCOG1007; iPACS): A Randomized Clinical Trial. J Clin Oncol, 2021. 39(10): p. 1098-1107.

[10]. Geng, R., et al., Metronomic capecitabine as maintenance treatment after first line induction with XELOX for metastatic colorectal cancer patients. Medicine (Baltimore), 2020. 99(51): p. e23719.

[11]. Heinemann, V., et al., FOLFIRI plus cetuximab versus FOLFIRI plus bevacizumab as first-line treatment for patients with metastatic colorectal cancer (FIRE-3): a randomised, open-label, phase 3 trial. Lancet Oncol, 2014. 15(10): p. 1065-75.

[12]. Stintzing, S., et al., FOLFIRI plus cetuximab versus FOLFIRI plus bevacizumab for metastatic colorectal cancer (FIRE-3): a post-hoc analysis of tumour dynamics in the final RAS wild-type subgroup of this randomised open-label phase 3 trial. Lancet Oncol, 2016. 17(10): p. 1426-1434.

[13]. Aranda, E., et al., FOLFOXIRI plus bevacizumab versus FOLFOX plus bevacizumab for patients with metastatic colorectal cancer and ≥3 circulating tumour cells: the randomised phase III VISNÚ-1 trial. ESMO Open, 2020. 5(6): p. e000944.

[14]. Cremolini, C., et al., Upfront FOLFOXIRI plus bevacizumab and reintroduction after progression versus mFOLFOX6 plus bevacizumab followed by FOLFIRI plus bevacizumab in the treatment of patients with metastatic colorectal cancer (TRIBE2): a multicentre, open-label, phase 3, randomised, controlled trial. Lancet Oncol, 2020. 21(4): p. 497-507.

[15]. Winther, S.B., et al., Reduced-dose combination chemotherapy (S-1 plus oxaliplatin) versus full-dose monotherapy (S-1) in older vulnerable patients with metastatic colorectal cancer (NORDIC9): a randomised, open-label phase 2 trial. Lancet Gastroenterol Hepatol, 2019. 4(5): p. 376-388.

[16]. Qin, S., et al., Efficacy and Tolerability of First-Line Cetuximab Plus Leucovorin, Fluorouracil, and Oxaliplatin (FOLFOX-4) Versus FOLFOX-4 in Patients With RAS Wild-Type Metastatic Colorectal Cancer: The Open-Label, Randomized, Phase III TAILOR Trial. J Clin Oncol, 2018. 36(30): p. 3031-3039.

[17]. Jonker, D.J., et al., A Randomized Phase II Study of FOLFOX6/Bevacizumab With or Without Pelareorep in Patients With Metastatic Colorectal Cancer: IND.210, a Canadian Cancer Trials Group Trial. Clin Colorectal Cancer, 2018. 17(3): p. 231-239.e7.

[18]. Aparicio, T., et al., Bevacizumab Maintenance Versus No Maintenance During Chemotherapy-Free Intervals in Metastatic Colorectal Cancer: A Randomized Phase III Trial (PRODIGE 9). J Clin Oncol, 2018. 36(7): p. 674-681.

[19]. Yamada, Y., et al., S-1 and irinotecan plus bevacizumab versus mFOLFOX6 or CapeOX plus bevacizumab as first-line treatment in patients with metastatic colorectal cancer (TRICOLORE): a randomized, open-label, phase III, noninferiority trial. Ann Oncol, 2018. 29(3): p. 624-631.

[20]. Denda, T., et al., Combination therapy of bevacizumab with either S-1 and irinotecan or mFOLFOX6/CapeOX as first-line treatment of metastatic colorectal cancer (TRICOLORE): Exploratory analysis of RAS status and primary tumour location in a randomised, open-label, phase III, non-inferiority trial. Eur J Cancer, 2021. 154: p. 296-306.

[21]. Simkens, L.H., et al., Maintenance treatment with capecitabine and bevacizumab in metastatic colorectal cancer (CAIRO3): a phase 3 randomised controlled trial of the Dutch Colorectal Cancer Group. Lancet, 2015. 385(9980): p. 1843-52.

[22]. Goey, K., et al., Maintenance treatment with capecitabine and bevacizumab versus observation in metastatic colorectal cancer: updated results and molecular subgroup analyses of the phase 3 CAIRO3 study. Ann Oncol, 2017. 28(9): p. 2128-2134.

[23]. Benson, A.R., et al., BATON-CRC: A Phase II Randomized Trial Comparing Tivozanib Plus mFOLFOX6 with Bevacizumab Plus mFOLFOX6 in Stage IV Metastatic Colorectal Cancer. Clin Cancer Res, 2016. 22(20): p. 5058-5067.

[24]. Luo, H.Y., et al., Single-agent capecitabine as maintenance therapy after induction of XELOX (or FOLFOX) in first-line treatment of metastatic colorectal cancer: randomized clinical trial of efficacy and safety. Ann Oncol, 2016. 27(6): p. 1074-1081.

[25]. van Hazel, G.A., et al., SIRFLOX: Randomized Phase III Trial Comparing First-Line mFOLFOX6 (Plus or Minus Bevacizumab) Versus mFOLFOX6 (Plus or Minus Bevacizumab) Plus Selective Internal Radiation Therapy in Patients With Metastatic Colorectal Cancer. J Clin Oncol, 2016. 34(15): p. 1723-31.

[26]. Aparicio, T., et al., Randomized phase III trial in elderly patients comparing LV5FU2 with or without irinotecan for first-line treatment of metastatic colorectal cancer (FFCD 2001-02). Ann Oncol, 2016. 27(1): p. 121-7.

[27]. Tournigand, C., et al., Bevacizumab with or without erlotinib as maintenance therapy in patients with metastatic colorectal cancer (GERCOR DREAM; OPTIMOX3): a randomised, open-label, phase 3 trial. Lancet Oncol, 2015. 16(15): p. 1493-1505.

[28]. Loupakis, F., et al., Initial therapy with FOLFOXIRI and bevacizumab for metastatic colorectal cancer. N Engl J Med, 2014. 371(17): p. 1609-18.

[29]. Cremolini, C., et al., FOLFOXIRI plus bevacizumab versus FOLFIRI plus bevacizumab as first-line treatment of patients with metastatic colorectal cancer: updated overall survival and molecular subgroup analyses of the open-label, phase 3 TRIBE study. Lancet Oncol, 2015. 16(13): p. 1306-15.

[30]. Bokemeyer, C., et al., Fluorouracil, leucovorin, and oxaliplatin with and without cetuximab in the first-line treatment of metastatic colorectal cancer. J Clin Oncol, 2009. 27(5): p. 663-71.

[31]. Bokemeyer, C., et al., FOLFOX4 plus cetuximab treatment and RAS mutations in colorectal cancer. Eur J Cancer, 2015. 51(10): p. 1243-52.

[32]. Bokemeyer, C., et al., Efficacy according to biomarker status of cetuximab plus FOLFOX-4 as first-line treatment for metastatic colorectal cancer: the OPUS study. Ann Oncol, 2011. 22(7): p. 1535-1546.

[33]. Wasan, H.S., et al., First-line selective internal radiotherapy plus chemotherapy versus chemotherapy alone in patients with liver metastases from colorectal cancer (FOXFIRE, SIRFLOX, and FOXFIRE-Global): a combined analysis of three multicentre, randomised, phase 3 trials. Lancet Oncol, 2017. 18(9): p. 1159-1171.

[34]. Stintzing, S., et al., FOLFOXIRI Plus Cetuximab or Bevacizumab as First-Line Treatment of BRAF(V600E)-Mutant Metastatic Colorectal Cancer: The Randomized Phase II FIRE-4.5 (AIO KRK0116) Study. J Clin Oncol, 2023. 41(25): p. 4143-4153.

[35]. Cassidy, J., et al., Randomized phase III study of capecitabine plus oxaliplatin compared with fluorouracil/folinic acid plus oxaliplatin as first-line therapy for metastatic colorectal cancer. J Clin Oncol, 2008. 26(12): p. 2006-12.

[36]. Seymour, M.T., et al., Different strategies of sequential and combination chemotherapy for patients with poor prognosis advanced colorectal cancer (MRC FOCUS): a randomised controlled trial. Lancet, 2007. 370(9582): p. 143-152.

[37]. Ychou, M., et al., Chemotherapy (doublet or triplet) plus targeted therapy by RAS status as conversion therapy in colorectal cancer patients with initially unresectable liver-only metastases. The UNICANCER PRODIGE-14 randomised clinical trial. Br J Cancer, 2022. 126(9): p. 1264-1270.

[38]. Hu, H., et al., Modified FOLFOXIRI With or Without Cetuximab as Conversion Therapy in Patients with RAS/BRAF Wild-Type Unresectable Liver Metastases Colorectal Cancer: The FOCULM Multicenter Phase II Trial. Oncologist, 2021. 26(1): p. e90-e98.

[39]. Oki, E., et al., Randomised phase II trial of mFOLFOX6 plus bevacizumab versus mFOLFOX6 plus cetuximab as first-line treatment for colorectal liver metastasis (ATOM trial). Br J Cancer, 2019. 121(3): p. 222-229.

[40]. Ruers, T., et al., Local Treatment of Unresectable Colorectal Liver Metastases: Results of a Randomized Phase II Trial. J Natl Cancer Inst, 2017. 109(9).

[41]. Gruenberger, T., et al., Bevacizumab plus mFOLFOX-6 or FOLFOXIRI in patients with initially unresectable liver metastases from colorectal cancer: the OLIVIA multinational randomised phase II trial. Ann Oncol, 2015. 26(4): p. 702-708.

[42]. Ychou, M., et al., A randomized phase II trial of three intensified chemotherapy regimens in first-line treatment of colorectal cancer patients with initially unresectable or not optimally resectable liver metastases. The METHEP trial. Ann Surg Oncol, 2013. 20(13): p. 4289-97.

[43]. Tang, W., et al., Bevacizumab Plus mFOLFOX6 Versus mFOLFOX6 Alone as First-Line Treatment for RAS Mutant Unresectable Colorectal Liver-Limited Metastases: The BECOME Randomized Controlled Trial. J Clin Oncol, 2020. 38(27): p. 3175-3184.

[44]. Ye, L.C., et al., Randomized controlled trial of cetuximab plus chemotherapy for patients with KRAS wild-type unresectable colorectal liver-limited metastases. J Clin Oncol, 2013. 31(16): p. 1931-8.

[45]. Ferrand, F., et al., Impact of primary tumour resection on survival of patients with colorectal cancer and synchronous metastases treated by chemotherapy: results from the multicenter, randomised trial Fédération Francophone de Cancérologie Digestive 9601. Eur J Cancer, 2013. 49(1): p. 90-7.

[46]. Ruers, T., et al., Radiofrequency ablation combined with systemic treatment versus systemic treatment alone in patients with non-resectable colorectal liver metastases: a randomized EORTC Intergroup phase II study (EORTC 40004). Ann Oncol, 2012. 23(10): p. 2619-2626.

[47]. Ducreux, M., et al., Sequential versus combination chemotherapy for the treatment of advanced colorectal cancer (FFCD 2000-05): an open-label, randomised, phase 3 trial. Lancet Oncol, 2011. 12(11): p. 1032-44.

[48]. Skof, E., et al., Capecitabine plus Irinotecan (XELIRI regimen) compared to 5-FU/LV plus Irinotecan (FOLFIRI regimen) as neoadjuvant treatment for patients with unresectable liver-only metastases of metastatic colorectal cancer: a randomised prospective phase II trial. BMC Cancer, 2009. 9: p. 120.

[49]. Rougier, P., et al., Hepatic arterial infusion of floxuridine in patients with liver metastases from colorectal carcinoma: long-term results of a prospective randomized trial. J Clin Oncol, 1992. 10(7): p. 1112-8.

[50]. Hunt, T.M., et al., Prospective randomized controlled trial of hepatic arterial embolization or infusion chemotherapy with 5-fluorouracil and degradable starch microspheres for colorectal liver metastases. Br J Surg, 1990. 77(7): p. 779-82.

[51]. Douillard, J.Y., et al., Final results from PRIME: randomized phase III study of panitumumab with FOLFOX4 for first-line treatment of metastatic colorectal cancer. Ann Oncol, 2014. 25(7): p. 1346-1355.

[52]. Douillard, J.Y., et al., Randomized, phase III trial of panitumumab with infusional fluorouracil, leucovorin, and oxaliplatin (FOLFOX4) versus FOLFOX4 alone as first-line treatment in patients with previously untreated metastatic colorectal cancer: the PRIME study. J Clin Oncol, 2010. 28(31): p. 4697-705.

[53]. Lee, J., et al., A phase II open-label randomized multicenter trial of TSU-68 in combination with S-1 and oxaliplatin versus S-1 in combination with oxaliplatin in patients with metastatic colorectal cancer. Invest New Drugs, 2014. 32(3): p. 561-8.

[54]. Correale, P., et al., Gemcitabine, oxaliplatin, levofolinate, 5-fluorouracil, granulocyte-macrophage colony-stimulating factor, and interleukin-2 (GOLFIG) versus FOLFOX chemotherapy in metastatic colorectal cancer patients: the GOLFIG-2 multicentric open-label randomized phase III trial. J Immunother, 2014. 37(1): p. 26-35.

[55]. Yamada, Y., et al., Leucovorin, fluorouracil, and oxaliplatin plus bevacizumab versus S-1 and oxaliplatin plus bevacizumab in patients with metastatic colorectal cancer (SOFT): an open-label, non-inferiority, randomised phase 3 trial. Lancet Oncol, 2013. 14(13): p. 1278-86.

[56]. Baba, H., et al., S-1 and oxaliplatin (SOX) plus bevacizumab versus mFOLFOX6 plus bevacizumab as first-line treatment for patients with metastatic colorectal cancer: updated overall survival analyses of the open-label, non-inferiority, randomised phase III: SOFT study. ESMO Open, 2017. 2(1): p. e000135.

[57]. Douillard, J.Y., et al., Panitumumab-FOLFOX4 treatment and RAS mutations in colorectal cancer. N Engl J Med, 2013. 369(11): p. 1023-34.

[58]. Tabernero, J., et al., Sorafenib in combination with oxaliplatin, leucovorin, and fluorouracil (modified FOLFOX6) as first-line treatment of metastatic colorectal cancer: the RESPECT trial. Clin Cancer Res, 2013. 19(9): p. 2541-50.

[59]. Hong, Y.S., et al., S-1 plus oxaliplatin versus capecitabine plus oxaliplatin for first-line treatment of patients with metastatic colorectal cancer: a randomised, non-inferiority phase 3 trial. Lancet Oncol, 2012. 13(11): p. 1125-32.

[60]. Hoff, P.M., et al., Cediranib plus FOLFOX/CAPOX versus placebo plus FOLFOX/CAPOX in patients with previously untreated metastatic colorectal cancer: a randomized, double-blind, phase III study (HORIZON II). J Clin Oncol, 2012. 30(29): p. 3596-603.

[61]. Maughan, T.S., et al., Addition of cetuximab to oxaliplatin-based first-line combination chemotherapy for treatment of advanced colorectal cancer: results of the randomised phase 3 MRC COIN trial. Lancet, 2011. 377(9783): p. 2103-14.

[62]. Kabbinavar, F.F., et al., Addition of bevacizumab to bolus fluorouracil and leucovorin in first-line metastatic colorectal cancer: results of a randomized phase II trial. J Clin Oncol, 2005. 23(16): p. 3697-705.
